# Isolation and Structure Determination of Drought-Induced Multihexose Benzoxazinoids from Maize (*Zea mays*)

**DOI:** 10.1021/acs.jafc.3c09141

**Published:** 2024-02-09

**Authors:** Sylvain Sutour, Van Cong Doan, Pierre Mateo, Tobias Züst, Ella Raymonde Hartmann, Gaétan Glauser, Christelle Aurélie Maud Robert

**Affiliations:** † Neuchâtel Platform of Analytical Chemistry, University of Neuchâtel, Neuchâtel 2000, Switzerland; ‡ Institute of Plant Sciences, University of Bern, Bern 3013, Switzerland; § Oeschger Centre for Climate Change Research (OCCR), University of Bern, Bern 3012, Switzerland; ∥ Plant Physiology Unit, The Department of Life Sciences and Systems Biology of the University of Turin, Via Accademia Albertina 13, Torino 10123, Italy; ⊥ Department of Systematic and Evolutionary Botany, University of Zürich, Zürich 8008, Switzerland

**Keywords:** maize, drought, NMR, multihexose benzoxazinoids, DIMBOA dihexose, DIMBOA trihexose

## Abstract

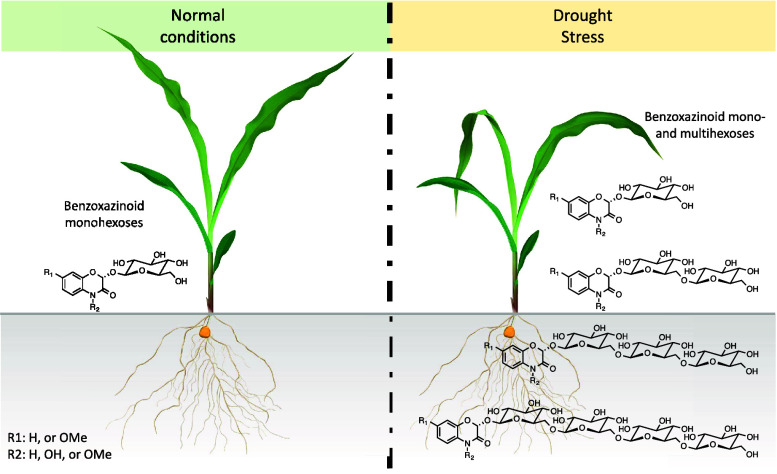

Benzoxazinoids (BXDs) are plant specialized metabolites exerting a pivotal role in plant nutrition, allelopathy, and defenses. Multihexose benzoxazinoids were previously observed in cereal-based food products such as whole-grain bread. However, their production in plants and exact structure have not been fully elucidated. In this study, we showed that drought induced the production of di-, tri-, and even tetrahexose BXDs in maize roots and leaves. We performed an extensive nuclear magnetic resonance study and elucidated the nature and linkage of the sugar units, which were identified as gentiobiose units β-linked (1″ → 6′) for the dihexoses and (1″ → 6′)/(1‴ → 6″) for the trihexoses. Drought induced the production of DIMBOA-2Glc, DIMBOA-3Glc, HMBOA-2Glc, HMBOA-3Glc, and HDMBOA-2Glc. The induction was common among several maize lines and the strongest in seven-day-old seedlings. This work provides ground to further characterize the BXD synthetic pathway, its relevance in maize-environment interactions, and its impact on human health.

## 1 Introduction

Benzoxazinoids (BXDs) are plant specialized metabolites that modulate plant nutrition, reproduction, interactions with microbes, and defenses against herbivores.^1^ They are commonly found in cereals and frequently recorded in wholegrain cereal products.^2−5^ BXDs can have cascading effects on food quality and human health, and their consumption is associated with anti-inflammatory and anticancer effects.^6,7^ Understanding the different molecular structures of BXDs is crucial for improving the reliability and robustness of analytical methods, for identifying their underlying biosynthesis genes, and ultimately, for comprehending their potential influence on human health.^8,9^

BXDs include benzoxazolinones with a 1,3-benzoxazol-2-one core structure and benzoxazinones, possessing a 1,4-benzoxazin-3-one scaffold. In rye, BXD species with no methoxy group on the aromatic ring, such as DIBOA and DIBOA-Glc, are dominant, whereas in maize, methylated forms such as DIMBOA and DIMBOA-Glc are predominant. In wheat, both species can be found in similar amounts.^10^ Multihexose BXDs, such as H(M)BOA and DI(M)BOA di-, tri-, and tetrahexoses, were reported in wheat, maize, and rye.^4,10−16^ The structures of dihexose BXDs were described using MS/MS fragmentation as H(M)BOA-Glc-Hex and DI(M)BOA-Glc-Hex.^4,8,10−14^ Consequently, the exact nature and configuration of the hexose units as well as the relative and absolute stereochemistry of multihexose BXDs have remained undefined.

BXDs can transition from plants to the human food supply, where they can impact human health. Food processing, such as watering/drying the seeds, or hydrothermal processing (HTP) of cereals were found to increase BXD levels, including dihexose BXDs, in processed products.^3,4,12^ For instance, rye-based products contain approximately 122 μg/g dry mass HBOA-Glc-Hex and 300 μg/g dry mass DIBOA-Glc-Hex.^4^ The impact of multihexose BXDs on human health remains unexplored, hindered by several factors, including the unavailability of purified compounds (attributable to their limited presence in healthy plants), a lack of structural characterization, and their omission from analytical methodologies.

Here, we demonstrate that drought enhances the production of multihexose BXDs in maize root and shoot tissues. We conducted an extensive nuclear magnetic resonance (NMR) study to determine the nature and configuration of the multihexose chains. We then characterized their induction patterns and kinetics in different maize tissues. Understanding the structure of these multihexose BXDs is pivotal for enabling their inclusion in analytical methods, enhancing our ability to monitor their presence and impact on food quality and human nutrition.

## 2 Materials and Methods

### 2.1 Biological Resources

Maize (*Zea mays*, varieties B73, CML277, Hp301, and Oh7B) were obtained from Maize GDB (https://maizegdb.org/data_center/phenotype#stocks) and bred in-house. The seeds were soaked in aqueous bleach (5%, Migros, CH) for 5 min, washed with distilled water, and then soaked in distilled water for one night before being placed in preweighed 100 mL plastic pots (4 cm diameter, 11.2 cm height; Platz GmbH Medizintechnik, Dorsten-Wulfen; DE) containing soil (40% sand, 35% silt, and 25% clay (Landerde, Ricoter, Aarberg; CHE)) with 23% moisture. The moisture level was controlled by drying soil in an oven (24 h, 75 °C) and adding the corresponding amount of water prior to planting. The plants were cultivated in a greenhouse with the following conditions: 14 h light (20 °C ± 2 °C), 10 h dark (18 °C ± 2 °C), 55% relative humidity, with 250 μmol·m^–2^·s^–1^ additional light supplied by Master GreenPower 600W 400 V E40 high-pressure sodium bulbs (Philips Lighting, CHE).

### 2.2 Drought Induction

After planting, the soil moisture was either kept at 23% v/v (ambient conditions) or left to decrease to 16% v/v (drought conditions, reached at day 4 after planting). Ambient and drought conditions were then maintained by weighing the pots and adding the required amounts of water daily. The drought moisture conditions were determined based on the projected RCP8.5 climate scenario, using a correlation between decrease precipitation levels and soil moisture levels as in refs (17−19). A 16.6% v/v moisture led to moderate drought symptoms in maize plants. All plants were harvested 10 days after sowing. Roots and shoots were harvested separately, flash-frozen in liquid nitrogen, and ground to a fine powder using a mortar and a pestle in the presence of liquid nitrogen.

### 2.3 Benzoxazinoid Analyses

The BXDs analysis was adapted from a previously described protocol.^20^ Aliquots of powder samples (100 mg) were mixed with 1 mL of a 70:30 mixture of methanol (HPLC grade) and water (milli-Q) containing 0.1% formic acid (LC-MS grade). All extracts were vortexed for 20 s and centrifuged at 13000 rpm for 20 min at 10 °C, and the supernatants were collected for LC-MS analyses. Where necessary, samples were diluted 1:10 prior to analysis.

The BXD profiling was performed with an Acquity i-Class UHPLC system equipped with a photodiode array detector (PDA) and a single quadrupole detector (QDa) with an electrospray source (Waters, USA). Gradient elution was performed on an Acquity BEH C18 (1.7 μm, 2.1 × 100 mm, Waters, USA) column maintained at 40 °C. The elution conditions were as follows: solvent A, H_2_O (Milli-Q) + 0.1% formic acid (LC-MS grade); solvent B, ACN (LC-MS grade) + 0.1% formic acid (LC-MS grade); flow rate, 0.4 mL/min; 0–3 min, linear gradient from 2 to 20% B; 3–6 min, linear gradient to 100% B; 6–8 min, 100% B; 8–10 min, 2% B. Absolute quantities of DIMBOA-2Glc, DIMBOA-3Glc and HMBOA-2Glc were determined through a semiquantitative method using standard curves of the corresponding pure monoglucoside DIMBOA-Glc for the two first ones and the aglucone HMBOA for the latter.

### 2.4 Crude Extract

Ten days after germination, root and shoot samples were gently washed in tap water, patted dry with paper, flash frozen in liquid nitrogen, and stored at −80 °C until further processing. The samples were ground manually with mortar and pestle in the presence of liquid nitrogen. Root and shoot samples were pooled to give 450 g of raw material. The extraction was performed in two equal batches (225 g each) as follows: the cold ground material was added to 1.5 L of MeOH and homogenized for 3 min with an immersion disperser (Polytron PT 10–35, Kinematica, CHE), followed by suction filtration through a P3 sintered glass filter equipped with two layers of filter paper. The filter cake was collected and suspended again in 1.5 L MeOH. After a second homogenization and a new filtration, the filtrates were combined and concentrated under reduced pressure on a rotary evaporator. Upon evaporation, a yellow-green sticky residue formed on the round-bottom flask walls. This residue was discarded, and the remaining aqueous phase was separated. The same procedure was repeated for the second batch of frozen ground material. The two aqueous residues obtained were pooled and lyophilized to provide a strong yellow crude dry extract. The presence of compounds **1**–**7** was verified by HPLC-MS prior to isolation.

### 2.5 Preparative Chromatography

The crude extract (1.8 g) was first fractionated by open column chromatography (CC) using 45 g of silica gel (63–200 μm, 60 Å) as the stationary phase. A step gradient elution consists of mixtures of dichloromethane-ethyl acetate, followed by ethyl acetate-methanol, and finally pure methanol yielded 11 fractions (F1–11). Compounds **1**–**7**, which were all present in F9 (126.8 mg), were further purified by semipreparative HPLC using a LC-20AR Shimadzu pump (Japan) connected to a UV–vis detector (Shimadzu, model SPD-20A) and a fraction collector FRC-40 (Shimadzu). A detailed description of the semipreparative conditions for each molecule is provided in Supporting Information SI1.

### 2.6 NMR Spectroscopy

NMR spectra were acquired on a Bruker Avance Neo Ascend 600 MHz (Bruker, Germany) in D_2_O. Standard pulse sequences were used to acquire 1D and 2D NMR analyses. HSQC and HMBC analyses were recorded in nonuniform sampling (NUS) mode with a sampling amount of 12.5%. Acetone was used as calibration standard (δ^1^H = 2.225 ppm; δ^13^C = 30.5 ppm) except for compounds **6** and **7**. In this case, spectra were calibrated on H-2′ (3.25 ppm) and C2′ (73.1 ppm). Acquired spectra were processed using the Mnova NMR software package (v.14.2.0, MestReLab Research S.L., Spain).

### 2.7 Statistical Analysis

All statistical analyses were conducted on Sigma Plot 14.5. The distribution and heteroscedasticity of the data residuals were assessed by using the Shapiro–Wilk and Brown–Forsythe tests. Two-way ANOVAs on ranks were conducted to assess the effects of drought and time on multihexose benzoxazinoid levels. Student *t-*tests and Mann–Whitney tests were performed to evaluate the impact of drought on individual compounds in maize.

## 3 Results and Discussion

### 3.1 Drought Promotes the Production of Putative Multihexose Benzoxazinones in Maize

**Figure 1:**
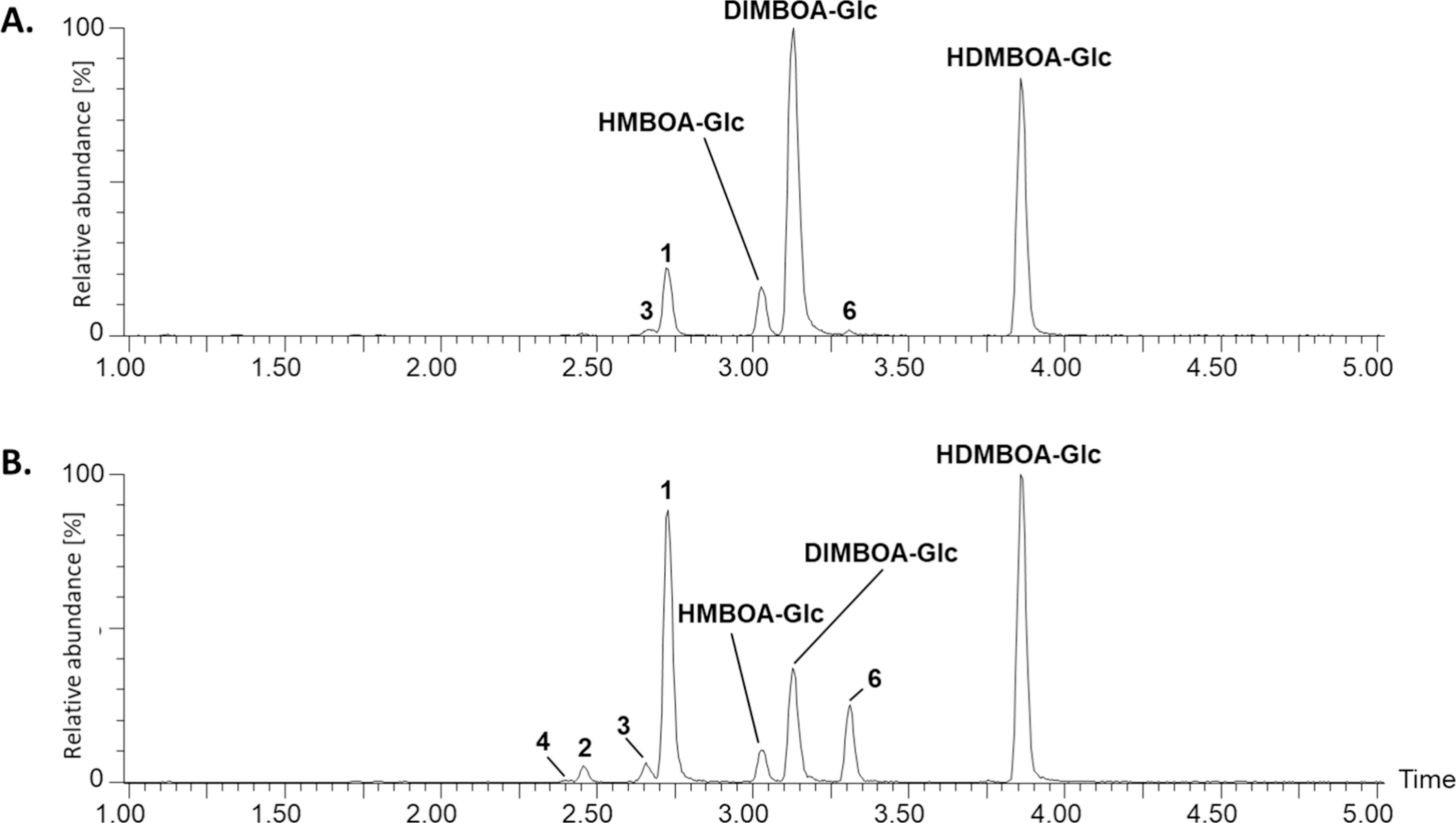
Drought induced putative multihexose benzoxazinoids (BXDs) in maize leaves. Base peak intensity (BPI) chromatograms of control (A) and drought-stressed (B) maize leaves. Putative multihexose benzoxazinoids (BXDs) are labeled as numbers in the chromatograms. (1) Putative DIMBOA-dihexose (*m*/*z* 534.1459), (2) putative DIMBOA-trihexose (*m*/*z* 696.1987), (3) putative HMBOA-dihexose (*m*/*z* 518.1510), (4) putative HMBOA-trihexose (*m*/*z* 680.2038), and (6) putative HDMBOA-dihexose (*m*/*z* 594.1670). *m*/*z*: mass-to-charge ratio.

**Figure 2:**
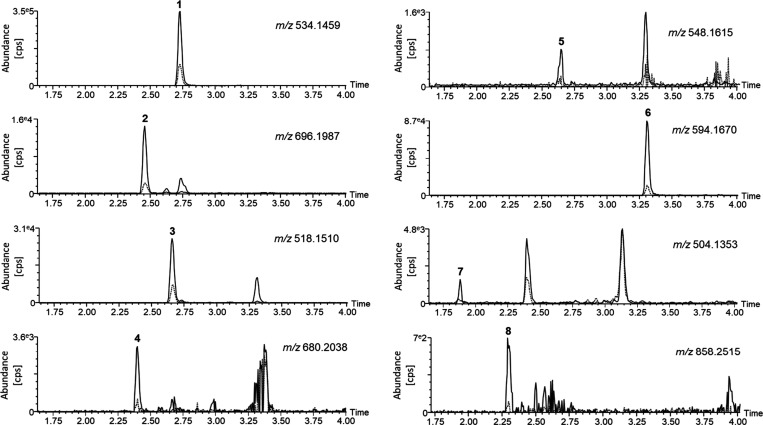
Extracted ion chromatograms of multihexose benzoxazinoids detected in control (dashed line) and drought-stressed (solid line) maize leaves. (1) Putative DIMBOA-dihexose (*m*/*z* 534.1459), (2) putative DIMBOA-trihexose (*m*/*z* 696.1987), (3) putative HMBOA-dihexose (*m*/*z* 518.1510), (4) putative HMBOA-trihexose (*m*/*z* 680.2038), (5) putative HM_2_BOA-dihexose (*m*/*z* 548.1615), (6) putative HDMBOA-dihexose (*m*/*z* 594.1670), (7) putative DIBOA-dihexose (*m*/*z* 504.1353), and (8) putative DIMBOA-tetrahexose (*m*/*z* 858.2515). cps: count per second. *m*/*z*: mass-to-charge ratio.

Although BXDs with multiple hexose units were previously reported,^4,10−16,21^ their exact structures remained unidentified, probably due to their low concentrations in plants. During our investigation on stress-induced BXDs in maize, we observed that drought induced higher amounts of putative di-, tri-, and tetrahexose BXDs in maize (Figures 1 and 2).

Extracted ion chromatograms revealed drought-increased peaks for DIMBOA-dihexose (*m*/*z* 534.1459, **1**), DIMBOA-trihexose (*m*/*z* 696.1987, **2**), HMBOA-dihexose (*m*/*z* 518.1510, **3**), HMBOA-trihexose (*m*/*z* 680.2038, **4**) HM_2_BOA-dihexose or HDMBOA-dihexose (*m*/*z* 548.1615 and 594.1670, **5** and **6**), DIBOA-dihexose (*m*/*z* 504.1353, **7**), and DIMBOA-tetrahexose (*m*/*z* 858.2515, **8**) (Figure 2). As high-resolution mass spectrometry was unable to provide the exact nature and linkage of those hexose units as well as to distinguish between isomeric aglucones such as HM_2_BOA and HDMBOA, we decided to purify these multihexose BXDs from large enough quantities of plant material to enable NMR analyses.

While certain dihexose BXDs such as DIMBOA-dihexose (**1**) or HDMBOA/HM_2_BOA-dihexose (**6**) were produced in fairly high concentrations as shown by their prominent peaks in the chromatogram, others were present in much lower amounts (e.g., HMBOA-trihexose (**4**), DIBOA-dihexose (**7**), Figures 1 and 2). Compound **8** was present in such low quantities that it could not be isolated from the plant material. To elucidate the structure of all detected di- and trihexose BXDs, we purified them from maize plants grown under drought for 10 days and fully characterized them by a combination of spectroscopic techniques.

### 3.2 Structural Elucidation of Multihexose Benzoxazinoids

**Figure 3:**
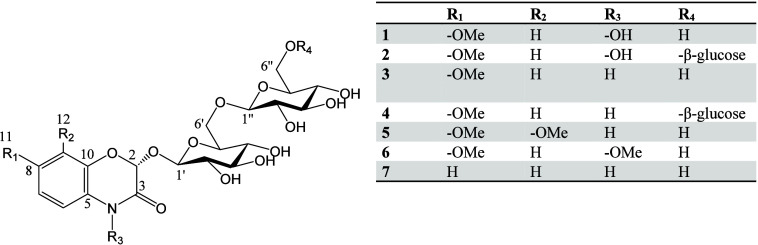
Structure of compounds **1** to **7**.

A methanol extract from maize roots was first fractionated by open column chromatography using a step gradient elution. The fraction (F9) containing dihexose and trihexose BXDs was eluted with a 1/1 mixture of ethyl acetate and methanol. This fraction was submitted to multiple steps of semipreparative fractionation to obtain compounds **1**–**7**. All of the purification steps were controlled by LC-MS and the purity of each isolated compound was finally checked by NMR spectroscopy. Compounds **1** to **7** were identified as multihexose BXDs (Figure 3).

In the following section, the structural elucidation of compounds **1** to **7**, based on NMR, HRMS and circular dichroism (CD) data, is briefly described. All ^1^H NMR chemical shifts are displayed in Table 1 and a detailed report of all obtained data is provided in Supporting Information SI2–SI8.

**Table 1:** ^1^H NMR (600 MHz, D_2_O) Data for Compounds **1**–**7**

**no.**		**1**	**2**	**3**	**4**	**5**	**6**	**7**
2		5.99	5.92	5.84	5.84	6.07	6.02	5.95
5		7.38	7.47	7.01	7.02	7.19	7.36	7.58
6		6.83	6.82	6.76	6.78	6.91	6.88	7.19
7								7.24
8		6.85	6.81	6.86	6.86		6.92	7.18
11		3.84	3.85	3.83	3.84	3.89	3.87	
12						3.92		
N-OMe							3.98	
1′		4.86	4.85	4.86	4.86	4.88	4.88	4.87
2′		3.25	3.26	3.25	3.27	3.25	3.25	3.25
3′		3.51	3.50	3.51	3.50	3.52	3.50	3.54
4′		3.49	3.49	3.50	3.49	3.51	3.49	3.50
5′		3.63	3.64	3.61	3.61	3.60	3.63	3.68
6′	a	3.92	3.94	3.91	3.96	3.93	3.93	3.90
	b	4.13	4.13	4.13	4.12	4.07	4.14	4.21
1″		4.40	4.37	4.41	4.35	4.39	4.41	4.48
2″		3.28	3.29	3.29	3.28	3.23	3.29	3.31
3″		3.44	3.48	3.45	3.43	3.42	3.46	3.47
4″		3.39	3.43	3.39	3.48	3.36	3.40	3.42
5″		3.27	3.30	3.27	3.32	3.23	3.28	3.34
6″	a	3.71	3.82	3.72	3.82	3.69	3.73	3.74
	b	3.87	4.14	3.88	4.15	3.84	3.89	3.92
1‴			4.49		4.49			
2‴			3.30		3.31			
3‴			3.49		3.49			
4‴			3.38		3.39			
5‴			3.43		3.43			
6‴	a		3.71		3.72			
	b		3.90		3.90			

The elemental composition of **1** was deduced to be C_21_H_29_NO_15_ based on the HRESIMS and NMR data (Table 1, Supporting Information SI2). The chemical shifts, multiplicity, and coupling constant of the aromatic protons were characteristic of a monosubstituted aromatic ring fused to 1,4-benzoxazine [(7.38 (d, *J* = 8.9 Hz), 6.85 (d, *J* = 2.6 Hz), 6.83 (dd, *J* = 8.9, 2.6 Hz)]. The proton H-11 of the methoxy group [δ_C_ 56.2 (CH_3_), δ_H_ 3.80] showed a long-range HMBC cross-peak with the carbon C-7. Thus, the aglycone was DIMBOA (2,4-dihydroxy-7-methoxy-1,4-benzoxazin-3-one). The remaining signals observed in the ^1^H and ^13^C NMR spectra exhibited resonance of two hexose units characterized by two anomeric methines [δ_C_ 102.5 (CH), δ_H_ 4.86 (d, 7.9 Hz)] and [δ_C_ 103.5 (CH), δ_H_ 4.40 (d, 7.8 Hz)], two methylenes [δ_C_ 68.9 (CH_2_), δ_H_ 3.92/4.13] and [δ_C_ 60.9 (CH_2_), δ_H_ 3.87/3.71] and two closely related series of 4 methines bonded with an oxygen. Long range HMBC spectrum displayed correlations from H-1′ (δ_H_ 4.86) to C-2 (δ_C_ 97.7) and from both H-6′a (δ_H_ 4.13) and H-6′b (δ_H_ 3.92) to C-1″ (δ_C_ 103.5), which established the linkage between the aglycone part and the sugar units (1′→2) and between the two sugar units (1″→6′). Both glucose units exhibited β conformations [δ_C_ 102.5 (CH), δ_H_ 4.86 (d, 7.9 Hz)] and [δ_C_ 103.5 (CH), δ_H_ 4.40 (d, 7.8 Hz)]. The coupling constants ^1^*J*_(C–H)_ of 163.7 and 161.8 Hz of both anomeric carbons C1′ and C1″ were further evidence of a β conformation. Selective 1D-TOCSY, 1D-NOESY together with 2D-band selective HSQC were employed to unambiguously assign chemical shifts and to determine the stereochemistry of the sugar units. Correlations between H2 (δ_H_ 5.99) and H-1′ (δ_H_ 4.86) confirmed the β linkage between the DIMBOA aglycone and the sugar unit. Additional NOESY enhancement observed from H-1′ to H-3′ and to H-5′ and in a similar way from H-1″ to H-3″ and to H-5″ indicated that they have a *syn* orientation. The (2R) configuration could be proven by means of the CD spectrum (Supporting Information SI2), showing a positive Cotton effect at 231 nm and a negative one at 315 nm in aqueous solution. This feature is in coincidence with that of the closely related (2R)-2-β-d-glucopyranosyloxy-7-methoxy-2*H*-1,4-benzoxazin-3(4H)-one.^22^ Compound **1** was thus identified as DIMBOA-β-gentiobiose (DIMBOA-2Glc) exhibiting a β-d-glucopyranosyl-(1″→6′)-β-d-glucopyranose sugar unit (Figure 3).

The elemental composition of **2** was deduced to be C_27_H_39_NO_20_ based on the HRESIMS and NMR data (Table 1, Supporting Information SI3). The aglycone part was identified as DIMBOA. Signals observed in the ^1^H and ^13^C NMR spectra of **2** exhibited an extra set of chemical shifts in the glucoside region of both spectra matching with the additional C_6_H_10_O_5_ moiety compared to **1**. Long range HMBC spectrum displaying correlations from H-6″a (δ_H_ 4.13) to C-1‴ (δ_C_ 103.7) established the linkage between the second and the third sugar units (1‴→6″). The third glucose unit exhibited an β conformation [δ_C_ 103.7 (CH), δ_H_ 4.49 (d, 8.0 Hz)]. The coupling constants ^1^*J*_(C–H)_ of anomeric carbons C 1′, C 1″ and C 1‴, 162.0, 161.3, and 159.3 Hz, respectively, are characteristic of a β conformation. The (2R) configuration could be proven by means of the CD spectrum, showing a positive Cotton effect at 231 nm and a negative one at 282 nm in aqueous solution. This feature is in accordance with that of the closely related DIMBOA-β-gentiobiose. Compound **2** was thus identified as DIMBOA-β-d-glucopyranosyl-(1″→6′)-β-d-glucopyranosyl-(1‴→6″)-d-β-glucopyranose (DIMBOA-3Glc, Figure 3).

The elemental composition of **3** was deduced to be C_21_H_29_NO_14_ based on the HRESIMS and NMR data (Table 1, Supporting Information SI4). Compound **1** possessed extra oxygen compared to **3**. NMR data of **1** and **3** were closely related. The alcohol function located on the nitrogen induced a gamma-steric effect for compound 1. This gamma-steric effect of the alcohol function located on the nitrogen induced a variation of the ^13^C chemical shift value of Δδ = −5.1 ppm on C-3 of 1 compared to 3 (Supporting Information SI2–SI4). NOESY correlations and the CD spectrum confirmed the structure of the dihexose unit as well as the (2R) configuration (Supporting Information SI4). Compound **3** was identified as HMBOA-β-gentiobiose (HMBOA-2Glc, Figure 3).

The elemental composition of **4** was deduced to be C_27_H_39_NO_19_ based on the HRESIMS and NMR data (Table 1, Supporting Information SI5). Signals observed in the ^1^H and ^13^C NMR spectra exhibited signals related to both the HMBOA aglycone and the trihexose unit previously described for **2**. CD spectrum confirmed the (2R) configuration. Compound **4** was identified as HMBOA-β-d-glucopyranosyl-(1″→6′)-β-d-glucopyranosyl-(1‴→6″)-d-β-glucopyranose (HMBOA-3Glc, Figure 3).

The elemental composition of **5** was deduced to be C_22_H_31_NO_15_ based on the HRESIMS and NMR data (Table 1, Supporting Information SI6). Signals observed in the ^1^H and ^13^C NMR spectra of **5** exhibited resonances for two methoxy groups [δ_C_ 56.2 (CH_3_), δ_H_ 3.89] and [δ_C_ 61.6 (CH_3_), δ_H_ 3.92]. Protons H-11 and H-12 located on each of the methoxy groups showed long-range HMBC cross-peaks, respectively, with the carbons C-8 and C-7. The aglycone unit was identified as HM_2_BOA. Chemical shifts of the two hexose units were closely related to both compounds **1** and **3** (Table 1). NOESY correlations and CD spectrum confirmed the structure of the dihexose unit as well as the (2R) configuration. Compound **5** was identified as HM_2_BOA-β-gentiobiose (HM_2_BOA-2Glc, Figure 3).

Compound **6** exhibited a prominent adduct [M+HCOO]^−^ at *m*/*z* 594.1670 and a minor [M-H]^−^ at *m*/*z* 548.1619 leading to the same elemental composition C_22_H_31_NO_15_ as **5** based on the HRESIMS and NMR data (Table 1, Supporting Information SI7). The proton NMR spectrum exhibited two singlet proton signals characteristic of methoxy groups [δ_C_ 56.3 (CH_3_), δ_H_ 3.87] and [δ_C_ 63.6 (CH_3_), δ_H_ 3.98]. Long-range HMBC showed cross-peaks between H-11 and C-7. Spectral data of the second methoxy group [δ_C_ 63.6 (CH_3_), δ_H_ 3.98] were characteristic of an N-OMe group. The aglycone unit was identified as HDMBOA. Chemical shifts of the two hexose units were closely related to compounds **1**, **3**, and **5** (Table 1). NOESY correlations and the CD spectrum confirmed the structure of the dihexose unit as well as the (2R) configuration. Compound **6** was identified as HDMBOA-β-gentiobiose (HDMBOA-2Glc, Figure 3).

The elemental composition of **7** was deduced to be C_20_H_27_NO_14_ based on the HRESIMS and NMR data (Table 1, Supporting Information SI8). Signals observed in the ^1^H spectrum were characteristic of an aromatic ring without any substituents fused to 1,4-benzoxazine [7.58 (dd, *J* = 7.5, 2.5 Hz), 7.24 (ddd, *J* = 7.9, 6.5, 2.5 Hz), 7.19 (dd, *J* = 7.5, 6.5 Hz), 7.18 (d, *J* = 7.9 Hz)]. Thus, the aglycone was DIBOA. Chemical shifts of the two hexose units were closely related to compounds **1**, **3, 5** and **6** (Table 1). Compound **7** was identified as DIBOA-β-gentiobiose (DIBOA-2Glc, Figure 3).

### 3.3 Drought Profoundly Modifies Benzoxazinoid Profiles in Leaf and Root Tissues

**Figure 4:**
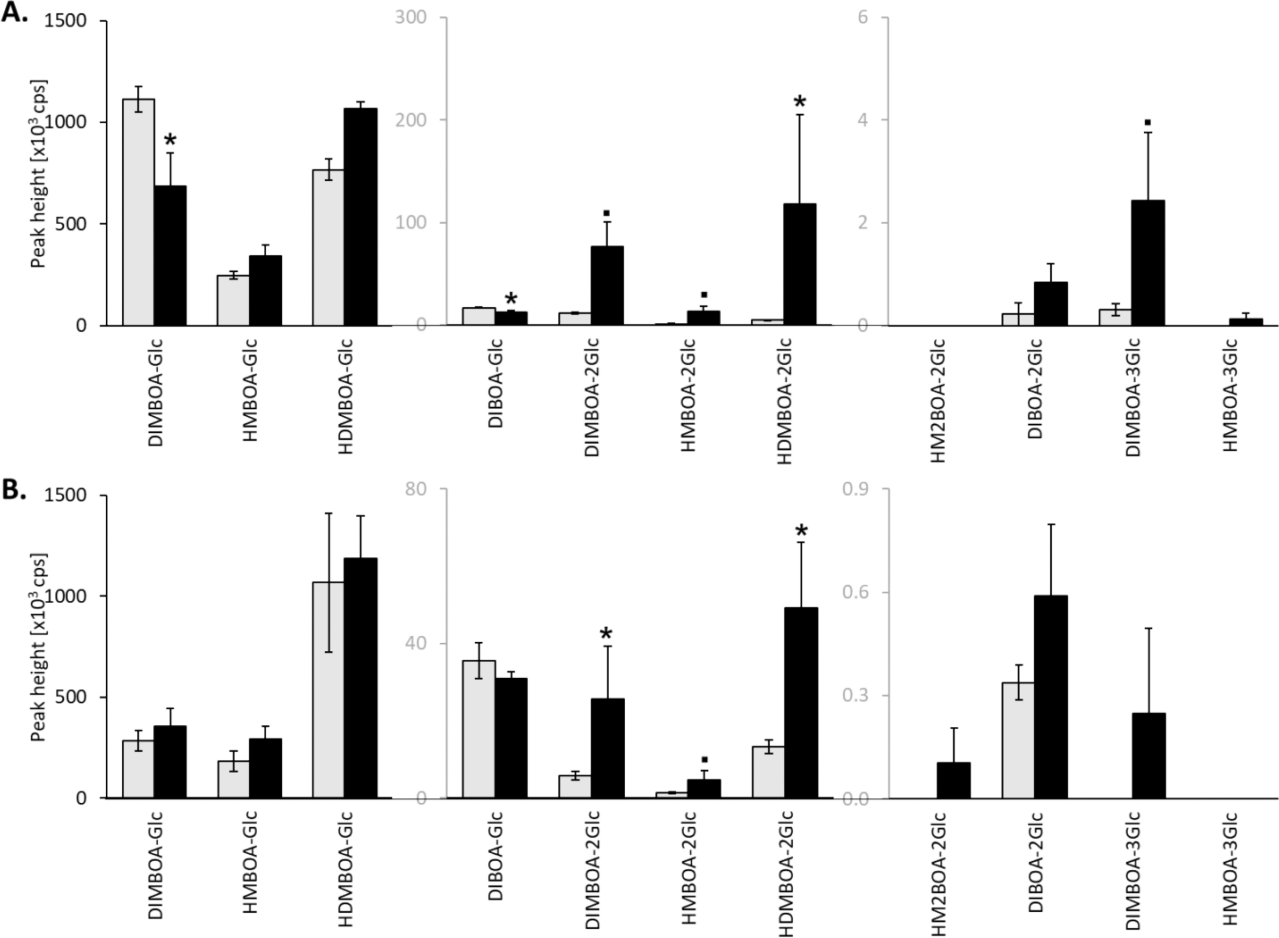
Drought induced di- and trihexose benzoxazinoids (BXDs) in maize roots and shoots. BXD peak heights (Mean ± sem) in B73 maize leaves (A) and roots (B) (*n* = 4–5 per treatment and tissue). Drought was established 4 days after sowing and all plants were harvested 6 days later. Gray bars: ambient conditions, black bars: drought conditions. cps: count per second. Student *t* tests and Mann–Whitney Rank Sum tests were conducted. Dots and stars indicate trends and significant differences respectively (.: *p* < 0.10, * *p* < 0.05).

Drought decreased DIMBOA-Glc and DIBOA-Glc levels but increased the levels of DIMBOA-2Glc, HMBOA-2Glc, DIMBOA-3Glc, and HDMBOA-2Glc in maize leaves. Drought further induced higher levels of DIMBOA-2Glc, HMBOA-2Glc, and HDMBOA-2Glc in roots (Figure 4). The production of di- and trihexose BXDs was previously observed in maize^13^ and was induced under stressful conditions.^15,16^

### 3.4 Induction of Dihexose- and Trihexose Benzoxazinoids Is Transient

**Figure 5:**
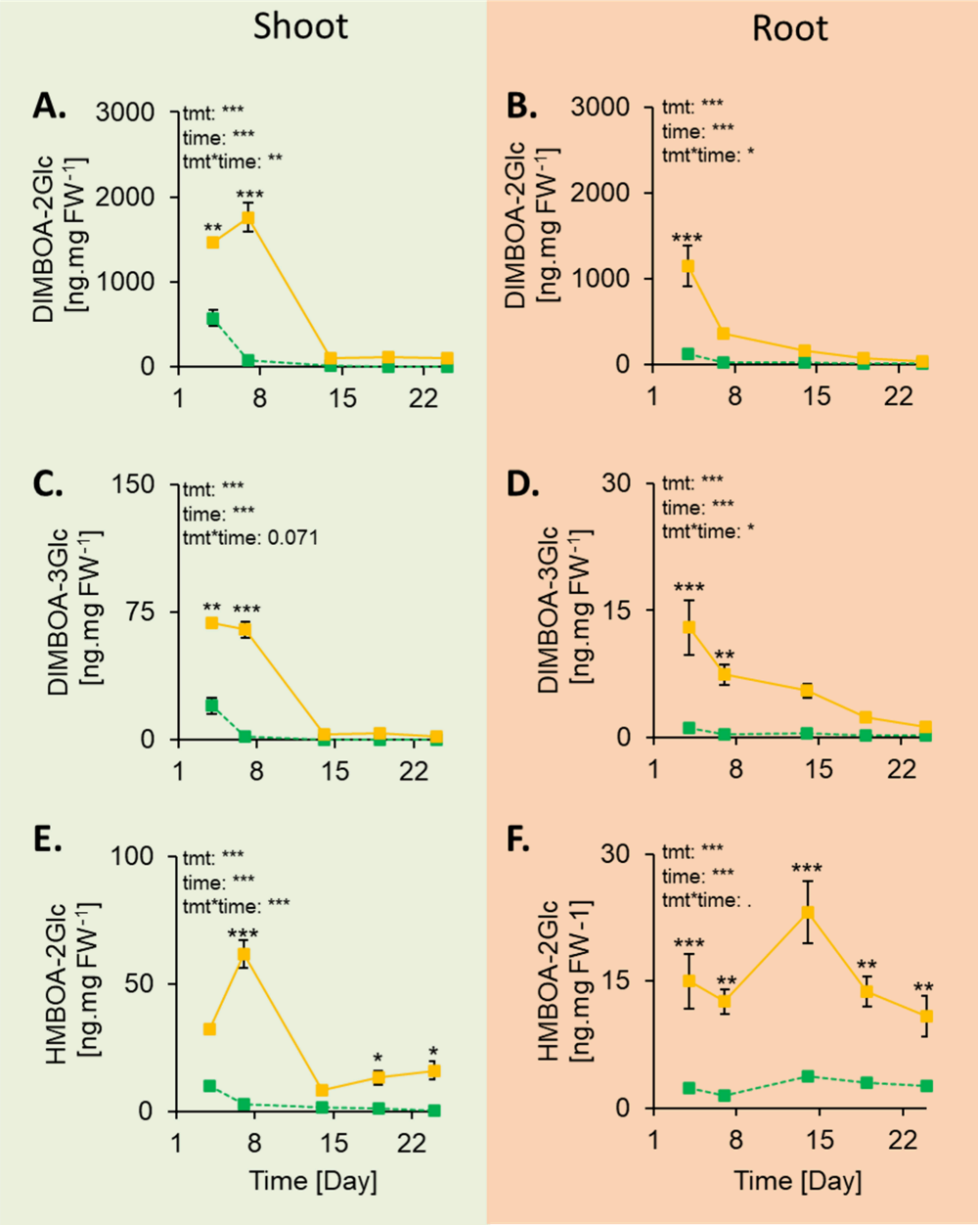
Drought-mediated induction of double and triple hexoses was transient. DIMBOA-2Glc concentrations in maize shoots (A) and roots (B) under control and drought conditions. DIMBOA-3Glc concentrations in maize shoots (C) and roots (D) under control and drought conditions. HMBOA-2Glc concentrations in maize shoots (E) and roots (F) under control and drought conditions (*n* = 8 per treatment and time point). Control (green lines): 23% soil moisture (v/v); Drought (yellow lines): 16.6% soil moisture (v/v). tmt: treatment (control or drought). FW: fresh weight. Mean ± SEM are shown. Two-way ANOVAs on ranks were conducted, followed by posthoc Holm–Sidak tests when relevant. Stars indicate significant differences within time points (.: *p* < 0.10; *: *p* < 0.05; **: *p* < 0.01; ***: *p* < 0.001).

Under ambient conditions, the oligosaccharides DIMBOA-2Glc, and DIMBOA-3Glc could only be detected in four-day-old seedlings, as their levels dropped under limit of detection afterward (Figure 5A-D). However, low levels of HMBOA-2Glc could be detected in roots over 24 days of growth (Figure 5E,F).

Under drought conditions, maize seedlings transiently produced higher concentrations of DIMBOA-2Glc, DIMBOA-3Glc, and HMBOA-2Glc in both shoot and root tissues (Figure 5). The production of the di- and trihexose BXDs was the highest in roots of 4-day-old seedlings and in shoots of 7-day-old seedlings (Figure 5). Whether these BXDs are produced in roots and subsequently transported to shoots remains to be investigated.

### 3.5 Drought-Induced Multihexose Benzoxazinoid Production Is Common in Maize

**Figure 6:**
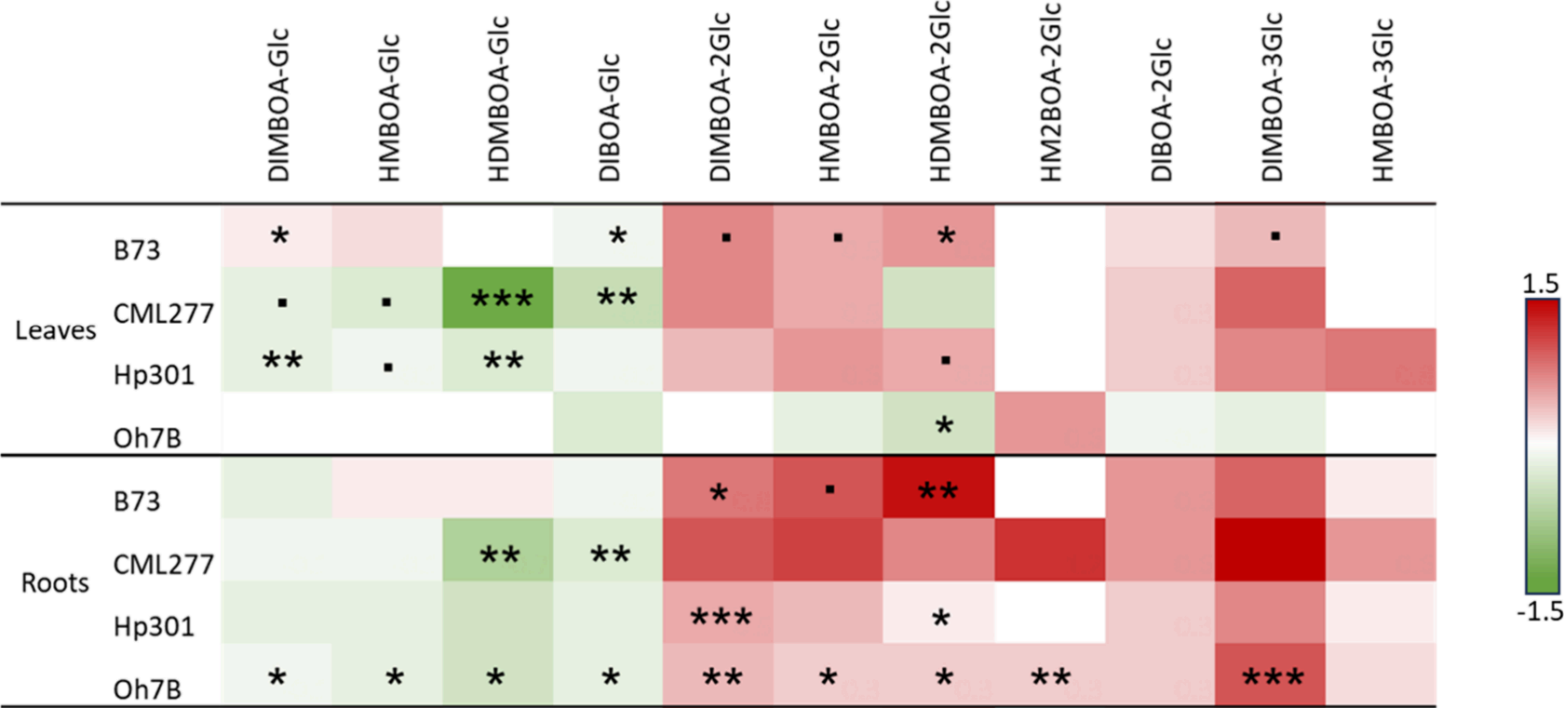
Multihexose benzoxazinoids were induced by drought in several maize varieties. Heatmap of benzoxazinoid (BXD) levels in drought-stressed plants (leaves and roots) compared to control plants in B73, CML277, Hp301, and Oh7B maize varieties (*n* = 2–5 per treatment and variety). The log fold (log10) changes between drought-stressed and control plants are shown. Green indicates a decrease in BXD levels upon drought. Red indicates an increase in BXD levels upon drought. Student *t* tests and Mann–Whitney Sum Rank tests were conducted. Dots and stars indicate trends and significant differences respectively (.: *p* < 0.10, *: *p* < 0.05, **: *p* < 0.01, ***: *p* < 0.001).

The drought-mediated induction of multihexose BXDs was found in leaves and roots of the four maize varieties, B73, CML277, Hp301, and Oh7B (Figure 6). Interestingly, the increase in multihexose BXDs was accompanied by a decrease in monoglucosylated BXDs, suggesting their use as precursors for multihexose BXDs (Figure 6). Some line-specific patterns were further observed, suggesting that genetic variability could be used to identify the biosynthesis pathways and glucosyl transferases involved in the production of multihexose BXDs.

### 3.6 Toward the Function(s) of Multihexose Benzoxazinoids

Overall, this study reports a method to produce maize extracts enriched in multihexose BXDs and provides the first characterization of the exact hexose nature and configuration. Glycosylation of a small organic compound can alter its solubility, stability, bioavailability, and bioactivity and modulate its storage, transport, and/or interactions with other organisms. Plant multihexose secondary metabolites have been previously reported in wheat, rye, tomato, daisy flowers, Madagascar periwinkle flowers, and Japanese morning glory.^23−26^ They include phenolic compounds such as eugenol triglycosides^26^ and flavonoids, such as quercetin 3-*O*-gentiobiosides, gentiotriosides, and gentiotetrosides,^23^ anthocyanidin 3-*O*-rutinosides,^24,25^ or 3-*O*-2″-*O*-β-glucuronosyl-6″-*O*-malonylglucoside.^24^ Evidence suggests that the conversion of glycoside plant specialized metabolites into multihexoses can modulate plant volatile emissions^26^ and flower coloration.^24^

The role of multihexose BXDs still remains to be elucidated. Several, nonexclusive, hypotheses can be formulated. First, multihexose BXDs may act as osmoprotectants through maintaining cell turgor pressure and osmotic balance. Second, these BXDs may form, through the sugar moieties, hydrogen bonds with water molecules and reduce the water loss associated with transpiration. Third, the multihexose compounds may act as stronger antioxidants than their precursors and prevent cellular damage associated with reactive oxygen species (ROS). Fourth, multihexoses may be used to reallocate and store sugars as an energy source to tolerate drought and decreased photosynthesis. Fifth, because BXDs modulate the plant interactions with pathogens and herbivores, multihexose BXDs may be involved in protecting the plant from biotic stress under challenging abiotic conditions. Alternatively, the sugar-rich compounds may be exuded in the rhizosphere and promote beneficial microorganisms. While our understanding of the biosynthesis and ecological functions of multihexose plant specialized metabolites is still in its infancy, they represent a fascinating aspect of phytochemical diversity, calling for future research.

## Supporting Information Available

(SI1) Preparative chromatography; (SI2) structure elucidation of DIMBOA-2Glc (**1**); (SI3) structure elucidation of DIMBOA-3Glc (**2**); (SI4) structure elucidation of HMBOA-2Glc (**3**); (SI5) structure elucidation of HMBOA-3Glc (**4**); (SI6) structure elucidation of HM_2_BOA-2Glc (**5**); (SI7) structure elucidation of HDMBOA-2Glc (**6**); (SI8) structure elucidation of DIBOA-2Glc (**7**) (PDF)

## Abbreviations Used

ACN: acetonitrile
BPI: base peak intensity chromatogram
BXD: benzoxazinoid
CHCl_3_: chloroform
CH: Switzerland
CLIP-HSQC: coupled heteronuclear single quantum coherence clean inphase multiplets
COSY: correlation spectroscopy (NMR spectroscopy)
DE: Germany
DIBOA: 2,4-dihydroxy-1,4-benzoxazin-3-one
DIMBOA: 2,4-dihydroxy-7-methoxy-1,4-benzoxazin-3-one
EtOH: ethanol
Glc: glucose
Hex: hexose
HBOA: 2-hydroxy-1,4-benzoxazin-3-one
HDMBOA: 2-hydroxy-4,7-dimethoxy-1,4-benzoxazin-3-one
HMBC: heteronuclear multiple bond correlation (NMR spectroscopy)
HMBOA: 2-hydroxy-4-methoxy-1,4-benzoxazin-3-one
HM_2_BOA: 2-hydroxy-4,7-dimethoxy-1,4-benzoxazin-3-one
HPLC: high-performance liquid chromatography
HTP: hydrothermal processing
HRESIMS: high-resolution electrospray mass spectrometry
HSQC: heteronuclear single quantum coherence (NMR spectroscopy)
IPCC: Intergovernmental Panel on Climate Change
LC: liquid chromatography
MeOH: methanol
MS: mass spectrometry
*m*/*z*: mass-to-charge ratio
NMR: nuclear magnetic resonance
NOESY: nuclear Overhauser effect spectroscopy (NMR spectroscopy)
ppm: parts per million
QTOF: quadrupole time-of-flight (mass spectrometry)
RCP: representative concentration pathways (climate modeling)
TOCSY: total correlation spectroscopy (NMR spectroscopy)
UPLC: ultrahigh-performance liquid chromatography
USA: United States of America

## References

[ref1] Robert C. A. M.; Mateo P. The Chemical Ecology of Benzoxazinoids. Chimia (Aarau) 2022, 76 (11), 928 10.2533/chimia.2022.928. 38069788

[ref2] Gross J. J.; Mateo P.; Ramhold D.; Kramer E.; Erb M.; Robert C. A. M. Turnover of Benzoxazinoids during the Aerobic Deterioration of Maize Silage (Zea Mays). J. Agric. Food Chem. 2023, 71 (5), 2370–2376. 10.1021/acs.jafc.2c06699. 36692976 PMC9912334

[ref3] Pedersen H. A.; Laursen B.; Mortensen A.; Fomsgaard I. S. Bread from Common Cereal Cultivars Contains an Important Array of Neglected Bioactive Benzoxazinoids. Food Chem. 2011, 127 (4), 1814–1820. 10.1016/j.foodchem.2011.02.070.

[ref4] Steffensen S. K.; Adhikari K. B.; Laursen B. B.; Jensen C.; Gregersen P. L.; Bhattarai B.; Maraís L. M.; Schnorr H.; Jensen B. M.; Poulsen L. K.; Nielsen C. H.; Borre M.; Borre M.; Høyer S.; Fomsgaard I. S. Bioactive Small Molecules in Commercially Available Cereal Food: Benzoxazinoids. J. Food Compos. Anal. 2017, 64, 213–222. 10.1016/j.jfca.2017.10.001.

[ref5] Gross J. J.; Mateo P.; Schlaeppi K.; Wyss U.; Kramer E.; Ramhold D.; Erb M.; Robert C. A. M. Short Communication: Metabolization of Benzoxazinoids during Silage Fermentation of Maize and Their Effects on Silage Quality. Anim Feed Sci. Technol. 2023, 304, 115748 10.1016/j.anifeedsci.2023.115748.

[ref6] Adhikari K. B.; Tanwir F.; Gregersen P. L.; Steffensen S. K.; Jensen B. M.; Poulsen L. K.; Nielsen C. H.; Høyer S.; Borre M.; Fomsgaard I. S. Benzoxazinoids: Cereal Phytochemicals with Putative Therapeutic and Health-Protecting Properties. Mol. Nutr. Food Res. 2015, 59 (7), 1324–1338. 10.1002/mnfr.201400717. 25600612

[ref7] Matos P.; Figueirinha A.; Paranhos A.; Nunes F.; Cruz P.; Geraldes C. F. G. C.; Cruz M. T.; Batista M. T. Bioactivity of Acanthus Mollis – Contribution of Benzoxazinoids and Phenylpropanoids. J. Ethnopharmacol 2018, 227, 198–205. 10.1016/j.jep.2018.09.013. 30201231

[ref8] Bhattarai B.; Steffensen S. K.; Staerk D.; Laursen B. B.; Fomsgaard I. S. Data-Dependent Acquisition-Mass Spectrometry Guided Isolation of New Benzoxazinoids from the Roots of Acanthus Mollis L. Int. J. Mass Spectrom. 2022, 474, 116815 10.1016/j.ijms.2022.116815.

[ref9] Nordin E.; Steffensen S. K.; Laursen B. B.; Andersson S.-O.; Johansson J.-E.; Åman P.; Hallmans G.; Borre M.; Stærk D.; Hanhineva K.; Fomsgaard I. S.; Landberg R. An Inverse Association between Plasma Benzoxazinoid Metabolites and PSA after Rye Intake in Men with Prostate Cancer Revealed with a New Method. Sci. Rep 2022, 12 (1), 5260 10.1038/s41598-022-08856-z. 35347164 PMC8960836

[ref10] Pihlava J.-M.; Kurtelius T. Determination of Benzoxazinoids in Wheat and Rye Beers by HPLC-DAD and UPLC-QTOF MS. Food Chem. 2016, 204, 400–408. 10.1016/j.foodchem.2016.02.148. 26988518

[ref11] Bhattarai B.; Steffensen S. K.; Gregersen P. L.; Kristensen H. L.; Fomsgaard I. S. Stepwise Mass Spectrometry-Based Approach for Confirming the Presence of Benzoxazinoids in Herbs and Vegetables. Phytochemical Analysis 2021, 32 (3), 283–297. 10.1002/pca.2973. 32688439

[ref12] Dihm K.; Vendelbo Lind M.; Sundén H.; Ross A.; Savolainen O. Quantification of Benzoxazinoids and Their Metabolites in Nordic Breads. Food Chem. 2017, 235, 7–13. 10.1016/j.foodchem.2017.05.007. 28554649

[ref13] Fomsgaard I. S.; Mortensen A. G.; Holm P. B.; Gregersen P. L. Use of Benzoxazinoids-Containing Cereal Grain Products for Health-Improving Purposes. Patent application. EP 2 265 133 A12009.

[ref14] Hanhineva K.; Rogachev I.; Aura A.-M.; Aharoni A.; Poutanen K.; Mykkänen H. Qualitative Characterization of Benzoxazinoid Derivatives in Whole Grain Rye and Wheat by LC-MS Metabolite Profiling. J. Agric. Food Chem. 2011, 59 (3), 921–927. 10.1021/jf103612u. 21214244

[ref15] Pedersen H. A.; Heinrichson K.; Fomsgaard I. S. Alterations of the Benzoxazinoid Profiles of Uninjured Maize Seedlings during Freezing, Storage, and Lyophilization. J. Agric. Food Chem. 2017, 65 (20), 4103–4110. 10.1021/acs.jafc.7b01158. 28457134

[ref16] Batyrshina Z. S.; Shavit R.; Yaakov B.; Bocobza S.; Tzin V. The Transcription Factor TaMYB31 Regulates the Benzoxazinoid Biosynthetic Pathway in Wheat. J. Exp Bot 2022, 73 (16), 5634–5649. 10.1093/jxb/erac204. 35554544 PMC9467655

[ref17] van Doan C.; Pfander M.; Guyer A. S.; Zhang X.; Maurer C.; Robert C. A. M. Natural Enemies of Herbivores Maintain Their Biological Control Potential under Short-Term Exposure to Future CO2, Temperature, and Precipitation Patterns. Ecol Evol 2021, 11 (9), 4182–4192. 10.1002/ece3.7314. 33976802 PMC8093683

[ref18] Guyer A.; van Doan C.; Maurer C.; Machado R. A. R.; Mateo P.; Steinauer K.; Kesner L.; Hoch G.; Kahmen A.; Erb M.; Robert C. A. M. Climate Change Modulates Multitrophic Interactions Between Maize, A Root Herbivore, and Its Enemies. J. Chem. Ecol 2021, 47 (10), 889–906. 10.1007/s10886-021-01303-9. 34415498 PMC8613123

[ref19] IPCC. Climate Change 2014: Synthesis Report. Contribution of Working Groups I, II and III to the Fifth Assessment Report of the Intergovernmental Panel on Climate Change; IPCC, 2014.

[ref20] Robert C. A. M.; Zhang X.; Machado R. A. R.; Schirmer S.; Lori M.; Mateo P.; Erb M.; Gershenzon J. Sequestration and Activation of Plant Toxins Protect the Western Corn Rootworm from Enemies at Multiple Trophic Levels. Elife 2017, 6, e29307 10.7554/eLife.29307. 29171835 PMC5701792

[ref21] Tanwir F.; Fredholm M.; Gregersen P. L.; Fomsgaard I. S. Comparison of the Levels of Bioactive Benzoxazinoids in Different Wheat and Rye Fractions and the Transformation of These Compounds in Homemade Foods. Food Chem. 2013, 141 (1), 444–450. 10.1016/j.foodchem.2013.02.109. 23768378

[ref22] Nagao T.; Otsuka H.; Kohda H.; Sato T.; Yamasaki K. Benzoxazinones from Coix Lachryma-Jobi Var. Ma-Yuen. Phytochemistry 1985, 24 (12), 2959–2962. 10.1016/0031-9422(85)80035-2.

[ref23] Masada S.; Terasaka K.; Oguchi Y.; Okazaki S.; Mizushima T.; Mizukami H. Functional and Structural Characterization of a Flavonoid Glucoside 1,6-Glucosyltransferase from Catharanthus Roseus. Plant Cell Physiol 2009, 50 (8), 1401–1415. 10.1093/pcp/pcp088. 19561332

[ref24] Sawada S.; Suzuki H.; Ichimaida F.; Yamaguchi M.; Iwashita T.; Fukui Y.; Hemmi H.; Nishino T.; Nakayama T. UDP-Glucuronic Acid:Anthocyanin Glucuronosyltransferase from Red Daisy (Bellis Perennis) Flowers. Enzymology and Phylogenetics of a Novel Glucuronosyltransferase Involved in Flower Pigment Biosynthesis. J. Biol. Chem. 2005, 280 (2), 899–906. 10.1074/jbc.M410537200. 15509561

[ref25] Morita Y.; Hoshino A.; Kikuchi Y.; Okuhara H.; Ono E.; Tanaka Y.; Fukui Y.; Saito N.; Nitasaka E.; Noguchi H.; Iida S. Japanese Morning Glory Dusky Mutants Displaying Reddish-Brown or Purplish-Gray Flowers Are Deficient in a Novel Glycosylation Enzyme for Anthocyanin Biosynthesis, UDP-Glucose:Anthocyanidin 3-O-Glucoside-2″-O-Glucosyltransferase, Due to 4-Bp Insertions in the Gene. Plant Journal 2005, 42 (3), 353–363. 10.1111/j.1365-313X.2005.02383.x. 15842621

[ref26] Tikunov Y. M.; Molthoff J.; de Vos R. C. H.; Beekwilder J.; van Houwelingen A.; van der Hooft J. J. J.; Nijenhuis-de Vries M.; Labrie C. W.; Verkerke W.; van de Geest H.; Viquez Zamora M.; Presa S.; Rambla J. L.; Granell A.; Hall R. D.; Bovy A. G. NON-SMOKY GLYCOSYLTRANSFERASE1 Prevents the Release of Smoky Aroma from Tomato Fruit. Plant Cell 2013, 25 (8), 3067–3078. 10.1105/tpc.113.114231. 23956261 PMC3784599

