# Supplementary material for: Isolation and Structure Determination of Drought-Induced Multihexose Benzoxazinoids from Maize (Zea mays)
Source: J Agric Food Chem. 2024 Feb 9;72(7):3427–35. doi: 10.1021/acs.jafc.3c09141 (PMC10885146; doi:10.1021/acs.jafc.3c09141)

Isolation and structure determination of drought-induced multihexose benzoxazinoids from maize (*Zea mays*)

Supplementary Information SI1:
Preparative Chromatography.

Sylvain Sutour[1,*], Cong Van Doan[2,3,4], Pierre Mateo[2], Tobias Züst[5], Ella Raymonde Hartmann[2], Gaétan Glauser[1], Christelle Aurélie Maud Robert[2,3,*]

[1] Neuchâtel Platform of Analytical Chemistry, University of Neuchâtel, 2000 Neuchâtel, Switzerland

[2] Institute of Plant Sciences, University of Bern, 3013 Bern, Switzerland

[3] Oeschger Centre for Climate Change Research (OCCR), University of Bern, 3012 Bern, Switzerland

[4] Plant Physiology Unit, The Department of Life Sciences and Systems Biology of the University of Turin, Via Accademia Albertina 13, 10123 Torino, Italy

[5] Department of Systematic and Evolutionary Botany, University of Zürich, 8008 Zürich, Switzerland

For correspondence:Sutour Sylvain - sylvain.sutour@unine.ch; Christelle A. M. Robert - christelle.robert@unibe.ch

Semi-preparative HPLC separation of compounds **1-7** was first performed on an Xterra prep MS C18 OBD column (5 μm, 19×150 mm, Waters, USA) and yielded 90 fractions (F9.1-9.90). The elution conditions were as follows: solvent A, H_2O + 0.05% formic acid; solvent B, MeOH + 0.05% formic acid; flow rate, 8.0 mL/min; 0-60 min, linear gradient from 0 to 27.2% B; 60-70 min, linear gradient to 100% B; 70-80 min, 100% B. The elution was monitored at 216 nm and 260 nm. Fractions were collected every minute. F9.59 (3.9 mg) containing **7** was further purified by semi-preparative HPLC equipped with an Xbridge Prep Amide (5 μm, 10×250 mm, Waters, USA) column. The elution conditions were as follows: solvent A, H_2O + 0.05% formic acid; solvent B, MeOH + 0.05% formic acid; flow rate, 8.0 mL/min; 0-3 min at 80%, 3-38 min, linear gradient from 80 to 60% B, 30-40 min, linear gradient from 60 to 50% B. F9.59.9 contained pure **7** (0.6 mg). F9.60 contained pure **1** (2.6 mg) and did not require further purification. F9.61 (7.3 mg) and F9.62 (5.4 mg) containing a mixture of **2** and **4** were combined and further purified by semi-preparative HPLC equipped with Xterra prep MS C18 OBD (5 μm, 19×150 mm, Waters, USA) column. The elution conditions were as follows: solvent A, H_2O; solvent B, MeOH + 0.05% formic acid; flow rate, 8.0 mL/min; 0-3.5 min at 6%, 3.5-35 min, linear gradient from 6 to 19% B, 35-38 min, linear gradient from 19 to 100% B. F9.61.22 (0.8 mg) and F9.62.22 (0.6 mg) containing **2** were combined; F9.61.21 (2.4 mg) and F9.62.21 (0.9 mg) containing **4** were also combined. Both resulting fractions were further purified by semi-preparative HPLC equipped with Xselect CSH prep C18 OBD (5 μm, 19×250 mm, Waters, USA) column. The elution conditions were as follows: solvent A, H_2O; solvent B, MeOH + 0.05% formic acid; flow rate, 8.0 mL/min; 0-3.5 min at 9%, 3.5-35 min, linear gradient from 9 to 20% B, 35-38 min, linear gradient from 20 to 100% B. F9.61/62.21.21 contained pure **4** (0.6 mg). F9.61/62.22.23 contained pure **2** (0.8 mg). F9.65 contained pure **5** (1.5 mg). F9.68 (4.2 mg) containing **3** was further purified by semi-preparative HPLC equipped with an XBridge Prep Amide (5 μm, 10×250 mm, Waters, USA) column. The elution conditions were as follows: solvent A, H_2O + 0.05% formic acid; solvent B, MeOH + 0.05% formic acid; flow rate, 8.0 mL/min; 0-3 min at 80%, 3-38 min linear gradient from 80 to 50% B. F9.68.8 to F9.68.10 were combined and contained pure **3** (1.9 mg).

F9.80 (6.7 mg) containing **6** was further purified by semi-preparative HPLC equipped with an Xterra prep MS C18 OBD (5 μm, 19×150 mm, Waters, USA) column. The elution condition was as follows: solvent A, H_2O + 0.05% formic acid; solvent B, MeOH + 0.05% formic acid; flow rate, 8.0 mL/min; 0-3.5 min at 12% B, 3.5-37 min, linear gradient from 12 to 22% B; 37-38 min, linear gradient to 100% B; 38-45 min, 100% B. F9.80.19 to F9.80.20 were combined and contained pure **6** (1.7 mg).

Isolation and structure determination of drought-induced multihexose benzoxazinoids from maize (*Zea mays*)

Supplementary Information SI2:
Structure elucidation of DIMBOA-2Glc (1).

Sylvain Sutour[1,*], Cong Van Doan[2,3,4], Pierre Mateo[2], Tobias Züst[5], Ella Raymonde Hartmann[2], Gaétan Glauser[1], Christelle Aurélie Maud Robert[2,3,*]

[1] Neuchâtel Platform of Analytical Chemistry, University of Neuchâtel, 2000 Neuchâtel, Switzerland

[2] Institute of Plant Sciences, University of Bern, 3013 Bern, Switzerland

[3] Oeschger Centre for Climate Change Research (OCCR), University of Bern, 3012 Bern, Switzerland

[4] Plant Physiology Unit, The Department of Life Sciences and Systems Biology of the University of Turin, Via Accademia Albertina 13, 10123 Torino, Italy

[5] Department of Systematic and Evolutionary Botany, University of Zürich, 8008 Zürich, Switzerland

For correspondence:Sutour Sylvain - sylvain.sutour@unine.ch; Christelle A. M. Robert - christelle.robert@unibe.ch

Chemical data for **1**: violet needles; ECD (c 1.5 mg/mL, H2O) λmax (Δε) 231 (66.7), 315 nm (−11.3); ^{1}H NMR (D_2O, 600 MHz) δ 7.38 (1H, d, J = 8.9 Hz, H-5), 6.85 (1H, d, J = 2.6 Hz, H-8), 6.83 (1H, dd, J = 8.9, 2.6 Hz, H-6), 5.99 (1H, s, H-2), 4.86 (1H, d, J = 7.9 Hz, H-1'), 4.40 (1H, d, J = 7.9 Hz, H-1''), 4.13 (1H, dd, J = 11.0, 2.0 Hz, H-6'b), 3.92 (1H, dd, J = 11.0, 5.6 Hz, H-6'a), 3.87 (1H, dd, J = 12.2, 2.2 Hz, H-6''b), 3.84 (3H, s, OMe, H-11), 3.71 (1H, dd, J = 12.2, 5.6 Hz, H-6''a), 3.63 (1H, ddd, J = 7.6, 5.6, 2.0 Hz, H-5'), 3.51 (1H, m, H-3'), 3.49 (1H, m, H-4'), 3.44 (1H, dd, J = 9.7, 9.3 Hz, H-3''), 3.39 (1H, t, J = 9.7 Hz, H-4''), 3.28 (1H, dd, J = 9.3, 8.0 Hz, H-2''), 3.27 (1H, ddd, J = 9.7, 5.6, 2.2 Hz, H- 5''), 3.25 (1H, dd, J = 9.2, 7.9 Hz, H-2'); ^{13}C NMR (D2O, 151 MHz) δ 157.2 (C, C-7), 156.7 (C, C-3), 141.5 (C, C-9), 121.4 (C, C-10), 114.9 (CH, C-5), 109.4 (CH, C-6), 104.3 (CH, C-8), 103.5 (CH, C-1''), 102.5 (CH, C-1'), 97.7 (CH, C-2), 76.1 (CH, C-3''), 75.9 (CH, C-5''), 75.7 (CH, C-5'), 75.5 (CH, C-3'), 73.4 (CH, C-2''), 73.1 (CH, C-2'), 69.8 (CH, C-4''), 69.0 (CH, C-4'), 68.9 (CH2, C-6'), 60.9 (CH2, C-6''), 56.2 (CH3, C-11). HRESIMS m/z 534.1461 (calcd for C21H28NO15, 534.1459).

^{1}H NMR Spectrum of **1** (DMSO-*d6*, 600 MHz)

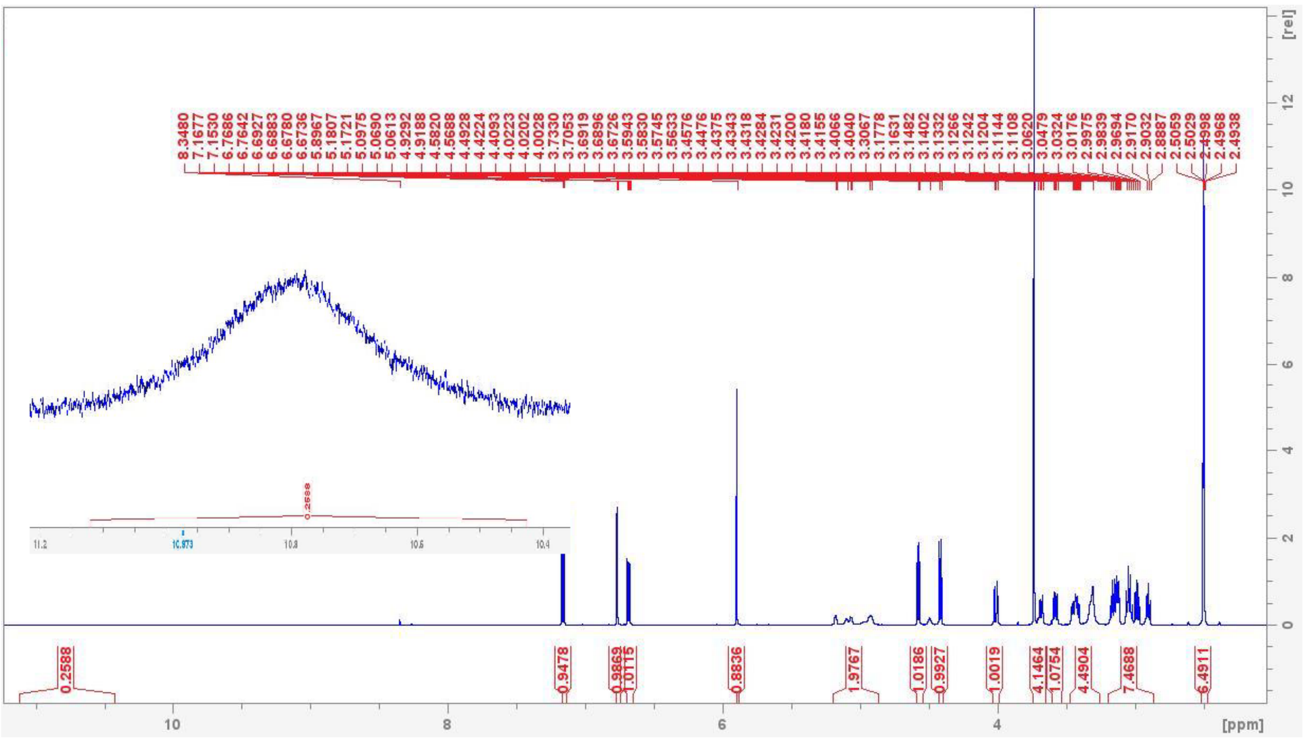

^{1}H NMR Spectrum of **1** (D_2O, 600 MHz)

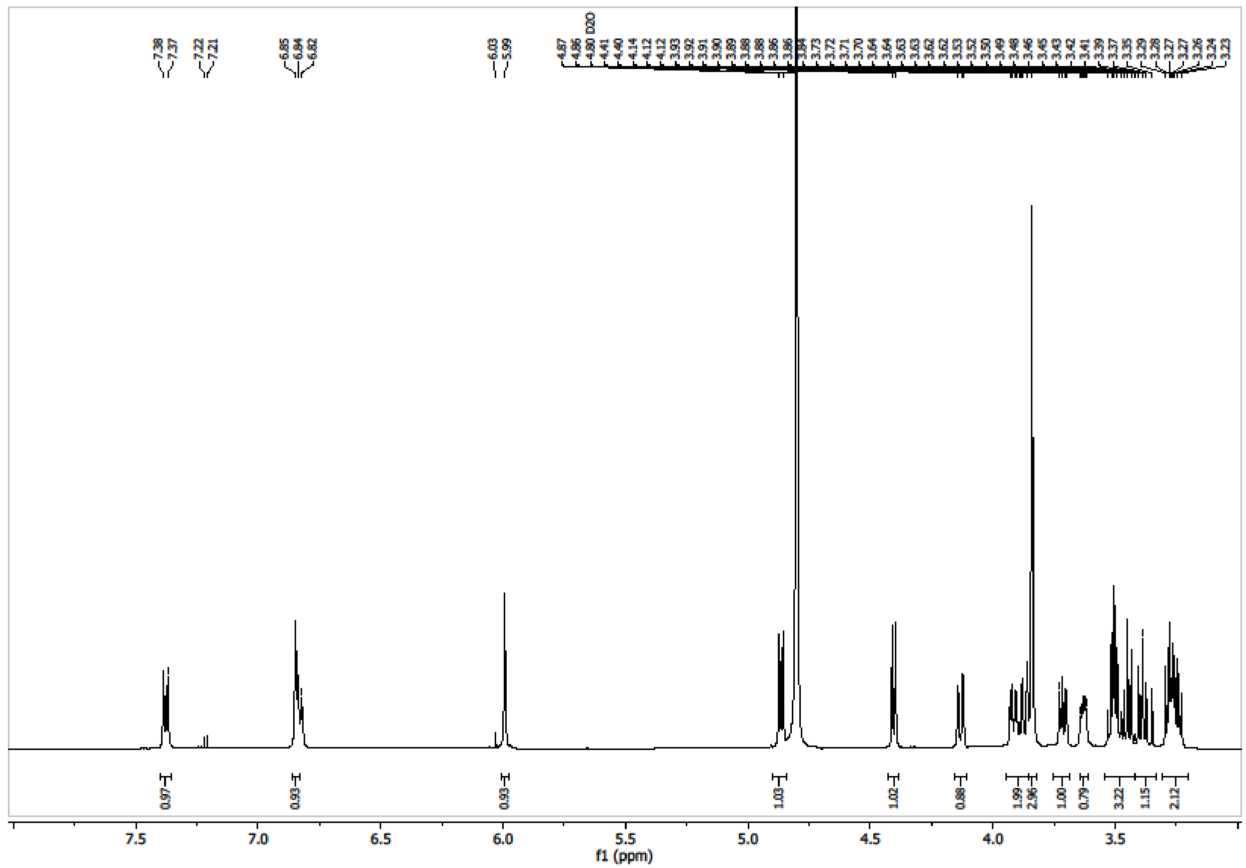

^{13}C NMR Spectrum of **1** (D_2O, 151 MHz)

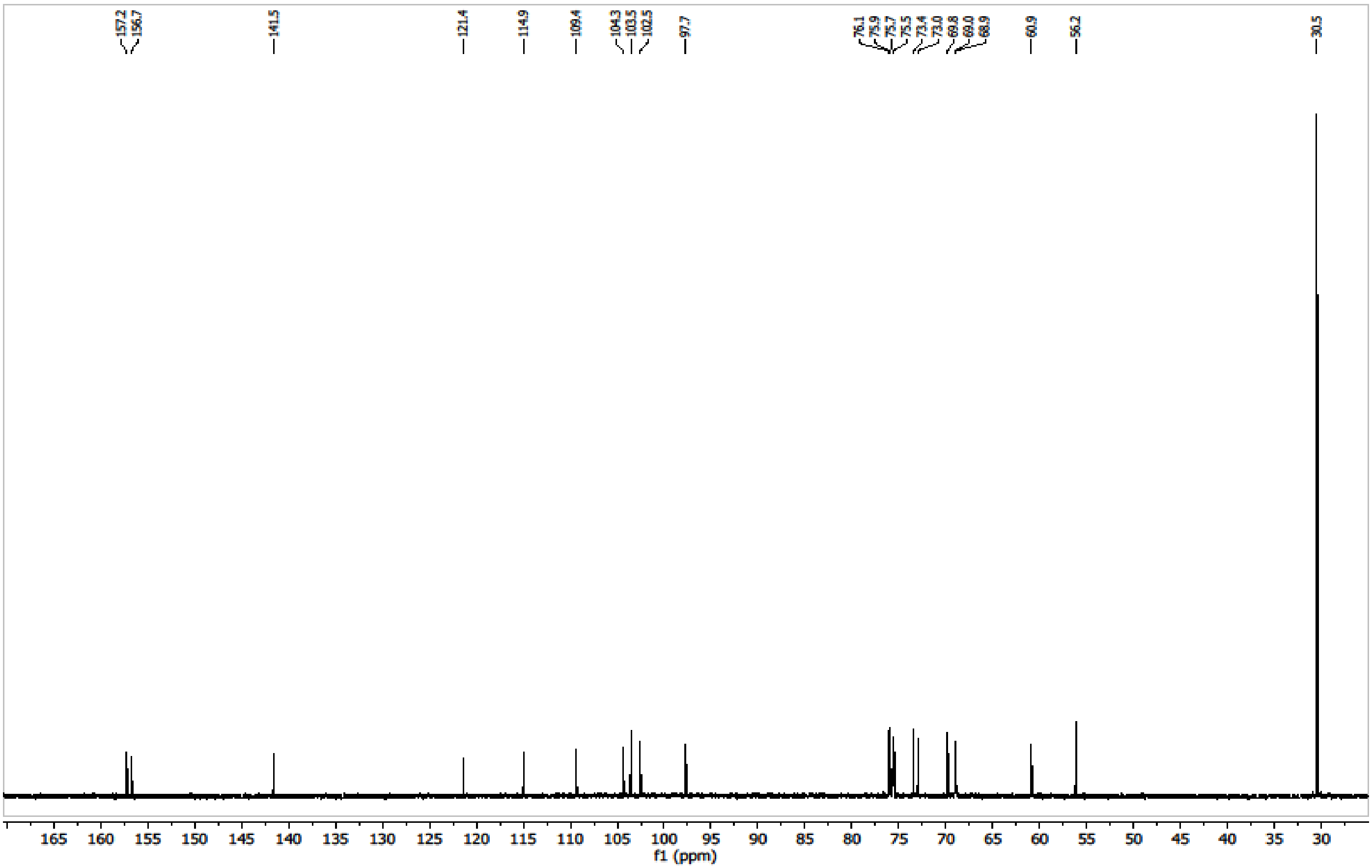

HSQC Spectrum of **1**

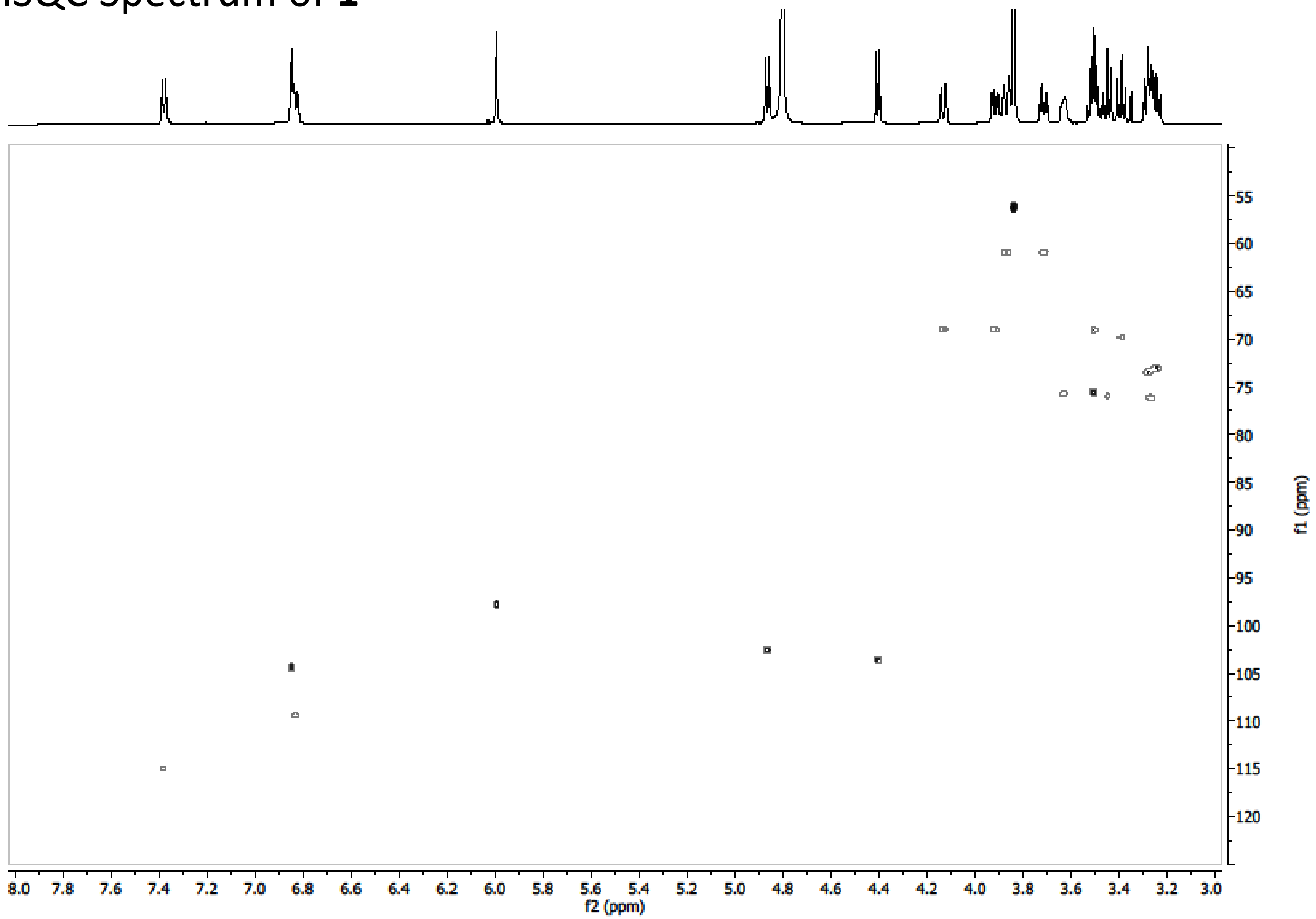

COSY Spectrum of **1**

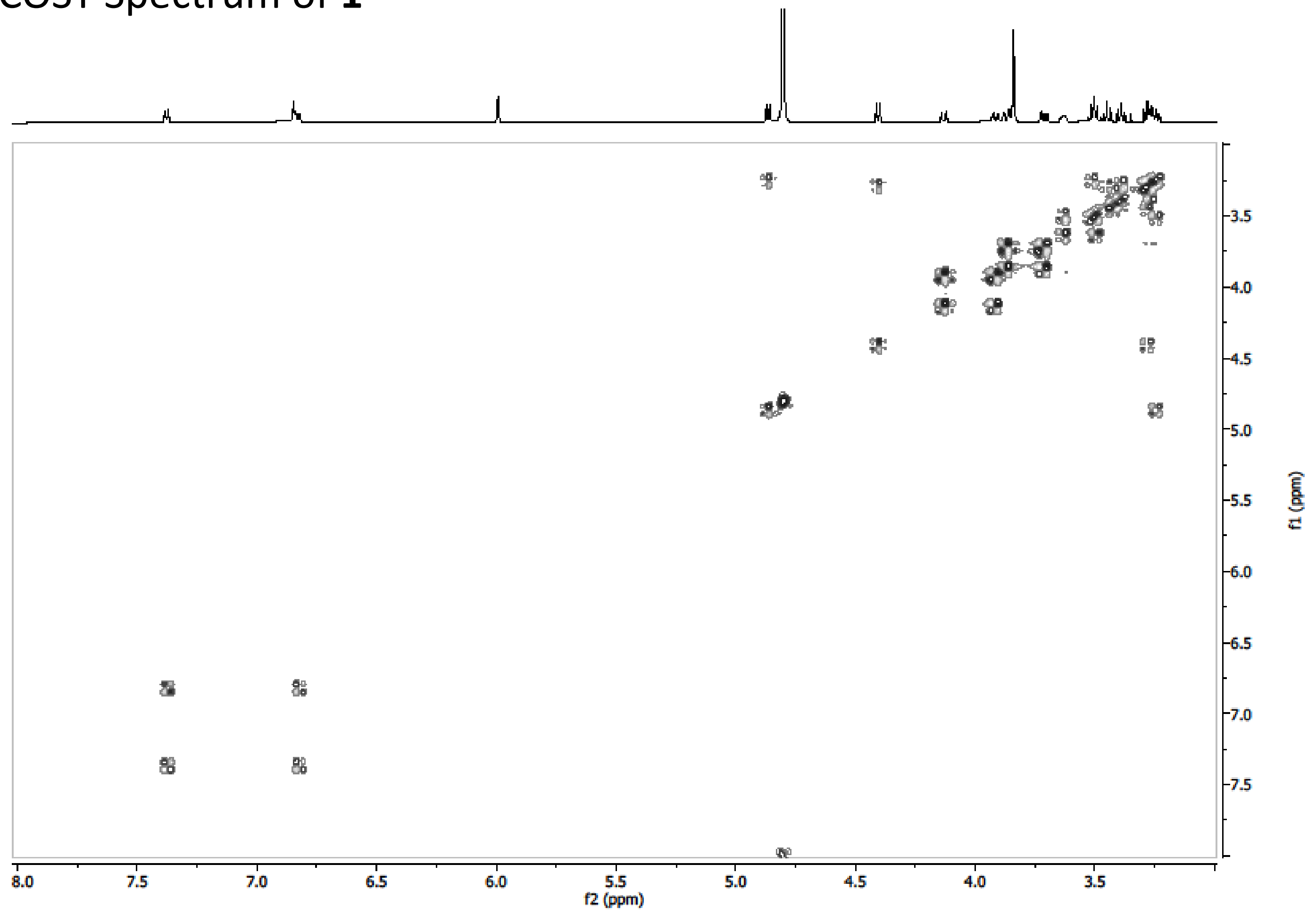

HMBC Spectrum of **1**

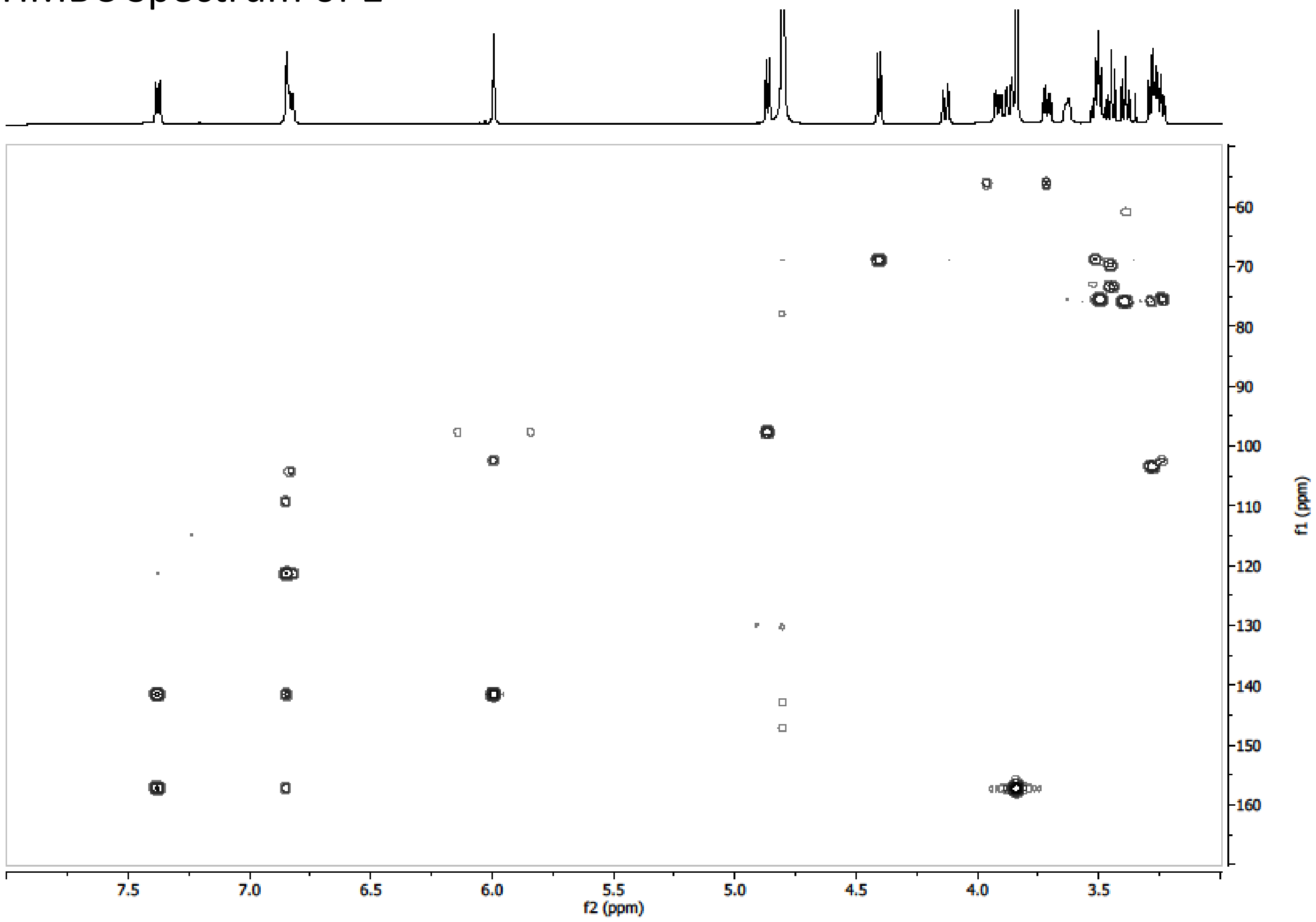

NOESY Spectrum of **1**

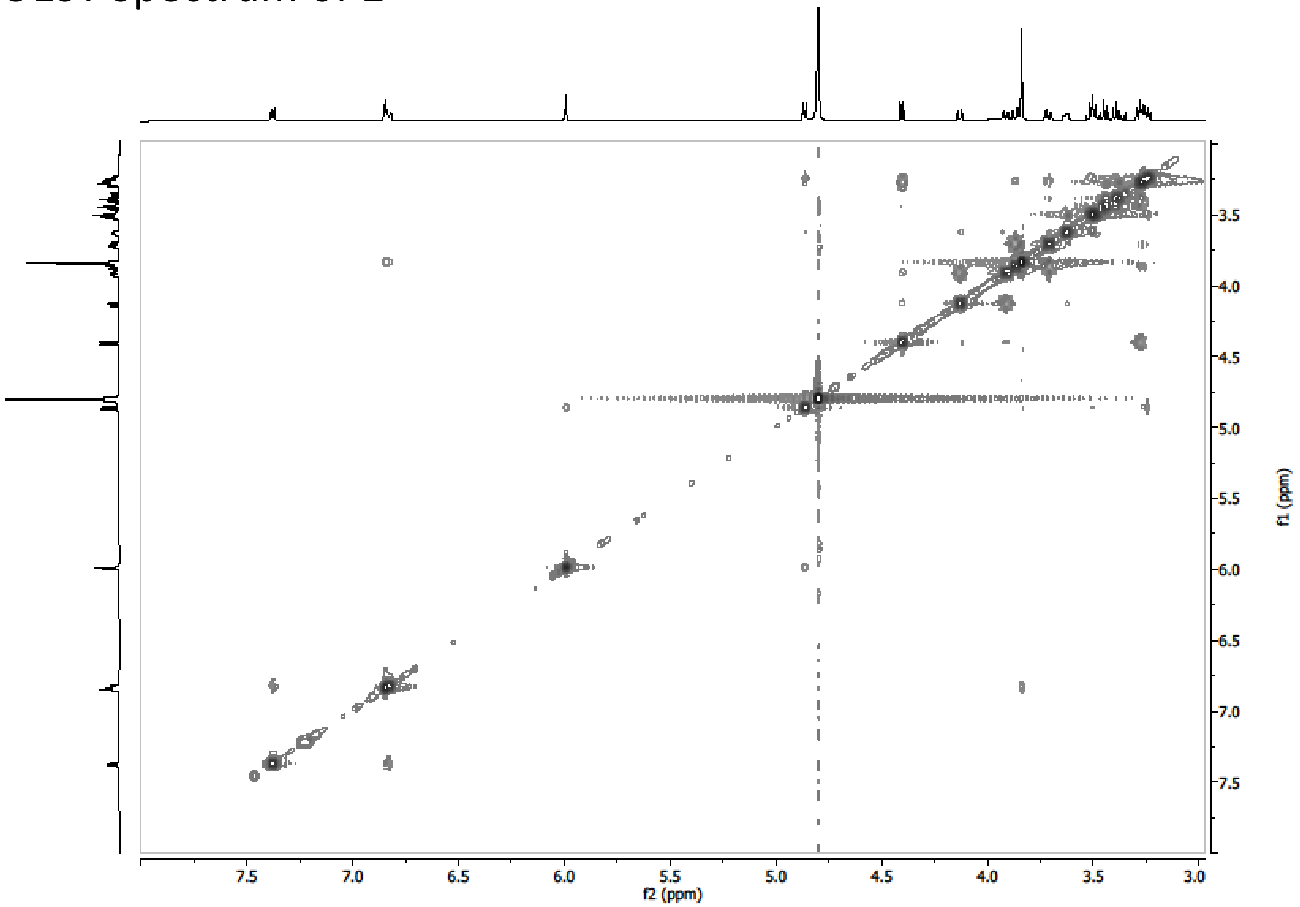

CLIP-HSQC Spectrum of **1**

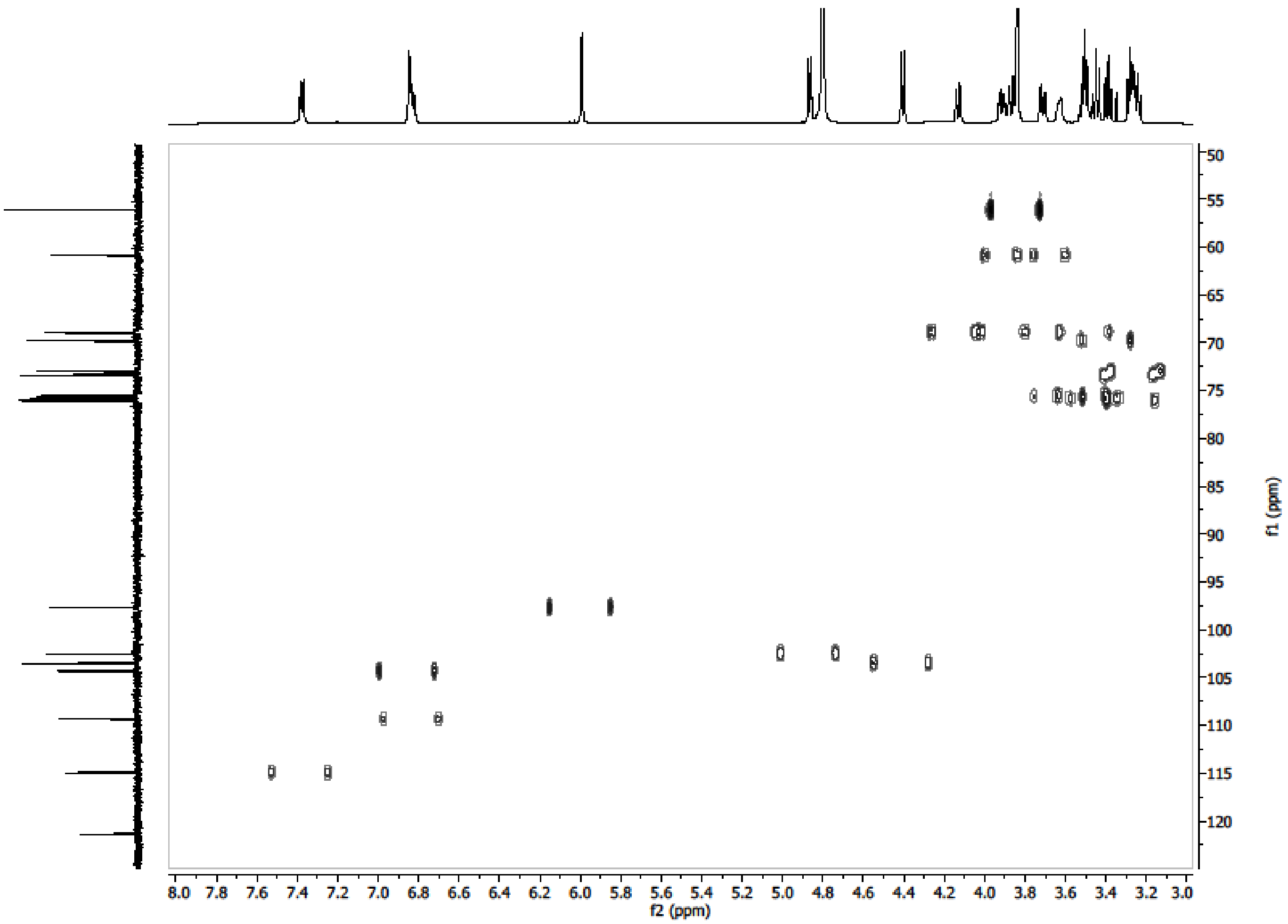

Selective 1D TOCSY Spectrum of **1** irradiation at 4.86 ppm

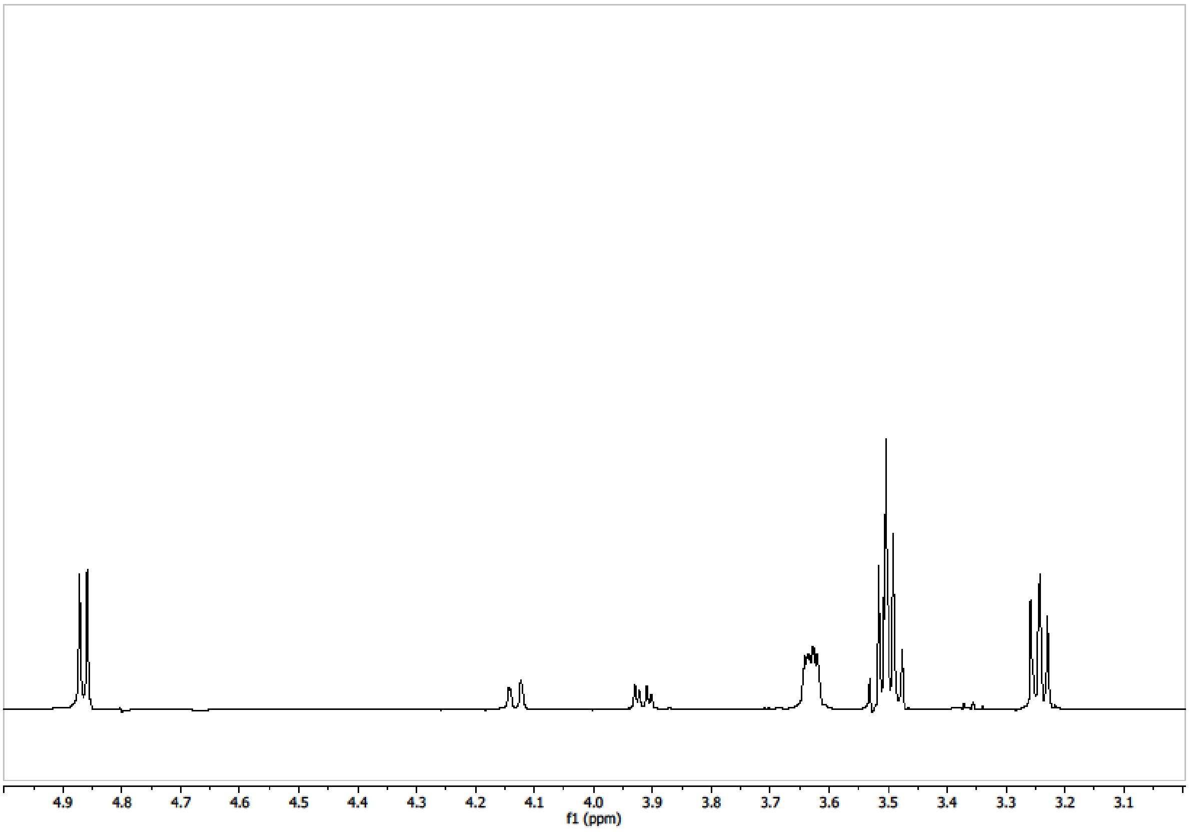

Selective 1D TOCSY Spectrum of **1** irradiation at 4.40 ppm

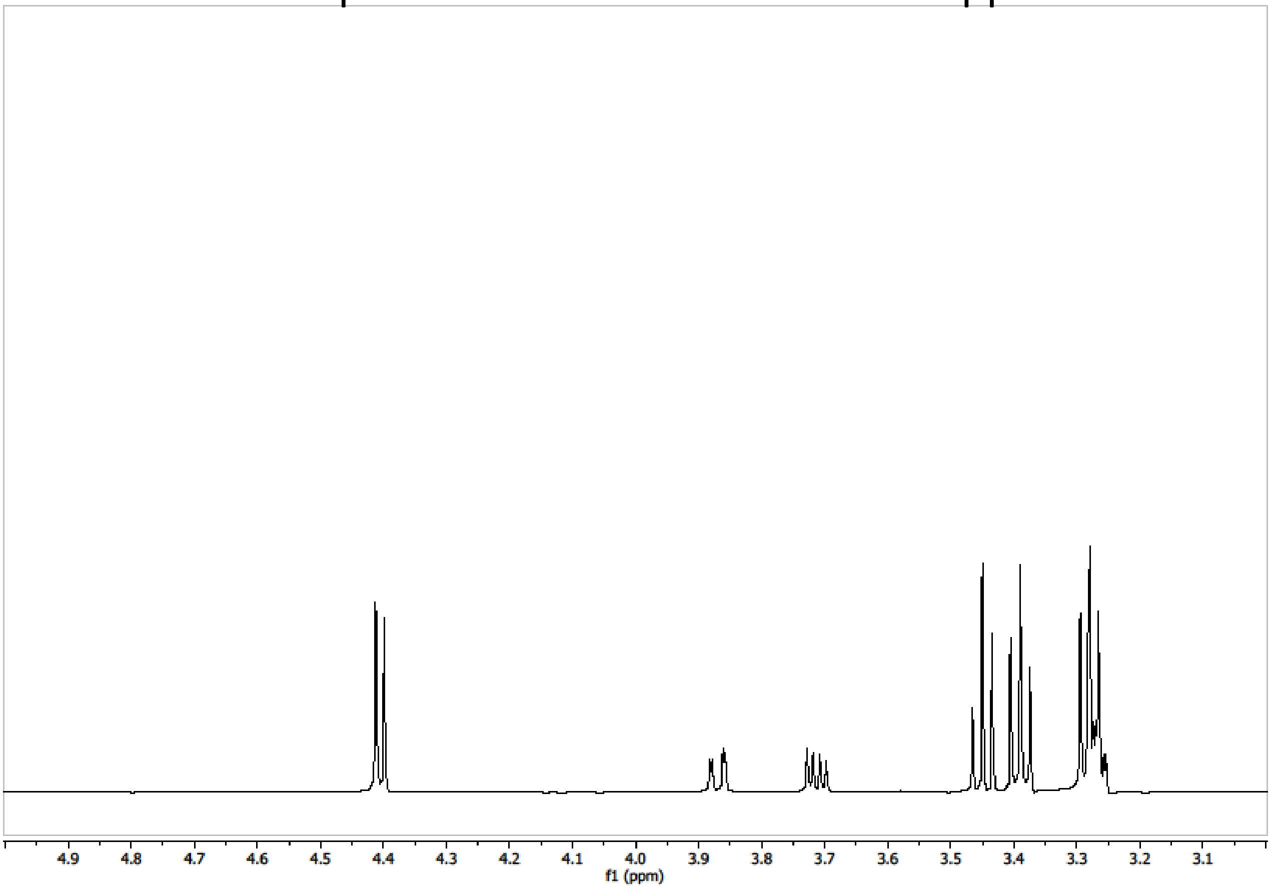

Selective 1D NOESY Spectrum of **1** irradiation at 4.86 ppm

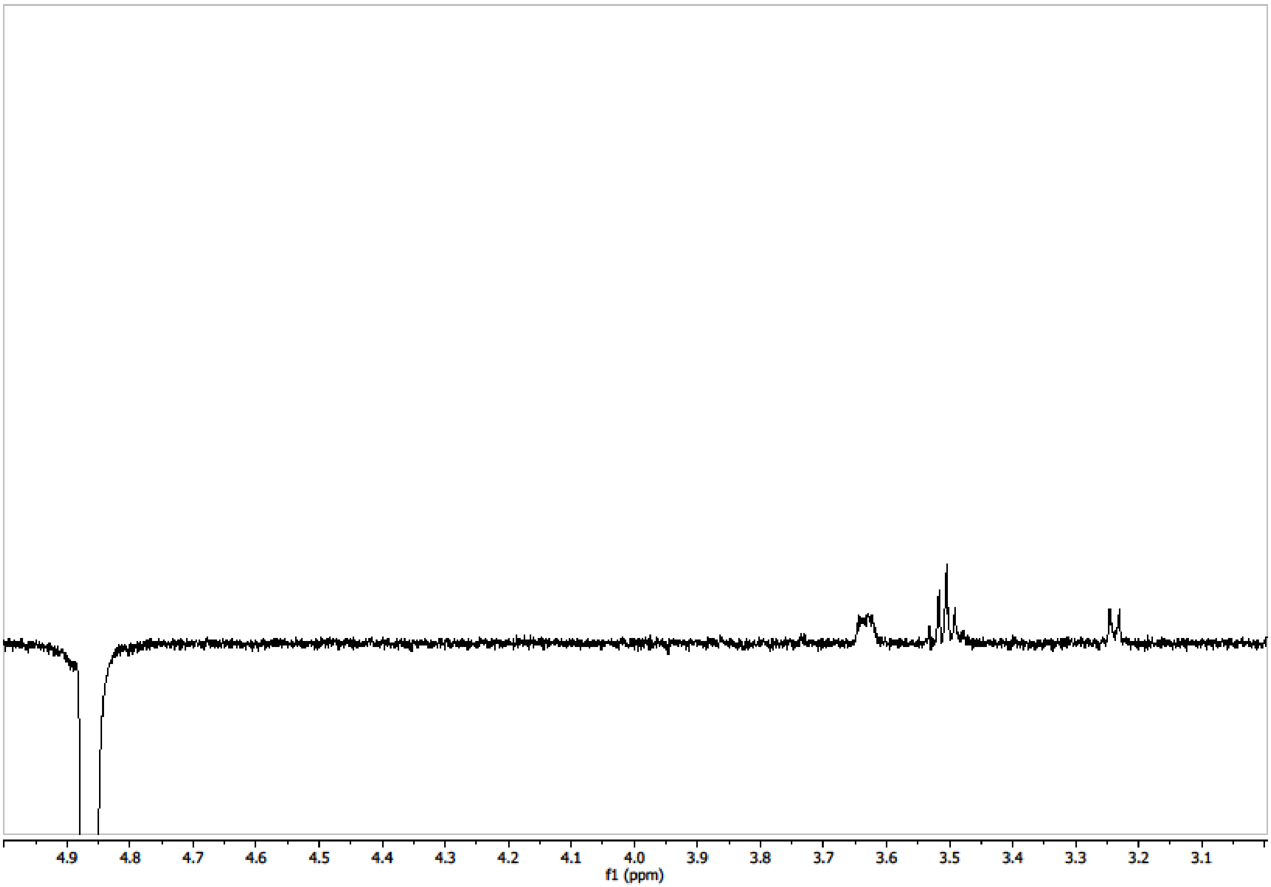

Selective 1D NOESY Spectrum of **1** irradiation at 4.40 ppm

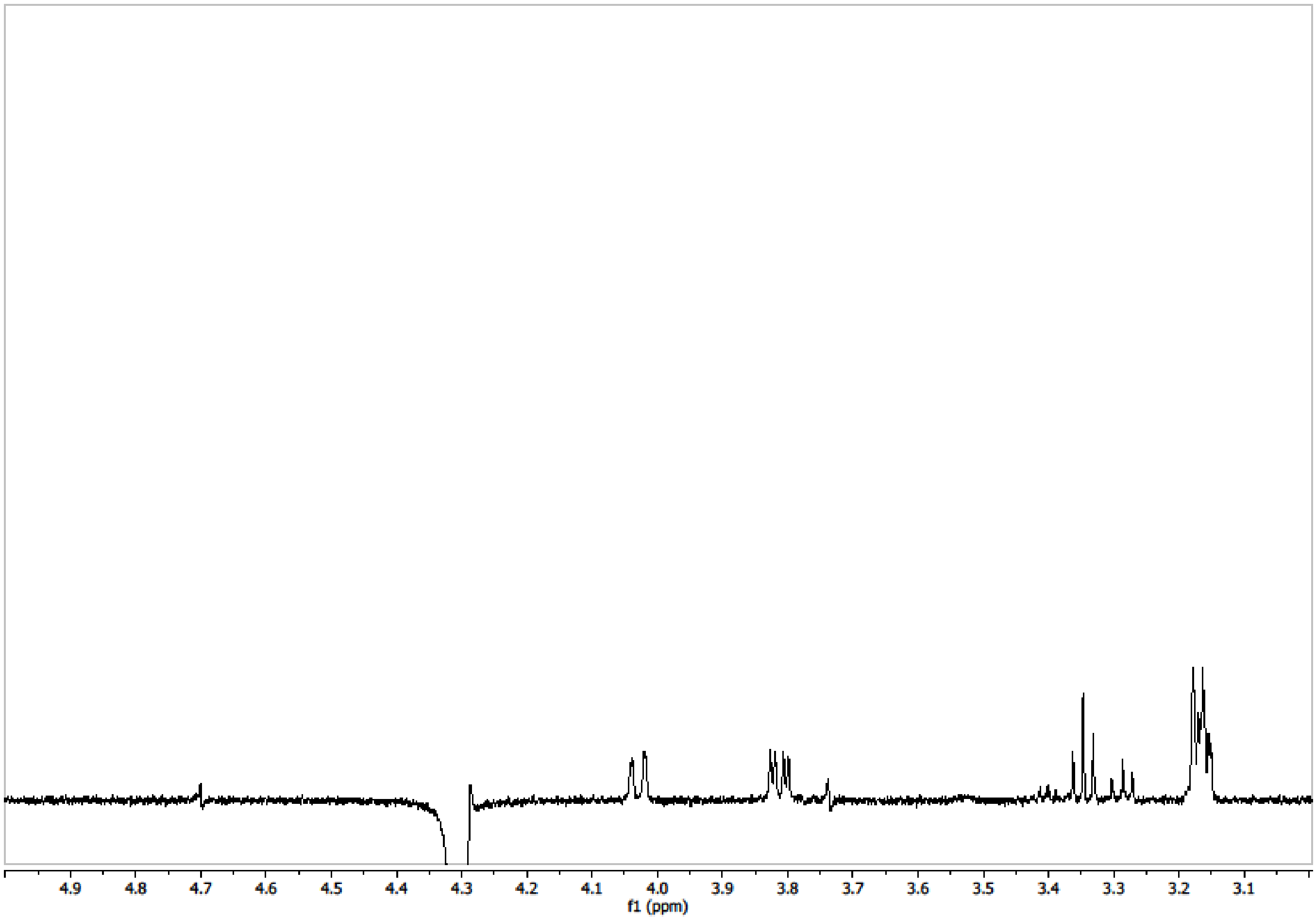

HRMS of **1**

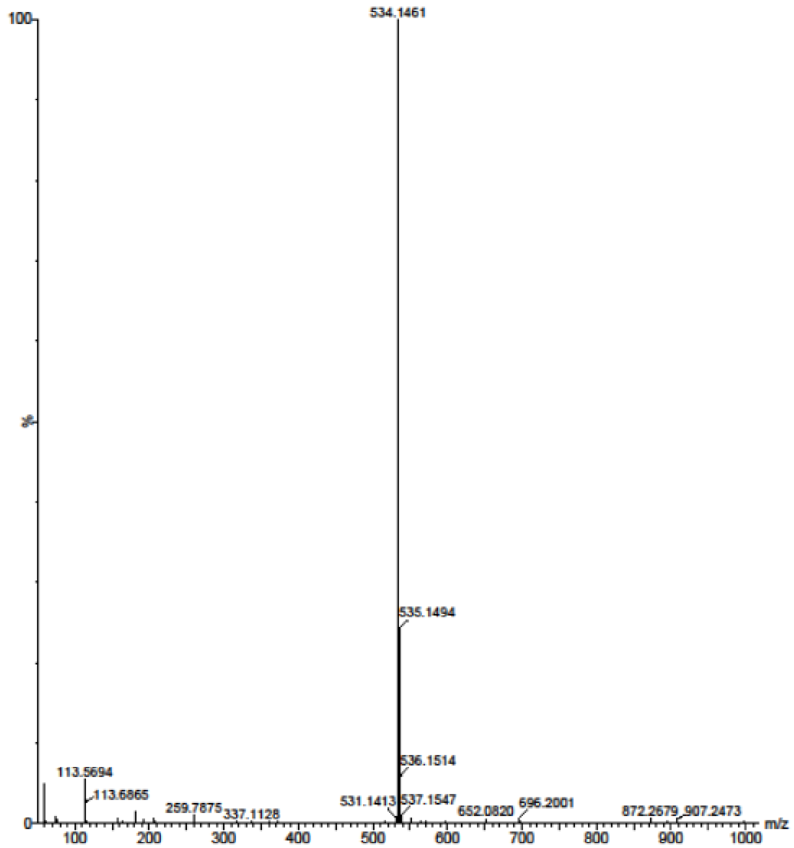

CD Spectrum of **1**

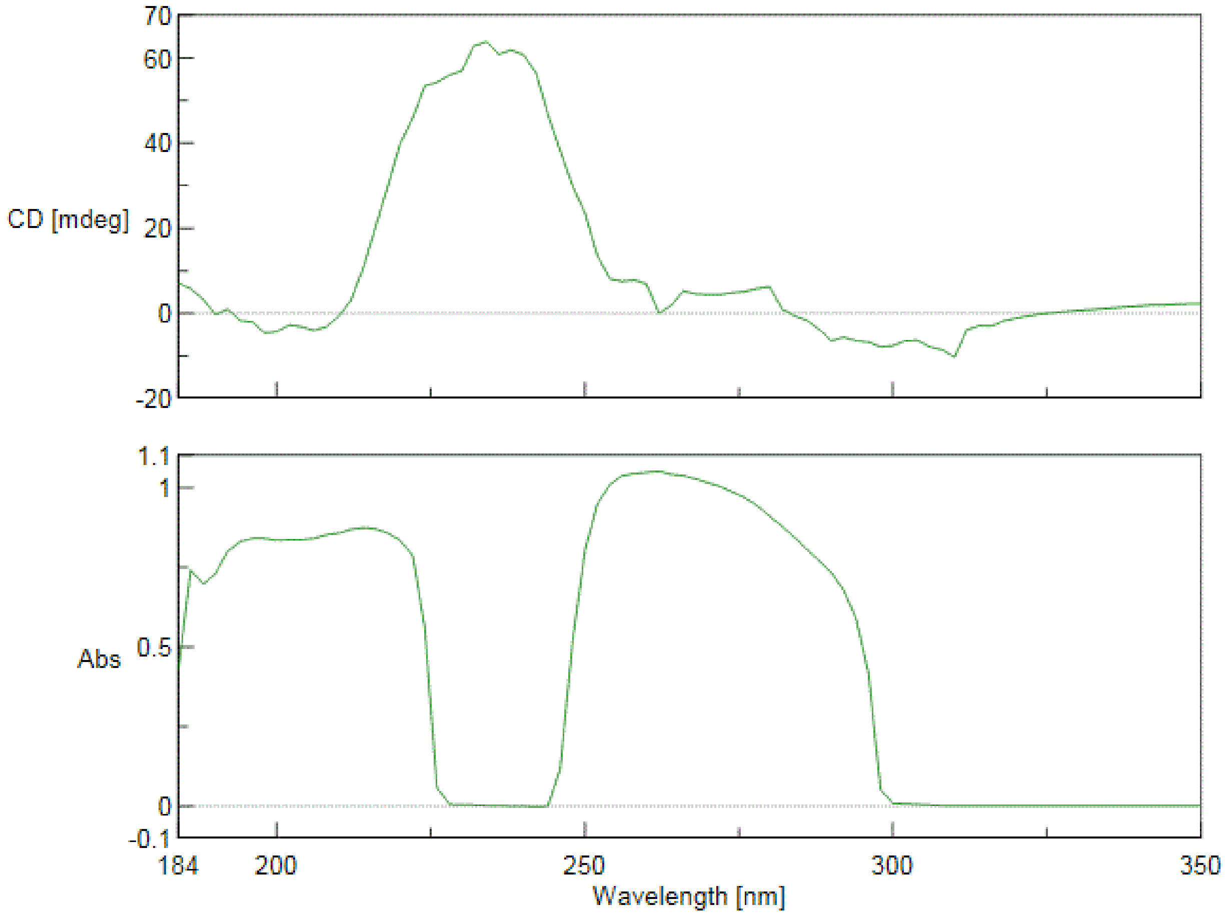

Isolation and structure determination of drought-induced multihexose benzoxazinoids from maize (*Zea mays*)

Supplementary Information SI3:
Structure elucidation of DIMBOA-3Glc (2).

Sylvain Sutour[1,*], Cong Van Doan[2,3,4], Pierre Mateo[2], Tobias Züst[5], Ella Raymonde Hartmann[2], Gaétan Glauser[1], Christelle Aurélie Maud Robert[2,3,*]

[1] Neuchâtel Platform of Analytical Chemistry, University of Neuchâtel, 2000 Neuchâtel, Switzerland

[2] Institute of Plant Sciences, University of Bern, 3013 Bern, Switzerland

[3] Oeschger Centre for Climate Change Research (OCCR), University of Bern, 3012 Bern, Switzerland

[4] Plant Physiology Unit, The Department of Life Sciences and Systems Biology of the University of Turin, Via Accademia Albertina 13, 10123 Torino, Italy

[5] Department of Systematic and Evolutionary Botany, University of Zürich, 8008 Zürich, Switzerland

For correspondence:Sutour Sylvain - sylvain.sutour@unine.ch; Christelle A. M. Robert - christelle.robert@unibe.ch

Chemical data for **2**: ECD (c 0.4 mg/mL, H_2O) λmax (Δε) 196 (4.6), 210 (-4.2), 226 (7.6), 288 (-2.7); ^{1}H NMR (D_2O, 600 MHz) δ 7.47 (1H, d, J = 8.7 Hz, H-5), 6.82 (1H, dd, J = 8.7,2.6, H-6), 6.81 (1H, d, J = 2.6, H-8), 5.92 (1H, s, H-2), 4.85 (1H, d, J = 7.9, H-1'), 4.49 (1H, d, J = 8.0, H-1'''), 4.37 (1H, d, J = 8.8, H-1''), 4.14 (1H, dd, J = 11.6, 2.1, H-6''b), 4.13 (1H, dd, J = 12.2, 2.0, H-6'b), 3.94 (1H, dd, J = 12.2, 4.7, H-6'a), 3.90 (1H, dd, J = 12.1, 2.0, H-6'''b), 3.85 (3H, s, H-11), 3.82 (1H, dd, J = 11.8, 5.2, H-6''a), 3.71 (1H, dd, J = 12.1, 5.9, H-6'''a), 3.64 (1H, ddd, J = 9.7, 4.7, 2.0, H-5'), 3.50 (1H, m, H-3'), 3.49 (1H, m, H-4'), 3.49 (1H, dd, J = 9.3, 9.1, H-3'''), 3.48 (1H, dd, J = 9.7, 9.3, H-3''), 3.43 (1H, dd, J = 9.7, 9.3, H-3''), 3.43 (1H, ddd, J = 10.0, 5.9, 2.0, H-5'''), 3.38 (1H, dd, J = 10.0, 9.1, H-4'''), 3.30 (1H, ddd, J = 9.3, 5.2, 2.1, H-5''), 3.30 (1H, dd, J = 9.3, 8.0, H-2'''), 3.29 (1H, dd, J = 9.3, 8.8, H-2''), 3.26 (1H, dd, J = 9.3, 8.0, H-2'); ^{13}C NMR (D2O, 151 MHz) δ 156.8 (C, C-7), 154.4 (C, C-3), 142.5 (C, C-9), 123.8 (C, C-10), 116.4 (CH, C-5), 109.5 (CH, C-6), 104.7 (CH, C-8), 104.5 (CH, C-1''), 103.7 (CH, C-1'''), 103.3 (CH, C-1'), 98.8 (CH, C-2), 76.6 (CH, C-3'''), 76.5 (CH, C-3''), 76.5 (CH, C-5'''), 76.1 (CH, C-5'), 76.1 (CH, C-3'), 75.6 (CH, C-5''), 74.0 (CH, C-2'''), 73.9 (CH, C-2''), 73.6 (CH, C-2'), 70.3 (CH, C-4''), 70.2 (CH, C-4'), 69.9 (CH2, C-6'), 69.6 (CH2, C-4'''), 69.1 (CH2, C-6''), 61.5 (CH2, C-6'''), 56.5 (CH3, C-11). HRESIMS m/z 696.1987 (calcd for C27H38NO20, 696.1987).

^{1}H NMR Spectrum of **2** (D_2O, 600 MHz)

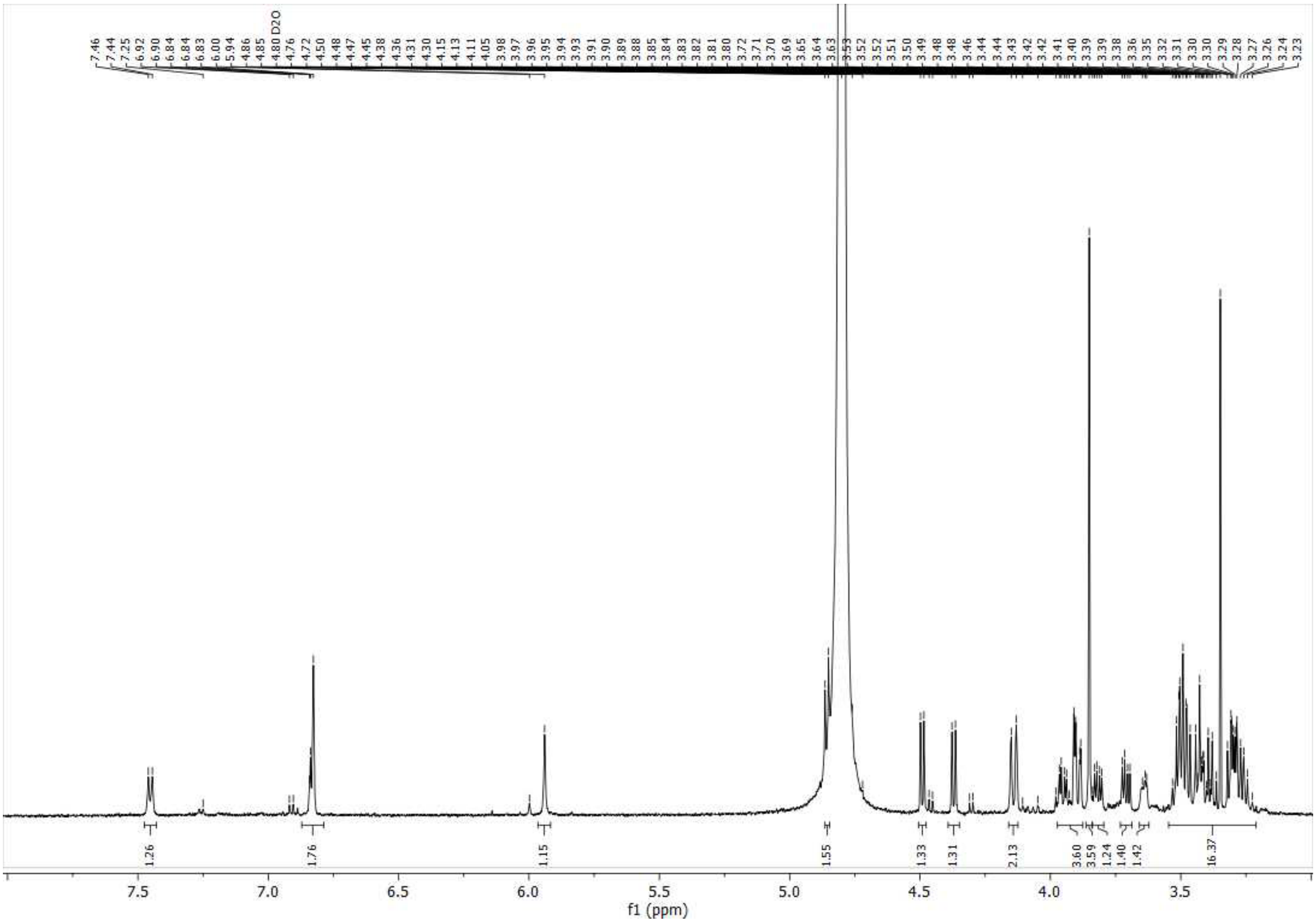

HSQC Spectrum of **2**

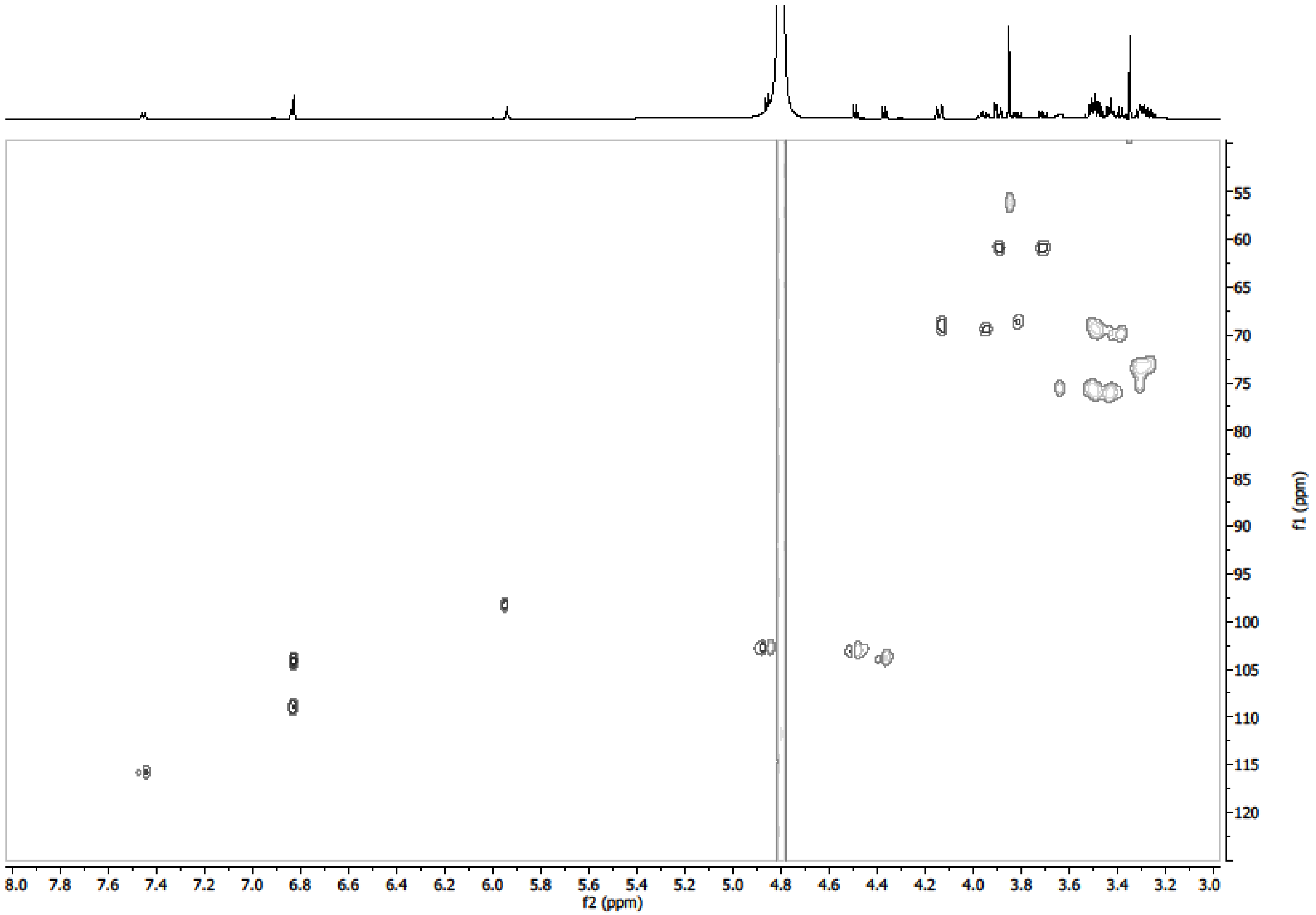

Band selective HSQC Spectrum of **2**

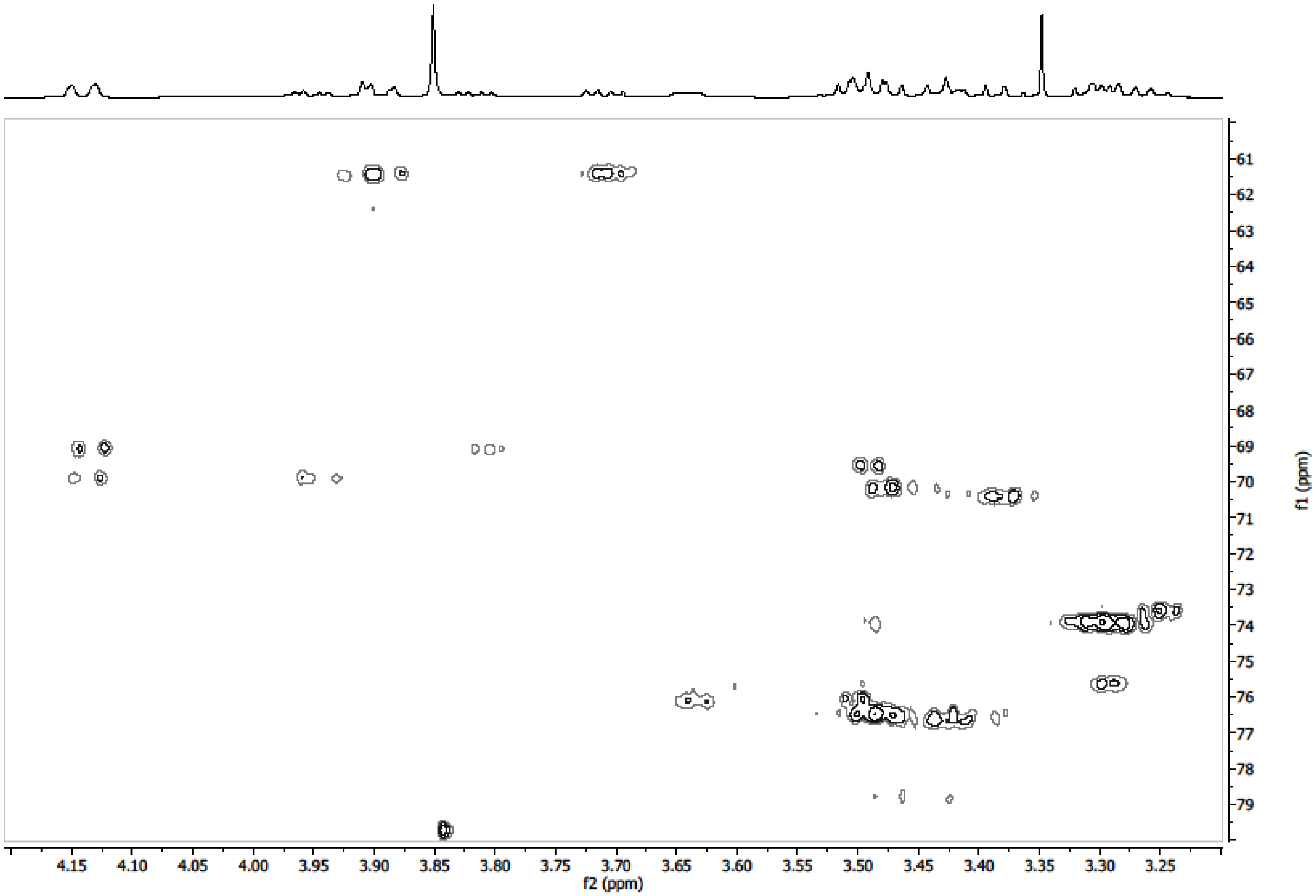

HMBC Spectrum of **2**

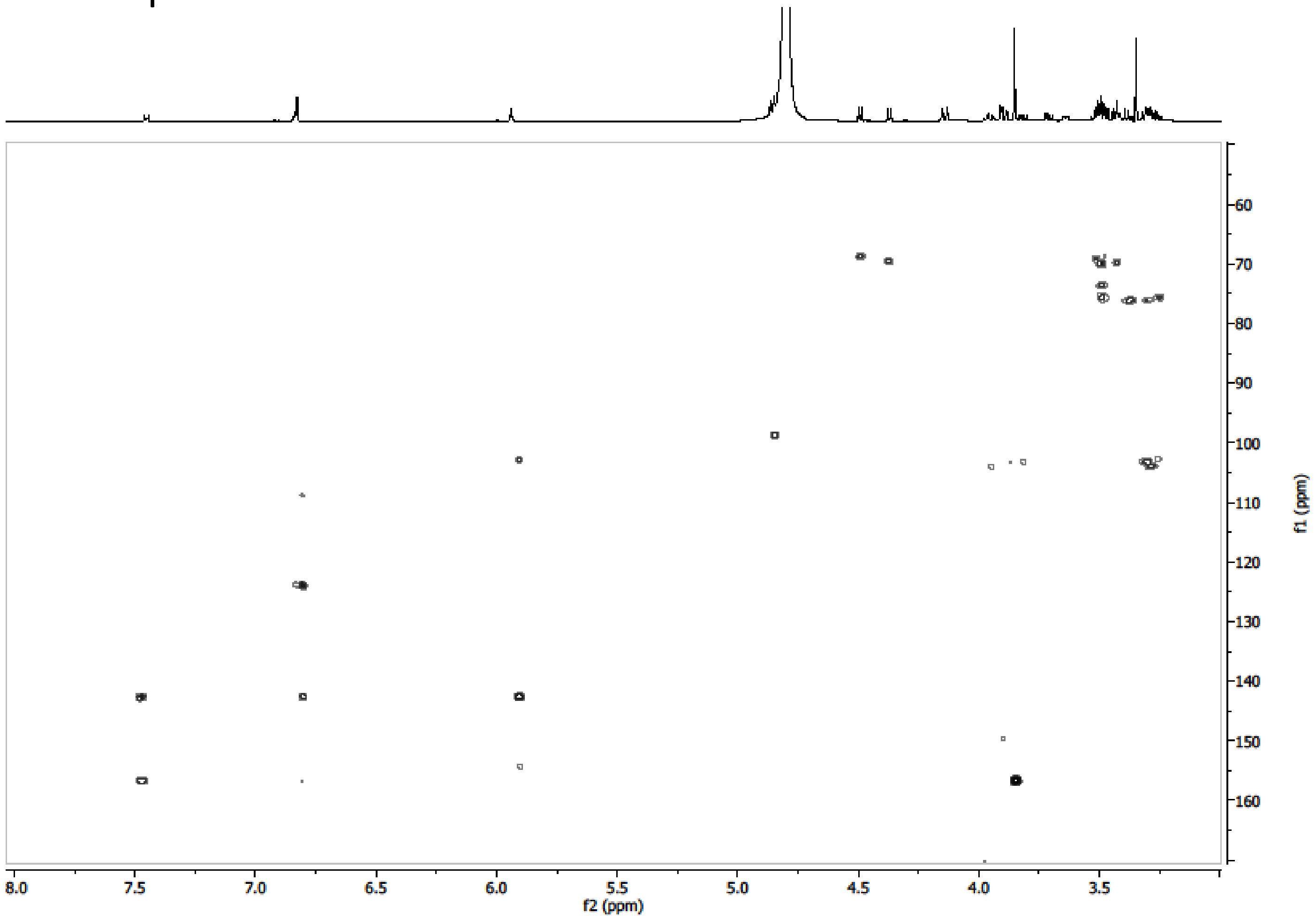

CLIP HSQC Spectrum of **2**

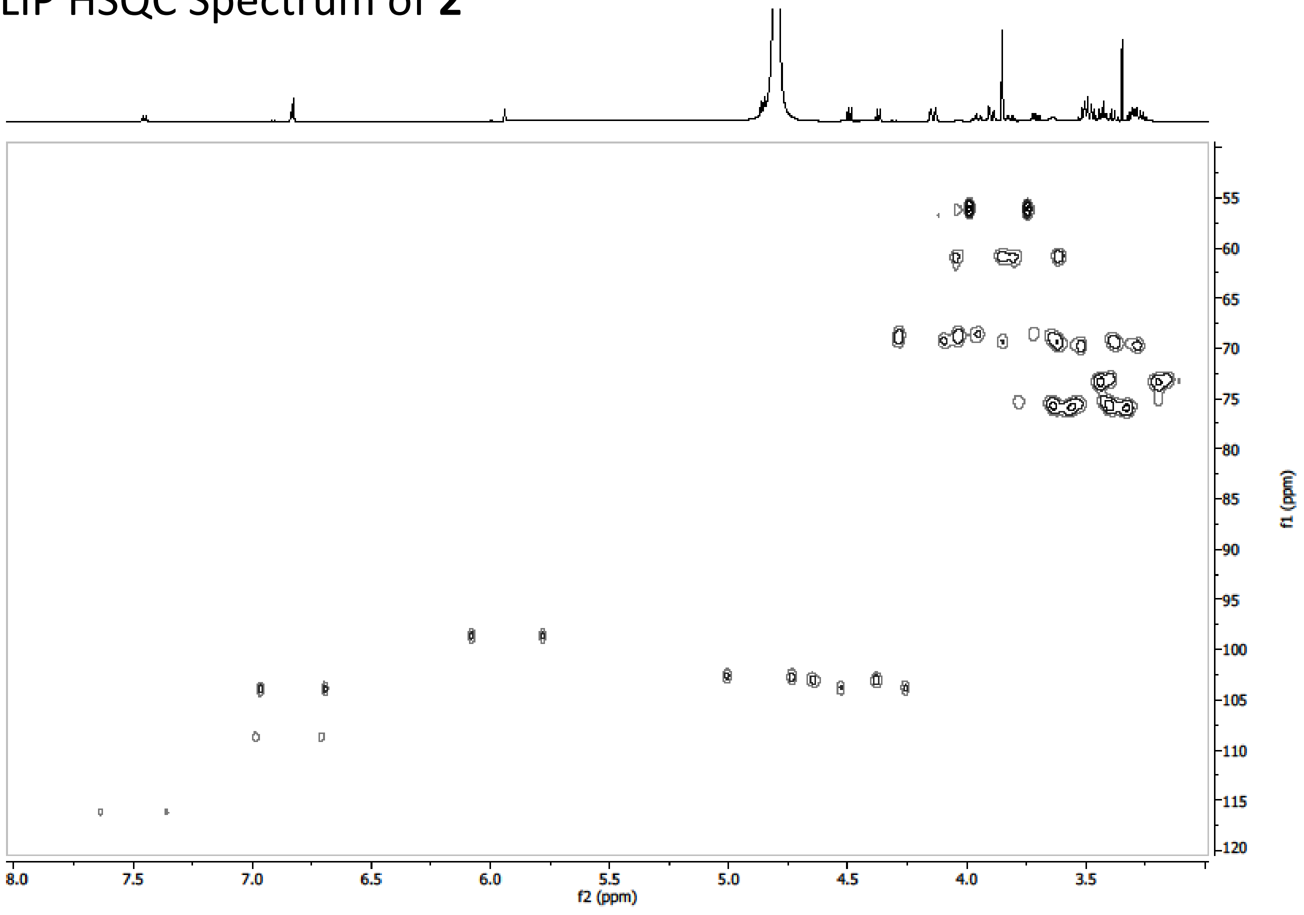

Selective 1D TOCSY Spectrum of **2** irradiation at 4.85 ppm

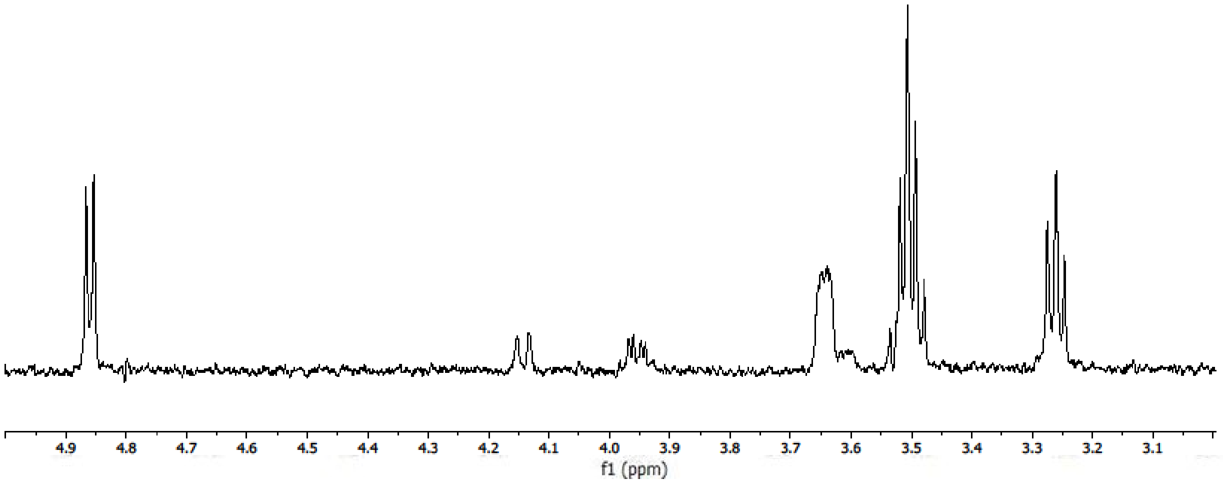

Selective 1D TOCSY Spectrum of **2** irradiation at 4.49 ppm

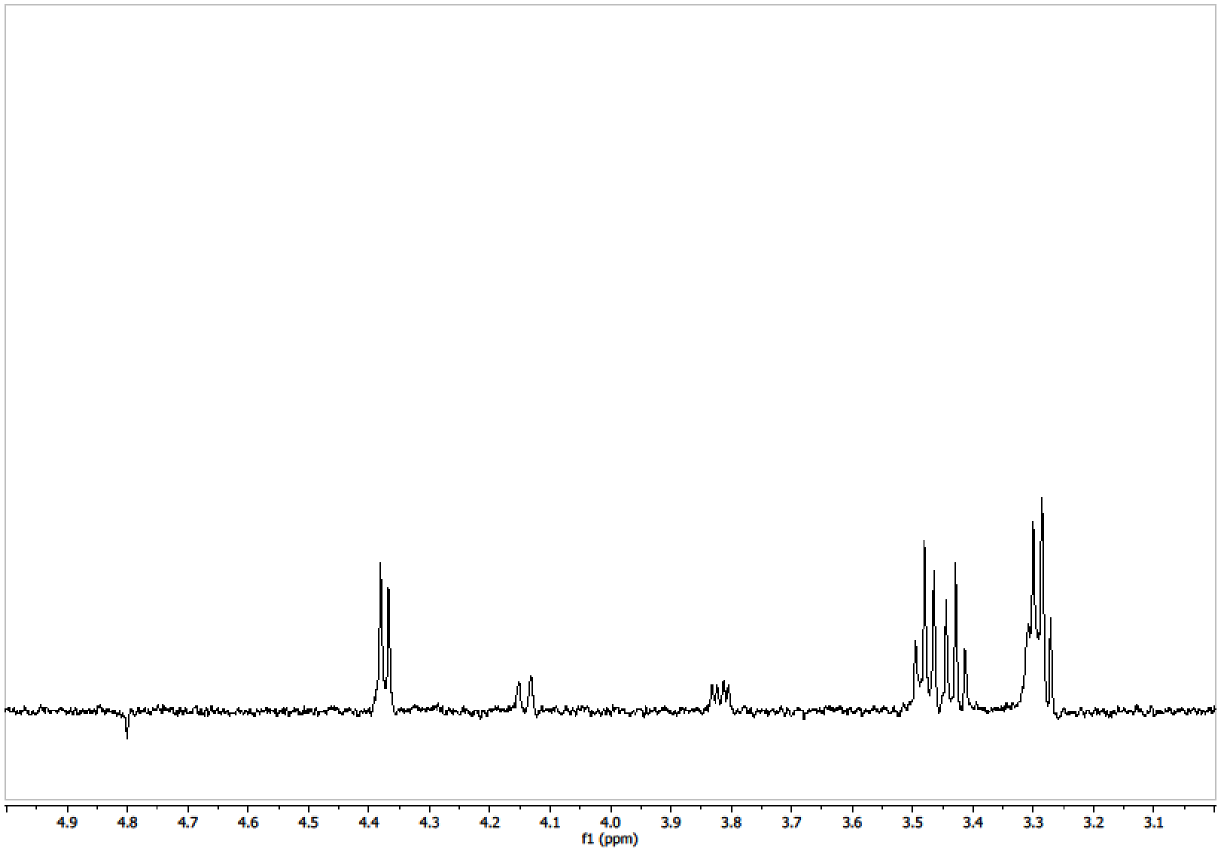

Selective 1D TOCSY Spectrum of **2** irradiation at 4.37 ppm

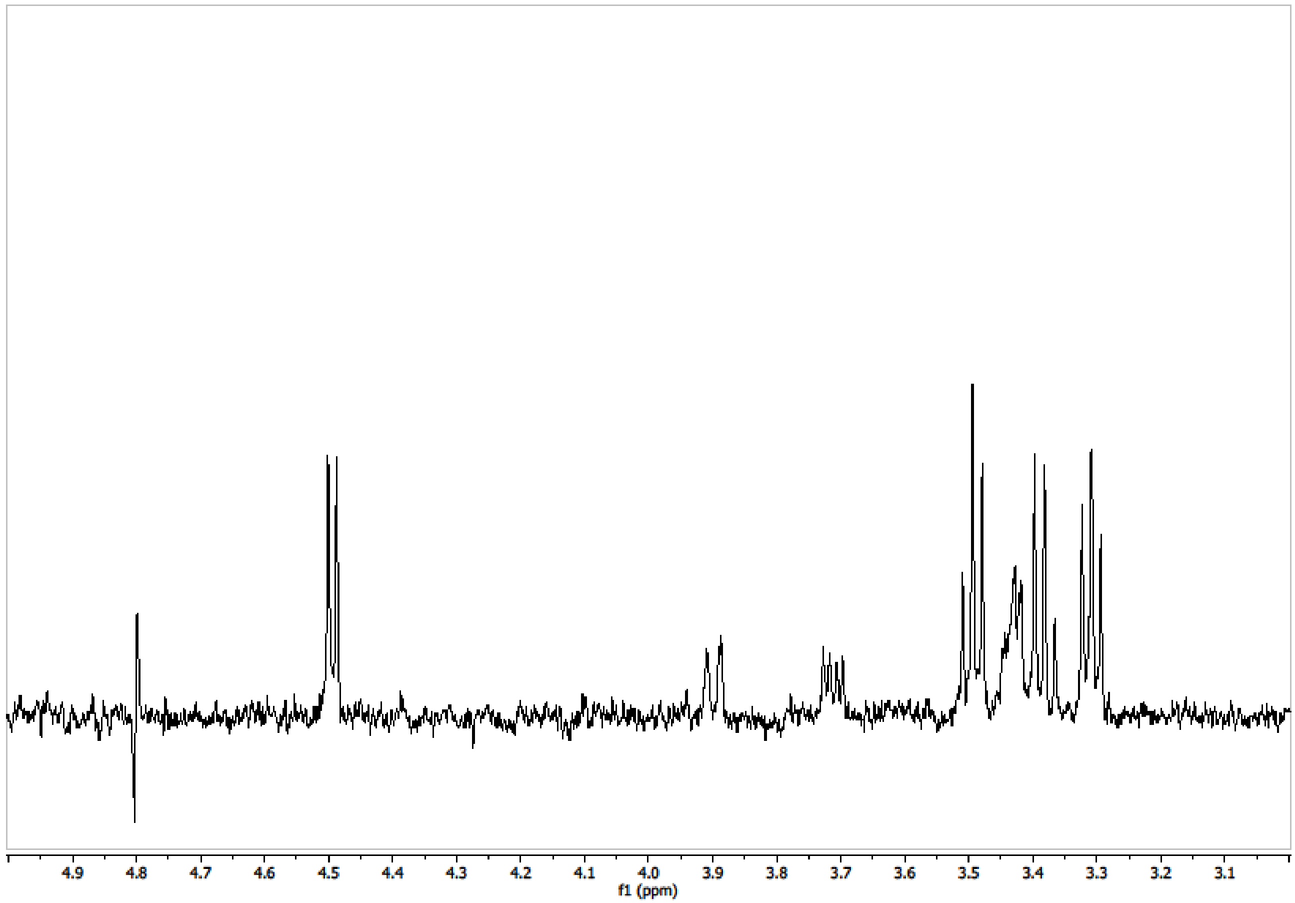

HRMS of **2**

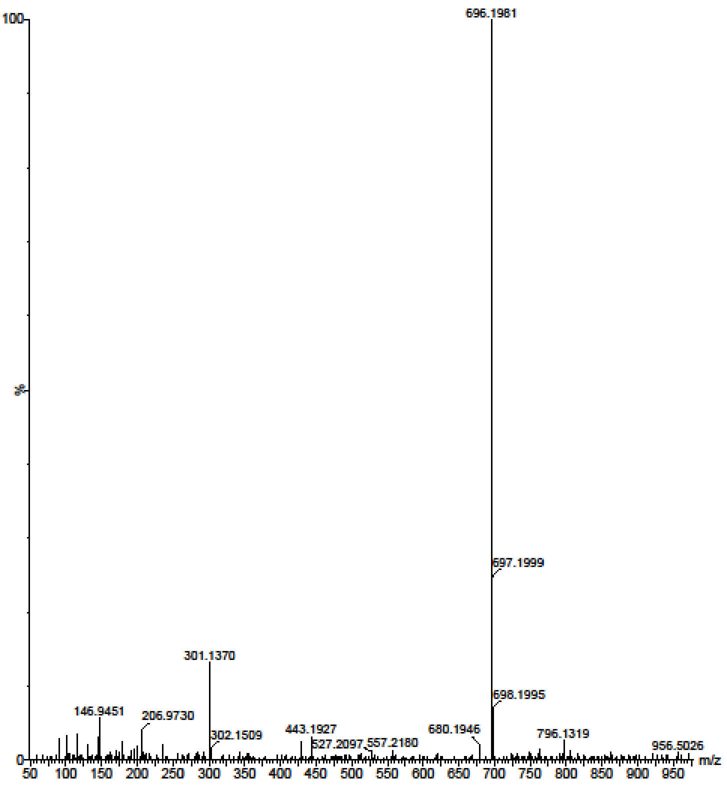

CD Spectrum of **2**

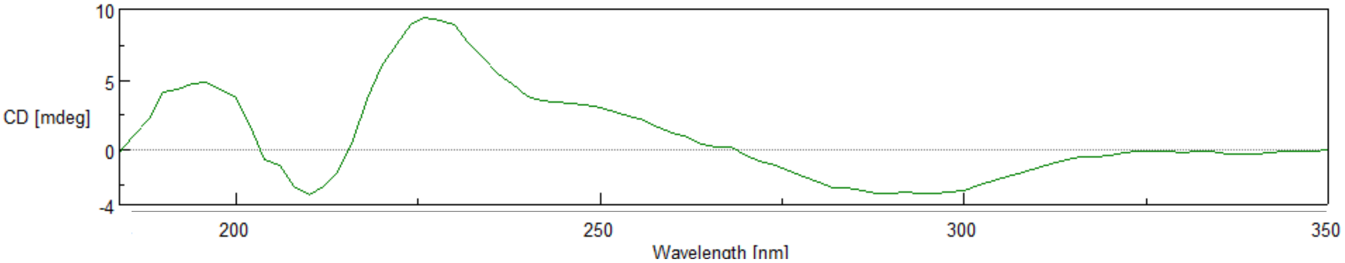

Isolation and structure determination of drought-induced multihexose benzoxazinoids from maize (*Zea mays*)

Supplementary Information SI4:
Structure elucidation of HMBOA-2Glc (3).

Sylvain Sutour[1,*], Cong Van Doan[2,3,4], Pierre Mateo[2], Tobias Züst[5], Ella Raymonde Hartmann[2], Gaétan Glauser[1], Christelle Aurélie Maud Robert[2,3,*]

[1] Neuchâtel Platform of Analytical Chemistry, University of Neuchâtel, 2000 Neuchâtel, Switzerland

[2] Institute of Plant Sciences, University of Bern, 3013 Bern, Switzerland

[3] Oeschger Centre for Climate Change Research (OCCR), University of Bern, 3012 Bern, Switzerland

[4] Plant Physiology Unit, The Department of Life Sciences and Systems Biology of the University of Turin, Via Accademia Albertina 13, 10123 Torino, Italy

[5] Department of Systematic and Evolutionary Botany, University of Zürich, 8008 Zürich, Switzerland

For correspondence:Sutour Sylvain - sylvain.sutour@unine.ch; Christelle A. M. Robert - christelle.robert@unibe.ch

Chemical data for **3**: ECD (c 1.1 mg/mL, H_2O) λmax (Δε) 192 (19.4), 206 (-15.9), 232 (12.4), 284 (-1.7); ^{1}H NMR (D_2O, 600 MHz) δ 7.01 (1H, d, J = 8.8 Hz, H-5), 6.86 (1H, d, J = 2.8 Hz, H-8), 6.76 (1H, dd, J = 8.8, 2.8 Hz, H-6), 5.84 (1H, s, H-2), 4.86 (1H, d, J = 8.1 Hz, H-1'), 4.41 (1H, d, J = 8.0 Hz, H-1''), 4.13 (1H, dd, J = 12.1, 1.8 Hz, H-6'b), 3.91 (1H, dd, J = 12.1, 4.6 Hz, H-6'a), 3.88 (1H, dd, J = 12.3, 2.2 Hz, H-6''b), 3.83 (3H, s, OMe H-11), 3.72 (1H, dd, J = 12.3, 5.7 Hz, H-6''a), 3.61 (1H, ddd, J = 9.5, 4.6, 1.8 Hz, H-5'), 3.51 (1H, m, H-3'), 3.50 (1H, m, H-4'), 3.45 (1H, dd, J = 9.3, 9.1 Hz, H-3''), 3.39 (1H, dd, J = 9.5, 9.1 Hz, H-4''), 3.29 (1H, dd, J = 9.3, 8.0 Hz, H-2''), 3.27 (1H, ddd, J = 9.5, 5.7, 2.2 Hz, H- 5''), 3.25 (1H, dd, J = 8.9, 8.1 Hz, H-2'); ^{13}C NMR (D_2O, 151 MHz) δ 161.8 (C, C-3), 156.7 (C, C-7), 141.2 (C, C-9), 118.8 (C, C-10), 117.5 (CH, C-5), 109.8 (CH, C-6), 104.4 (CH, C-8), 103.7 (CH, C-1''), 102.5 (CH, C-1'), 96.1 (CH, C-2), 76.3 (CH, C-5''), 76.1 (CH, C-3''), 75.8 (CH, C-5'), 75.7 (CH, C-3'), 73.5 (CH, C-2''), 73.1 (CH, C-2'), 70.0 (CH, C-4''), 69.2 (CH, C-4'), 69.0 (CH2, C-6'), 61.0 (CH2, C-6''), 56.4 (CH3, C-11). HRESIMS m/z 518.1517 (calcd for C21H28NO14, 518.1510).

^{1}H NMR Spectrum of **3** (D_2O, 600 MHz)

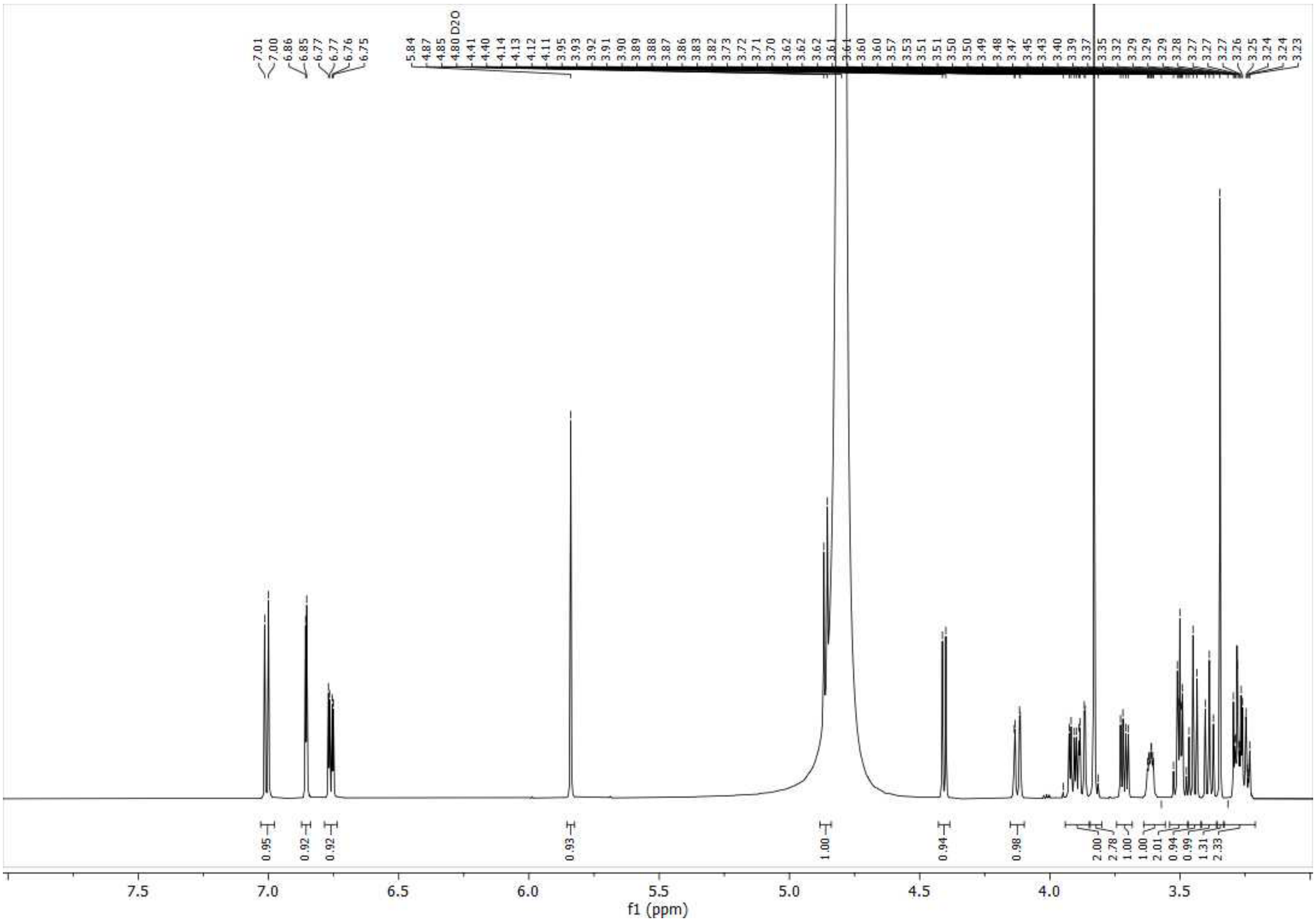

HSQC Spectrum of **3**

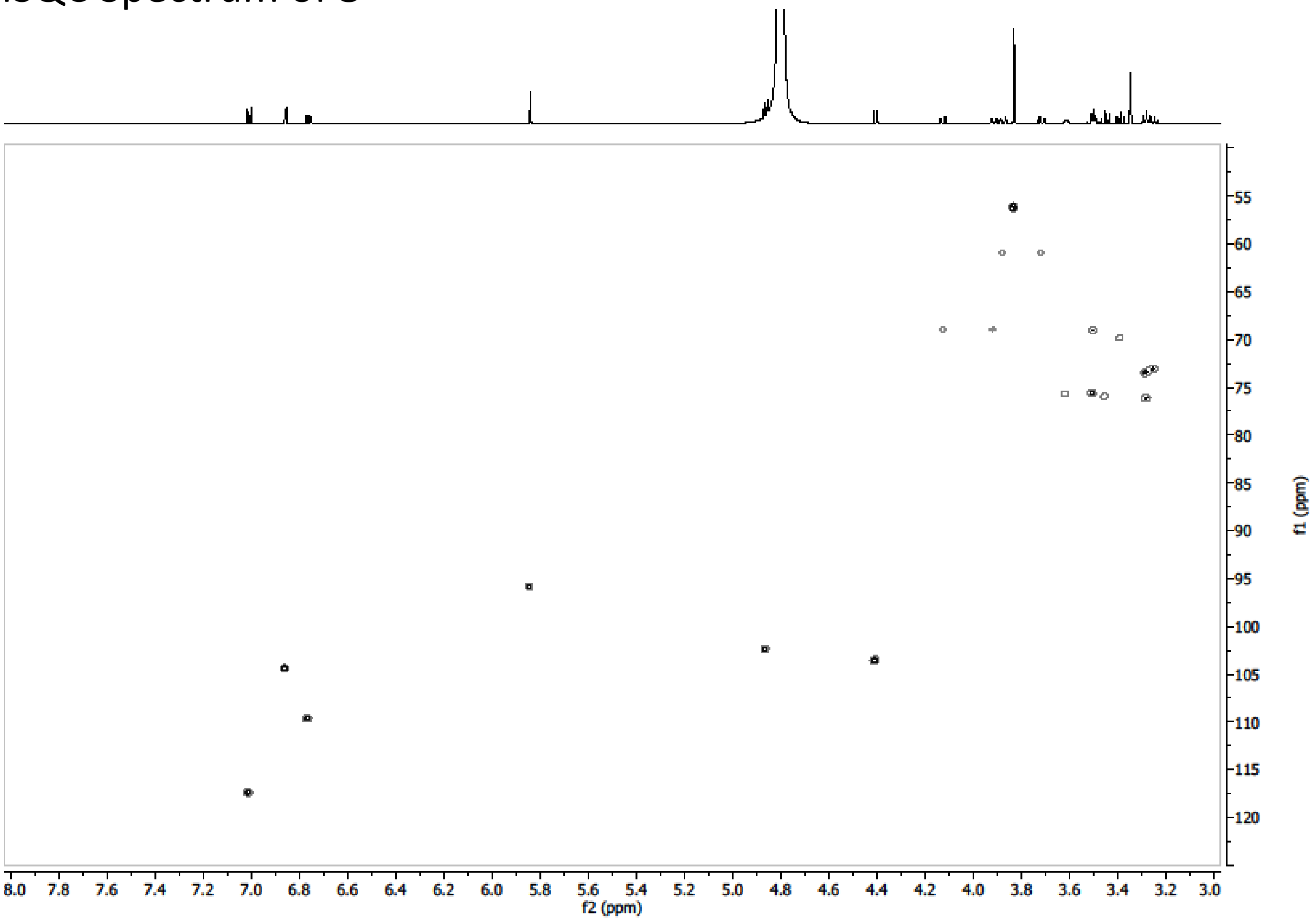

Band selective HSQC Spectrum of **3**

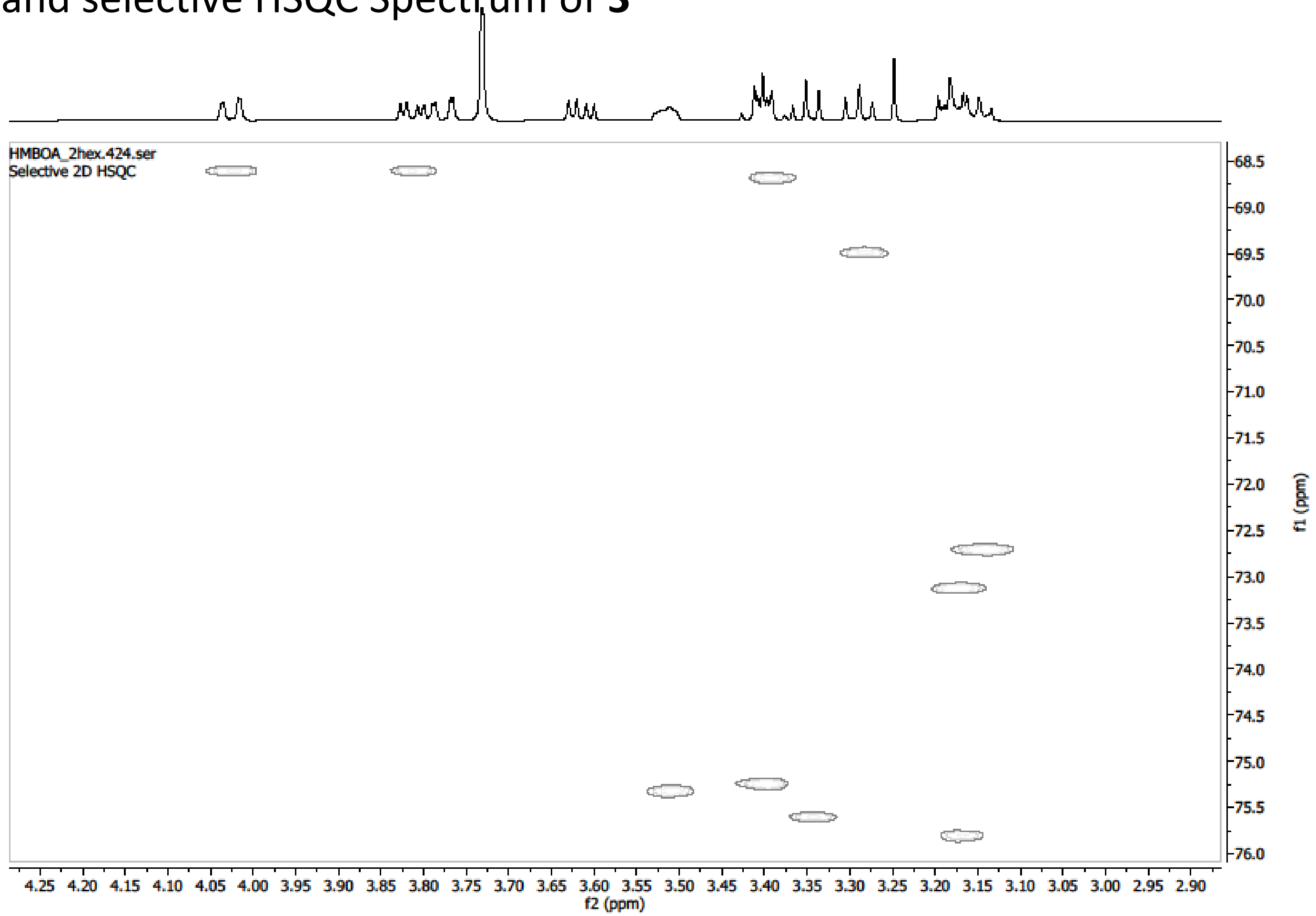

HMBC Spectrum of **3**

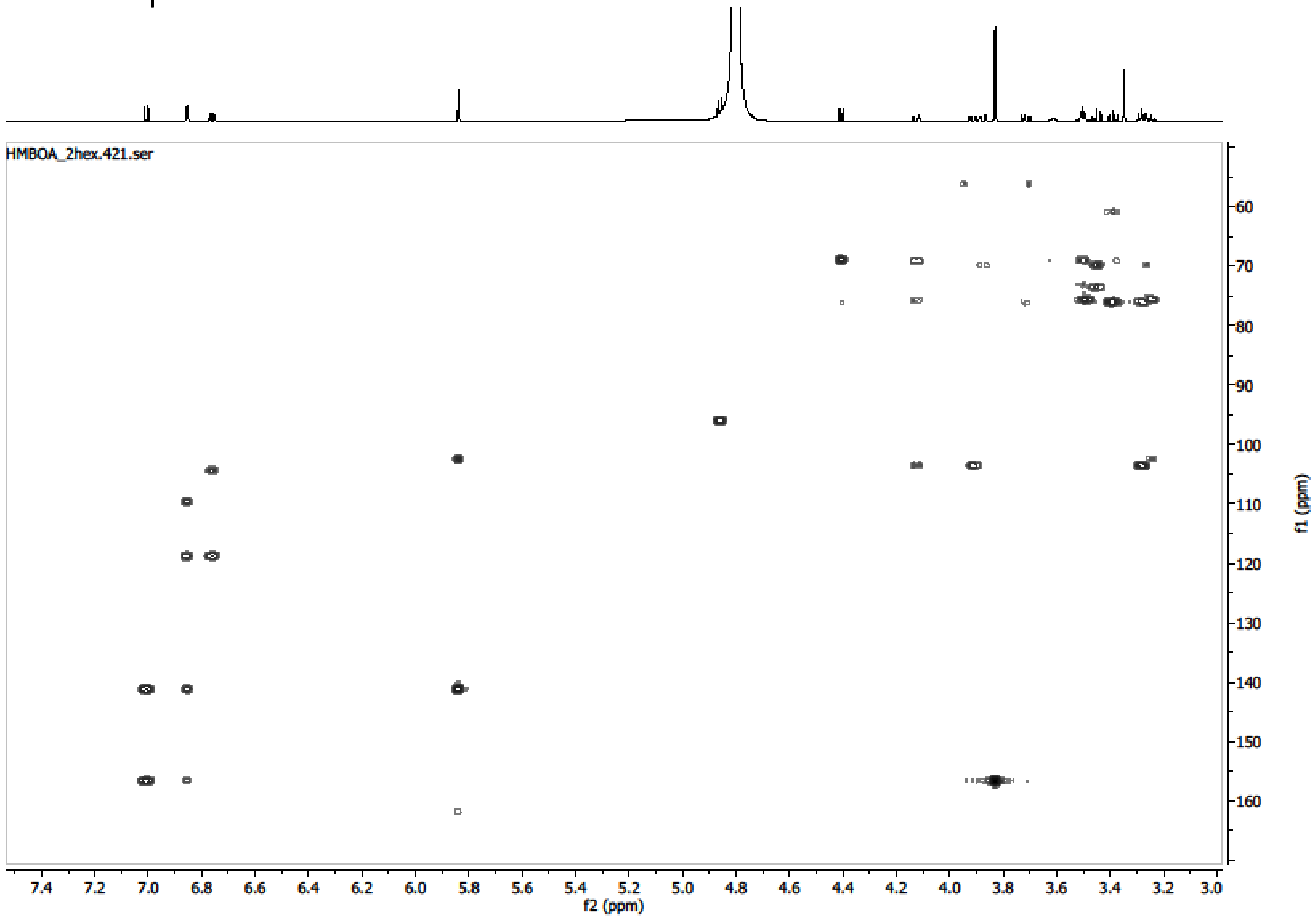

CLIP HSQC Spectrum of **3**

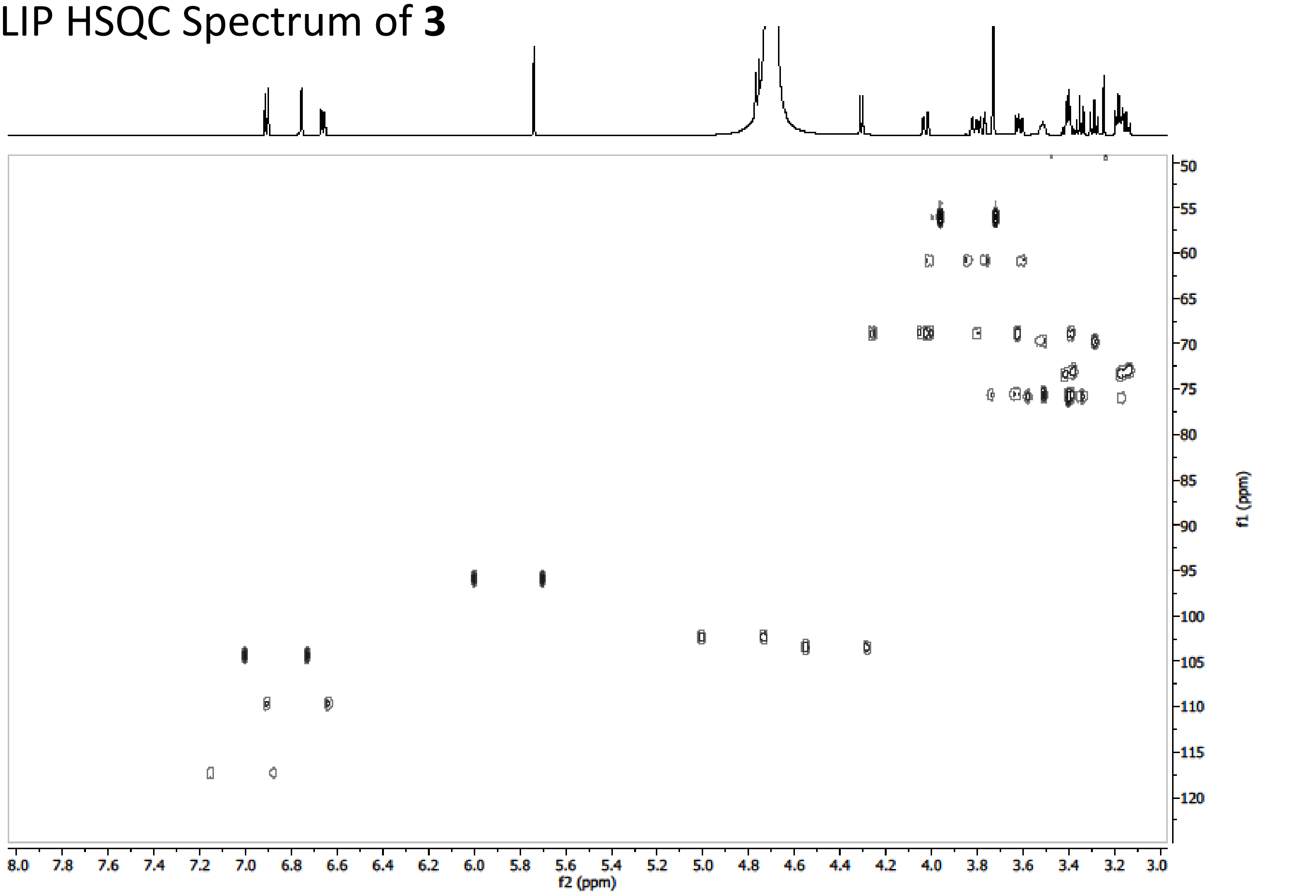

Selective 1D TOCSY Spectrum of **3** irradiation at 4.86 ppm

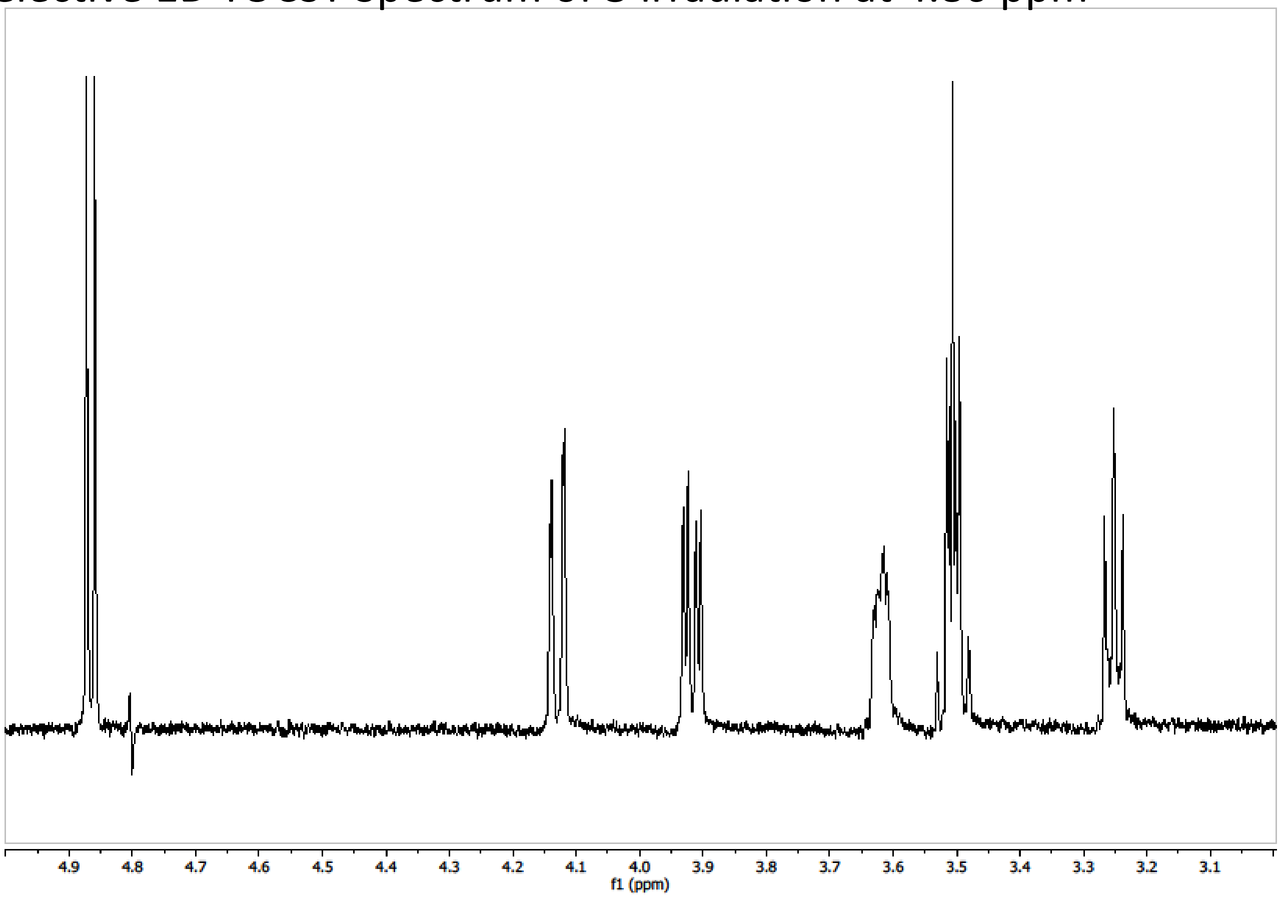

Selective 1D TOCSY Spectrum of **3** irradiation at 4.41 ppm

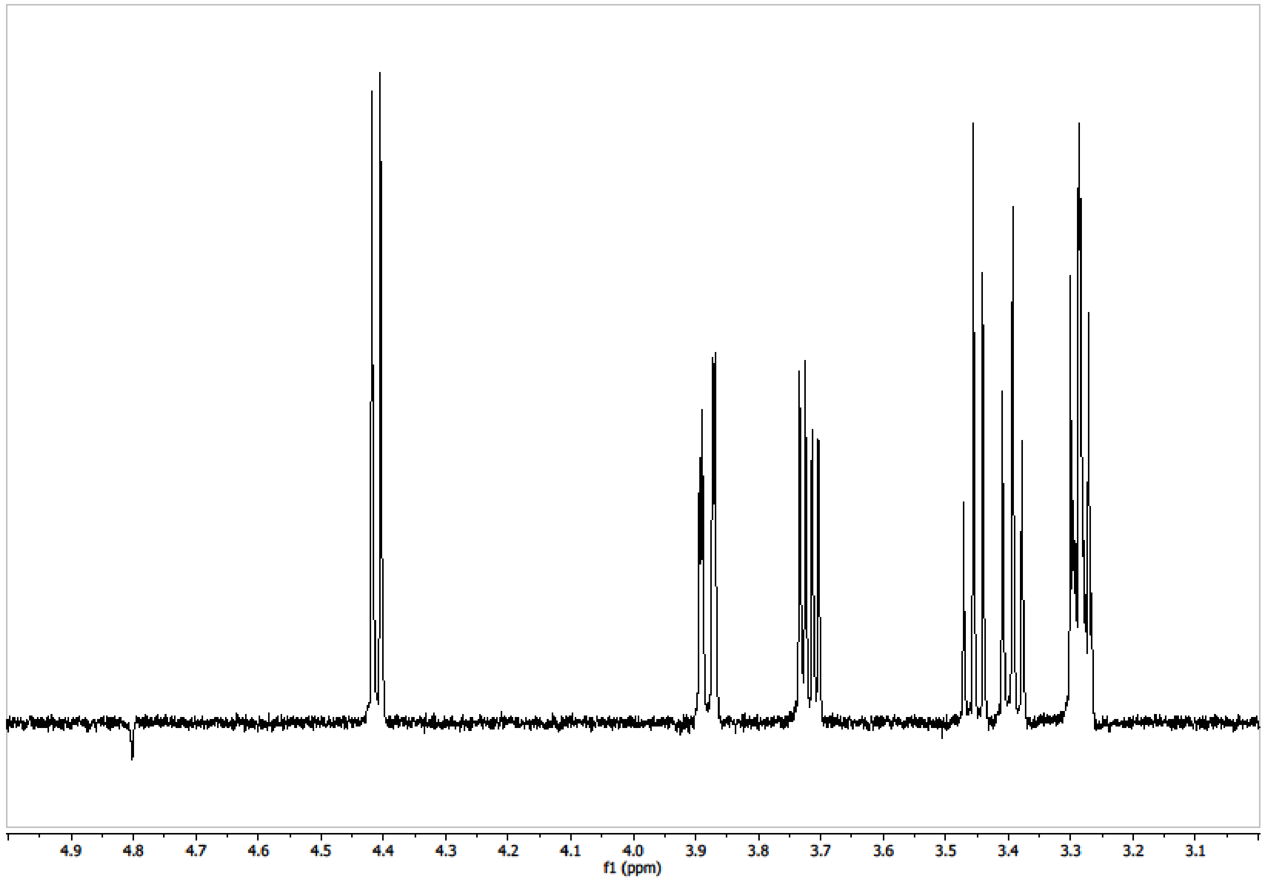

Selective 1D NOESY Spectrum of **3** irradiation at 4.86 ppm

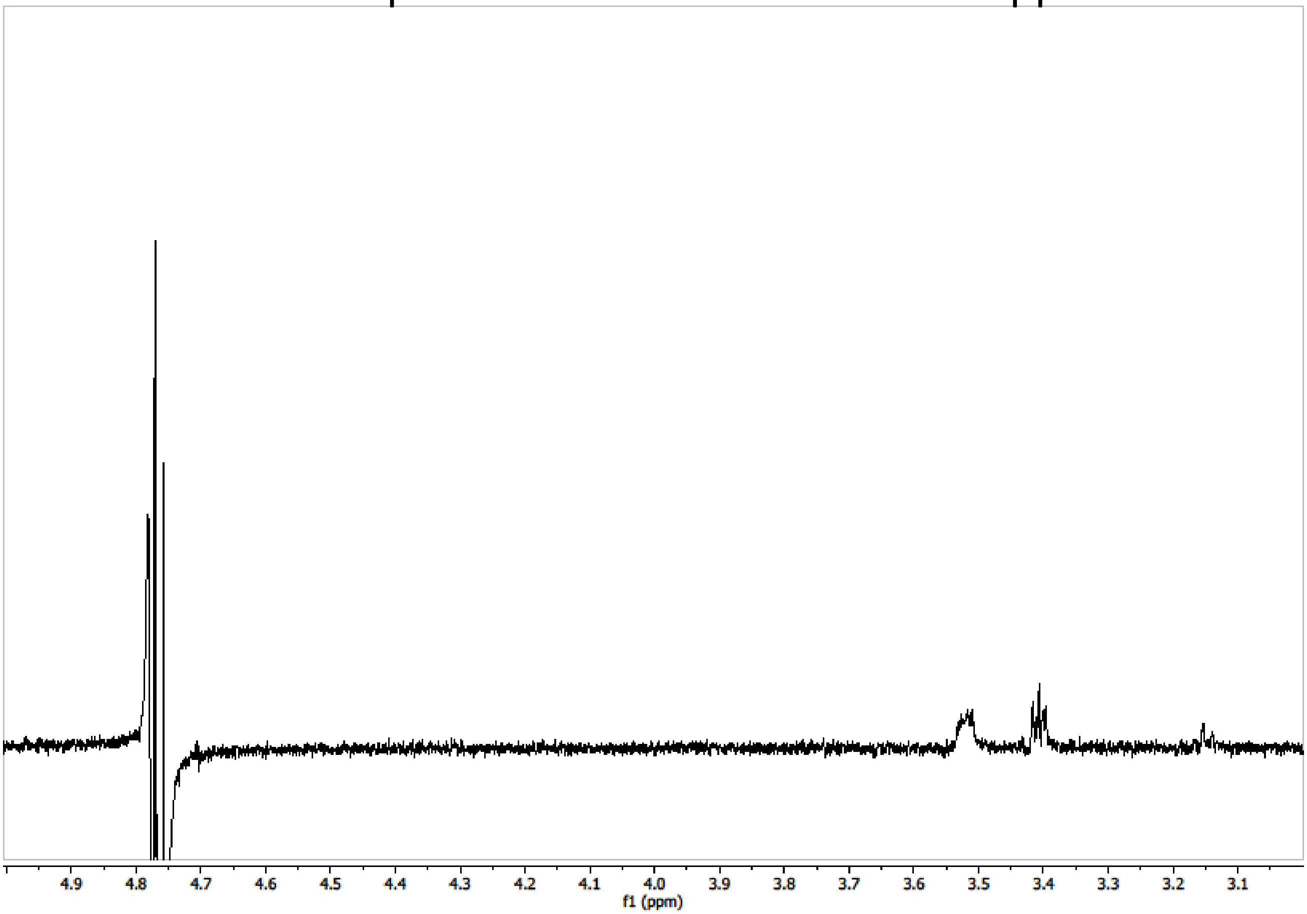

Selective 1D NOESY Spectrum of **3** irradiation at 4.41 ppm

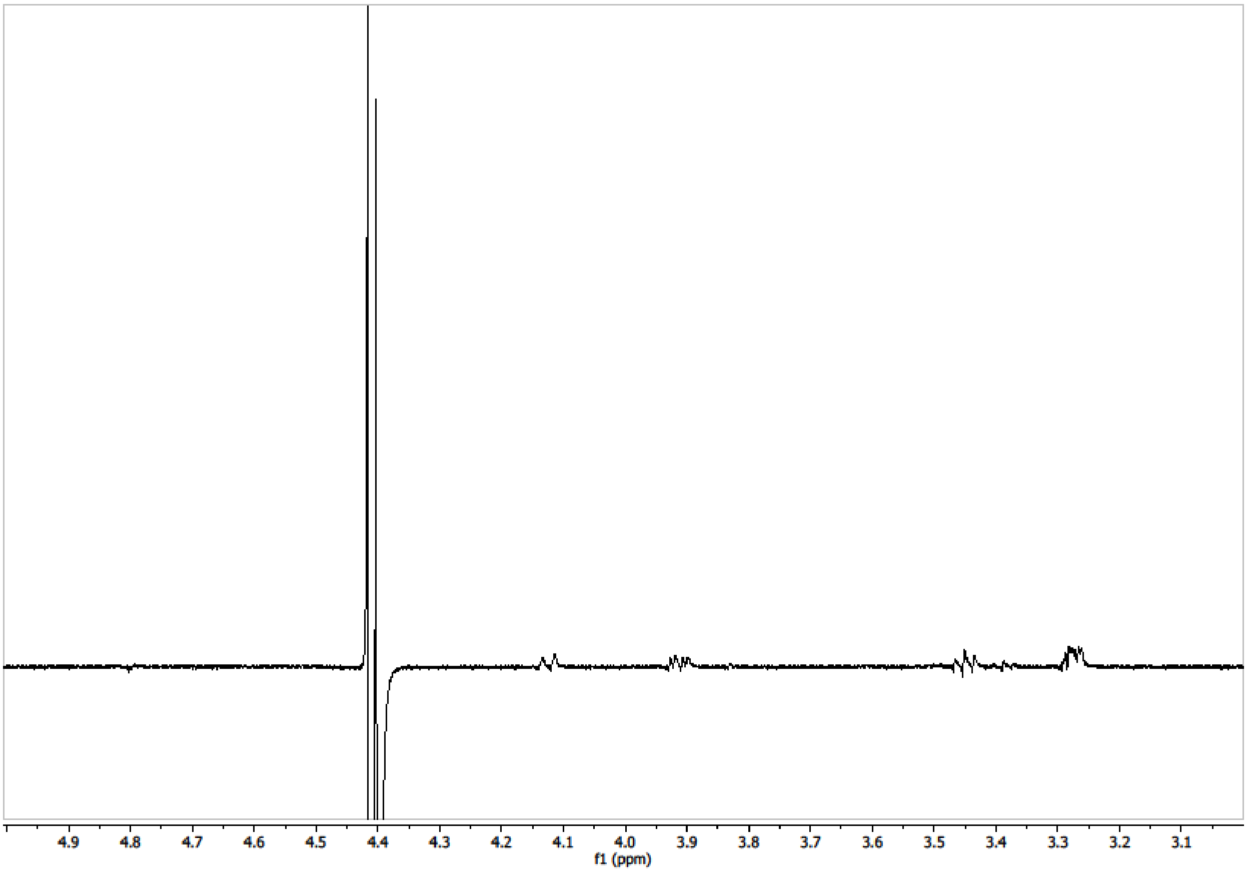

HRMS of **3**

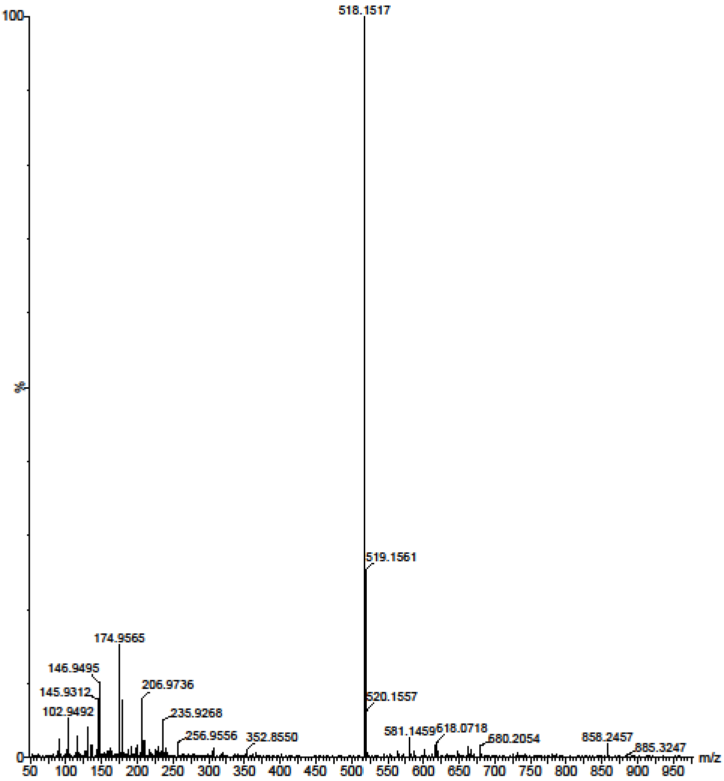

Figure S34 CD Spectrum of **3**

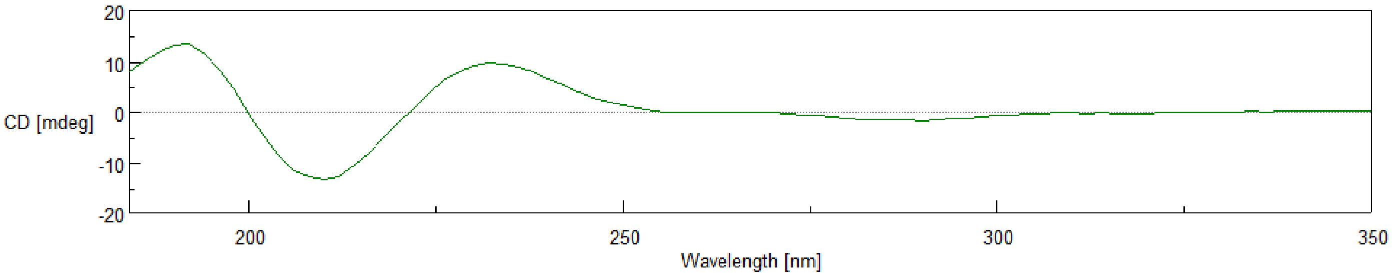

Isolation and structure determination of drought-induced multihexose benzoxazinoids from maize (*Zea mays*)

Supplementary Information SI5:
Structure elucidation of HMBOA-3Glc (4).

Sylvain Sutour[1,*], Cong Van Doan[2,3,4], Pierre Mateo[2], Tobias Züst[5], Ella Raymonde Hartmann[2], Gaétan Glauser[1], Christelle Aurélie Maud Robert[2,3,*]

[1] Neuchâtel Platform of Analytical Chemistry, University of Neuchâtel, 2000 Neuchâtel, Switzerland

[2] Institute of Plant Sciences, University of Bern, 3013 Bern, Switzerland

[3] Oeschger Centre for Climate Change Research (OCCR), University of Bern, 3012 Bern, Switzerland

[4] Plant Physiology Unit, The Department of Life Sciences and Systems Biology of the University of Turin, Via Accademia Albertina 13, 10123 Torino, Italy

[5] Department of Systematic and Evolutionary Botany, University of Zürich, 8008 Zürich, Switzerland

For correspondence:Sutour Sylvain - sylvain.sutour@unine.ch; Christelle A. M. Robert - christelle.robert@unibe.ch

Chemical data for **4**: ECD (c 0.8 mg/mL, H_2O) λmax (Δε) 196 (4.6), 210 (-4.2), 226 (7.6), 288 (-2.7); ^{1}H NMR (D_2O, 600 MHz) δ 7.02 (1H, d, J = 8.8 Hz, H-5), 6.86 (1H, dd, J = 2.6, H-8), 6.78 (1H, d, J = 8.8, 2.6, H-6), 5.84 (1H, s, H-2), 4.86 (1H, d, J = 7.9, H-1'), 4.49 (1H, d, J = 7.9, H-1'''), 4.35 (1H, d, J = 7.9, H-1''), 4.15 (1H, dd, J = 11.8, 2.0, H-6''b), 4.12 (1H, dd, J = 11.0, 2.0, H-6'b), 3.96 (1H, dd, J = 11.0, 5.6, H-6'a), 3.90 (1H, dd, J = 11.9, 1.8, H-6'''b), 3.84 (3H, s, H-11), 3.82 (1H, dd, J = 11.8, 5.3, H-6''a), 3.72 (1H, dd, J = 11.9, 5.5, H-6'''a), 3.61 (1H, ddd, J = 9.3, 5.6, 2.0, H-5'), 3.50 (1H, m, H-3'), 3.49 (1H, m, H-4'), 3.49 (1H, dd, J = 9.3, 9.1, H-3'''), 3.48 (1H, m, H-4''), 3.43 (1H, m, H-3''), 3.43 (1H, ddd, J = 8.8, 5.5, 1.8, H-5'''), 3.39 (1H, dd, J = 9.3, 8.8, H-4'''), 3.32 (1H, m, H-5''), 3.31 (1H, dd, J = 9.3, 7.9, H-2'''), 3.28 (1H, dd, J = 8.7, 7.9, H-2''), 3.27 (1H, dd, J = 9.3, 7.9, H-2'); ^{13}C NMR (D2O, 151 MHz) δ 163.7 (C, C-3), 156.4 (C, C-7), 141.1 (C, C-9), 118.4 (C, C-10), 117.1 (CH, C-5), 109.2 (CH, C-6), 104.2 (CH, C-8), 103.5 (CH, C-1''), 102.7 (CH, C-1'''), 102.2 (CH, C-1'), 95.5 (CH, C-2), 75.7 (CH, C-3''), 75.6 (CH, C-3'''), 75.5 (CH, C-5'''), 75.2 (CH, C-3'), 75.2 (CH, C-5'), 73.1 (CH, C-2''), 73.0 (CH, C-5''), 73.0 (CH, C-2'''), 72.6 (CH, C-2'), 69.4 (CH, C-4'''), 69.2 (CH, C-4''), 69.0 (CH2, C-6'), 68.5 (CH, C-4'), 68.2 (CH2, C-6''), 60.5 (CH2, C-6'''), 55.9 (CH3, C-11). HRESIMS m/z 680.2031 (calcd for C27H38NO19, 680.2038).

^1H NMR Spectrum of **4** (D_2O, 600 MHz)

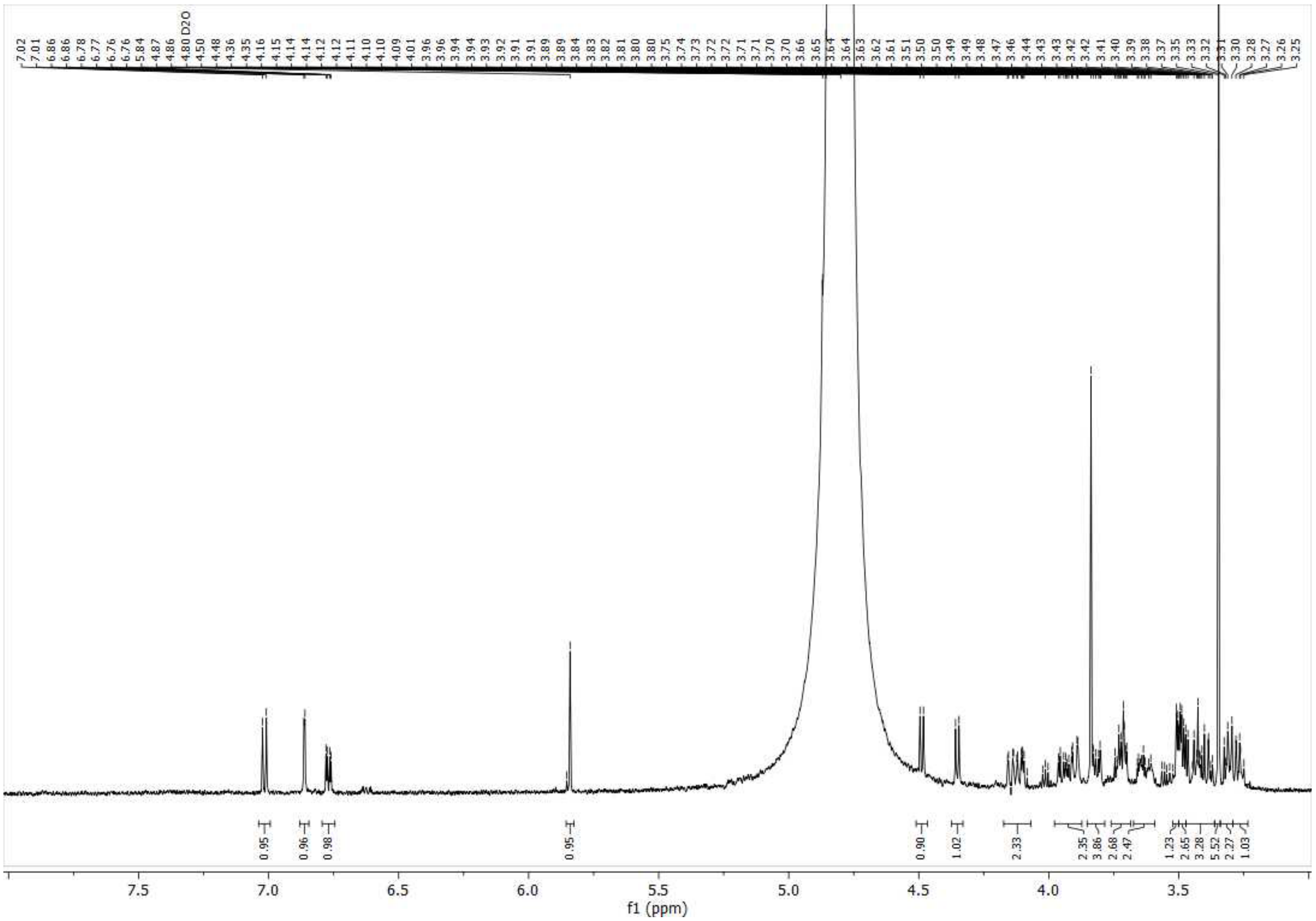

HSQC Spectrum of **4**

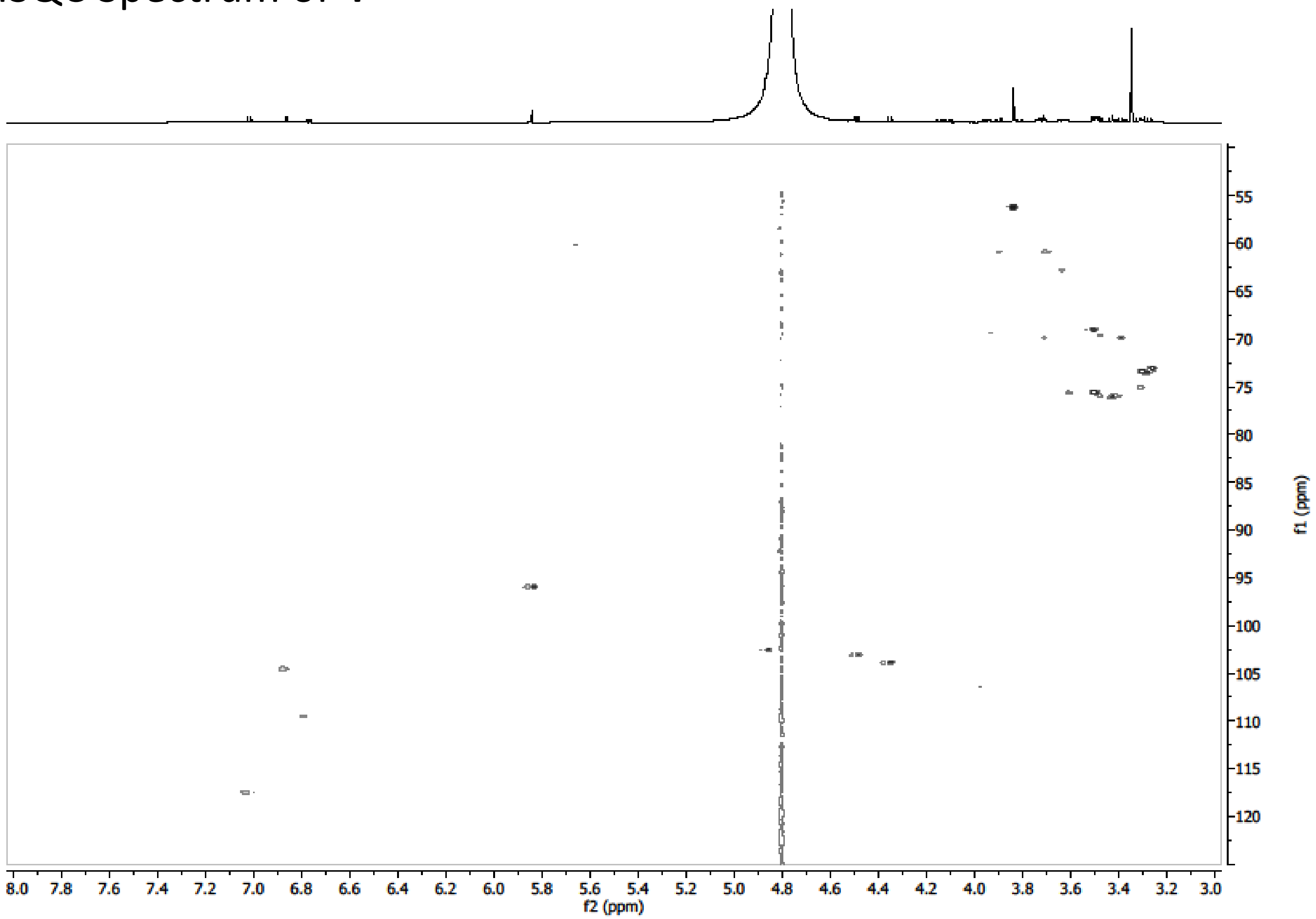

Band selective HSQC Spectrum of **4**

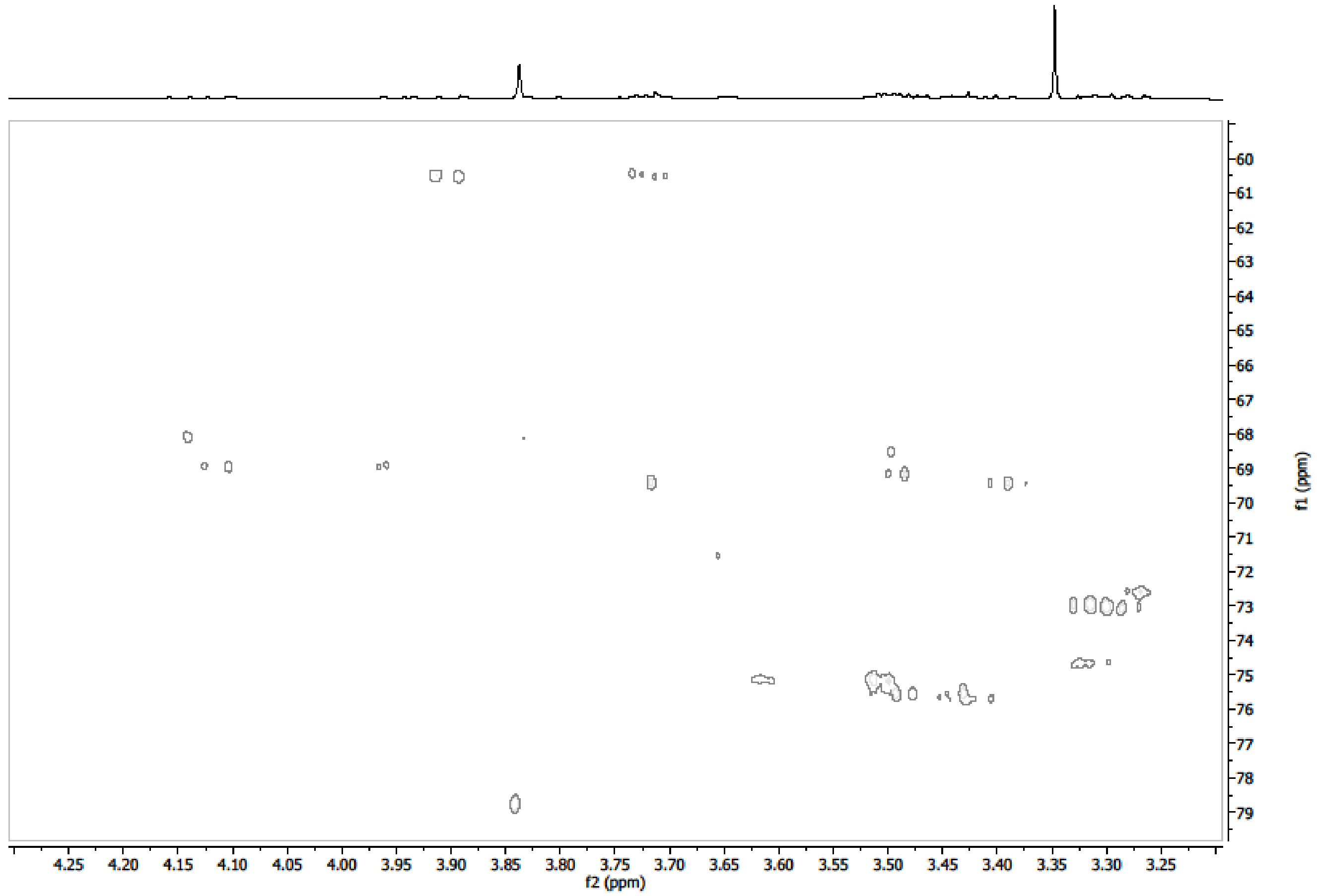

HMBC Spectrum of **4**

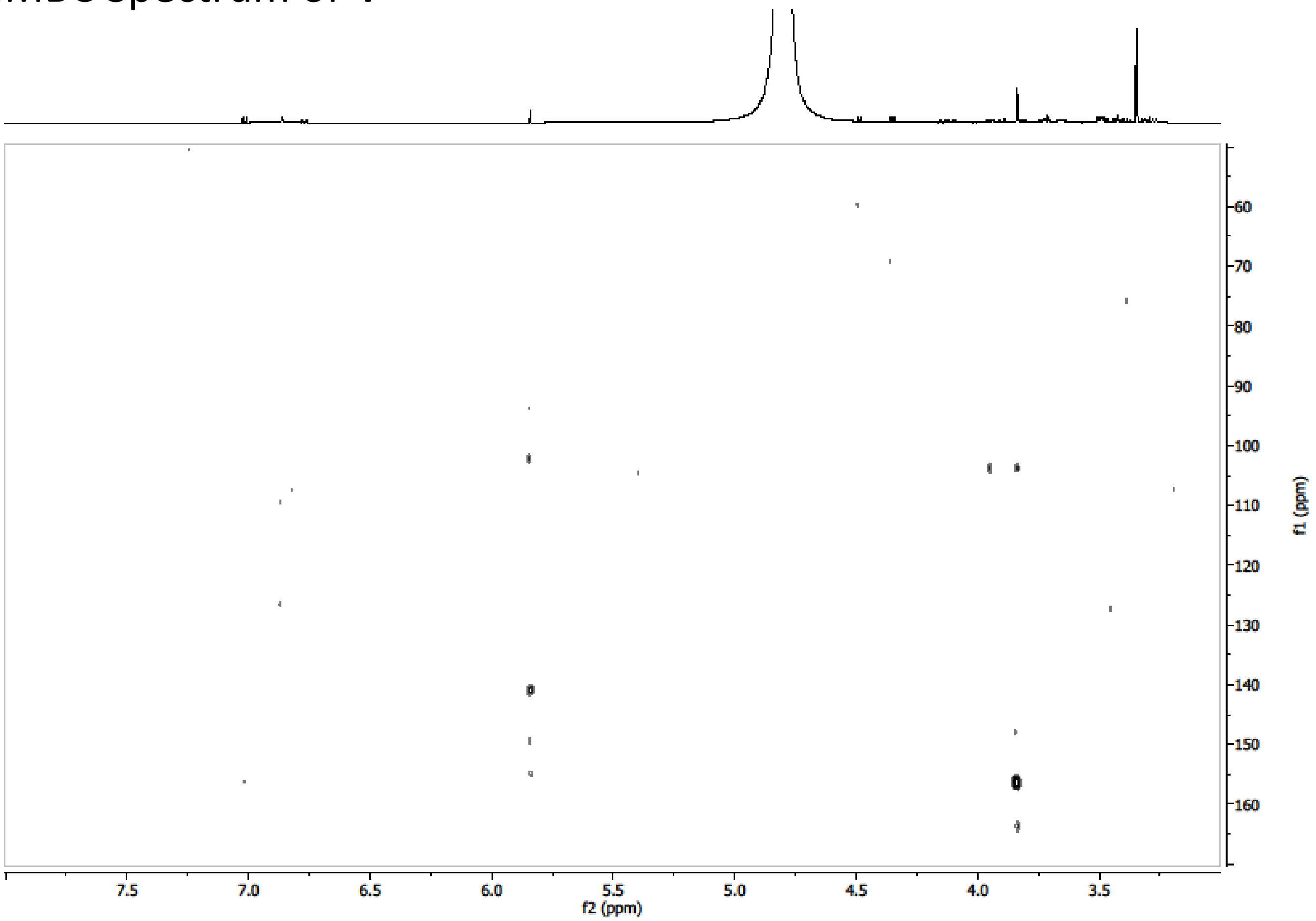

CLIP HSQC Spectrum of **4**

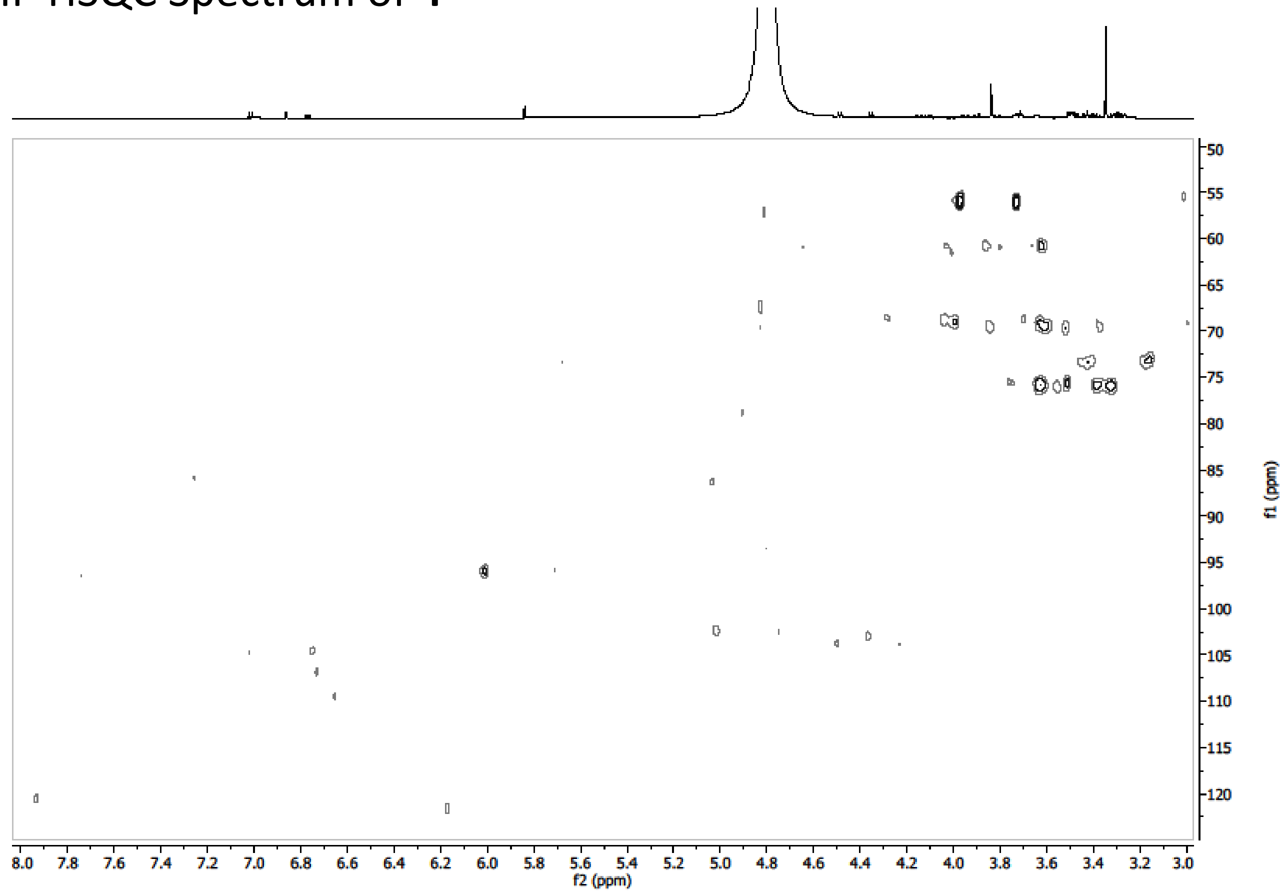

Selective 1D TOCSY Spectrum of **4** irradiation at 4.86 ppm

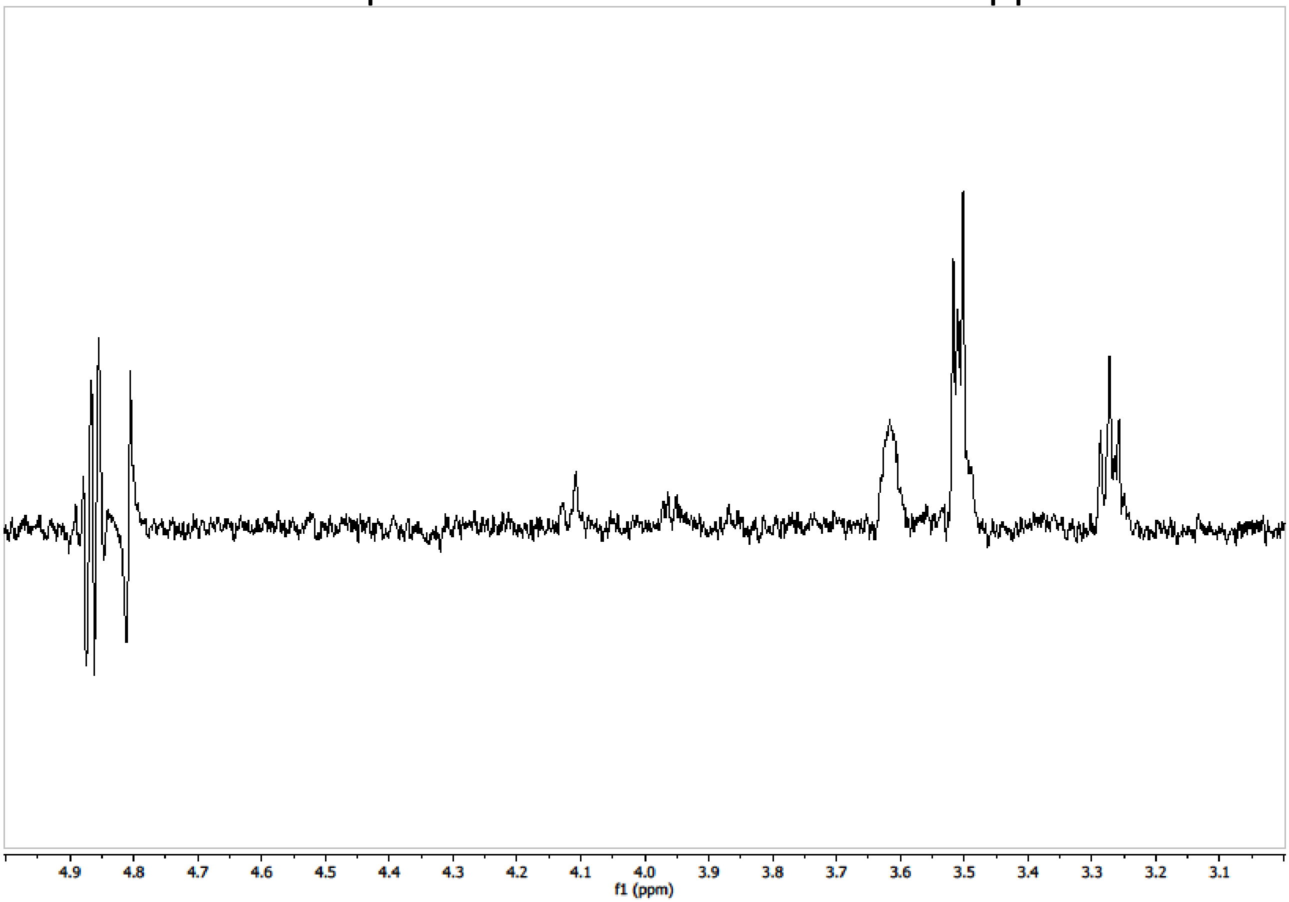

Selective 1D TOCSY Spectrum of **4** irradiation at 4.49 ppm

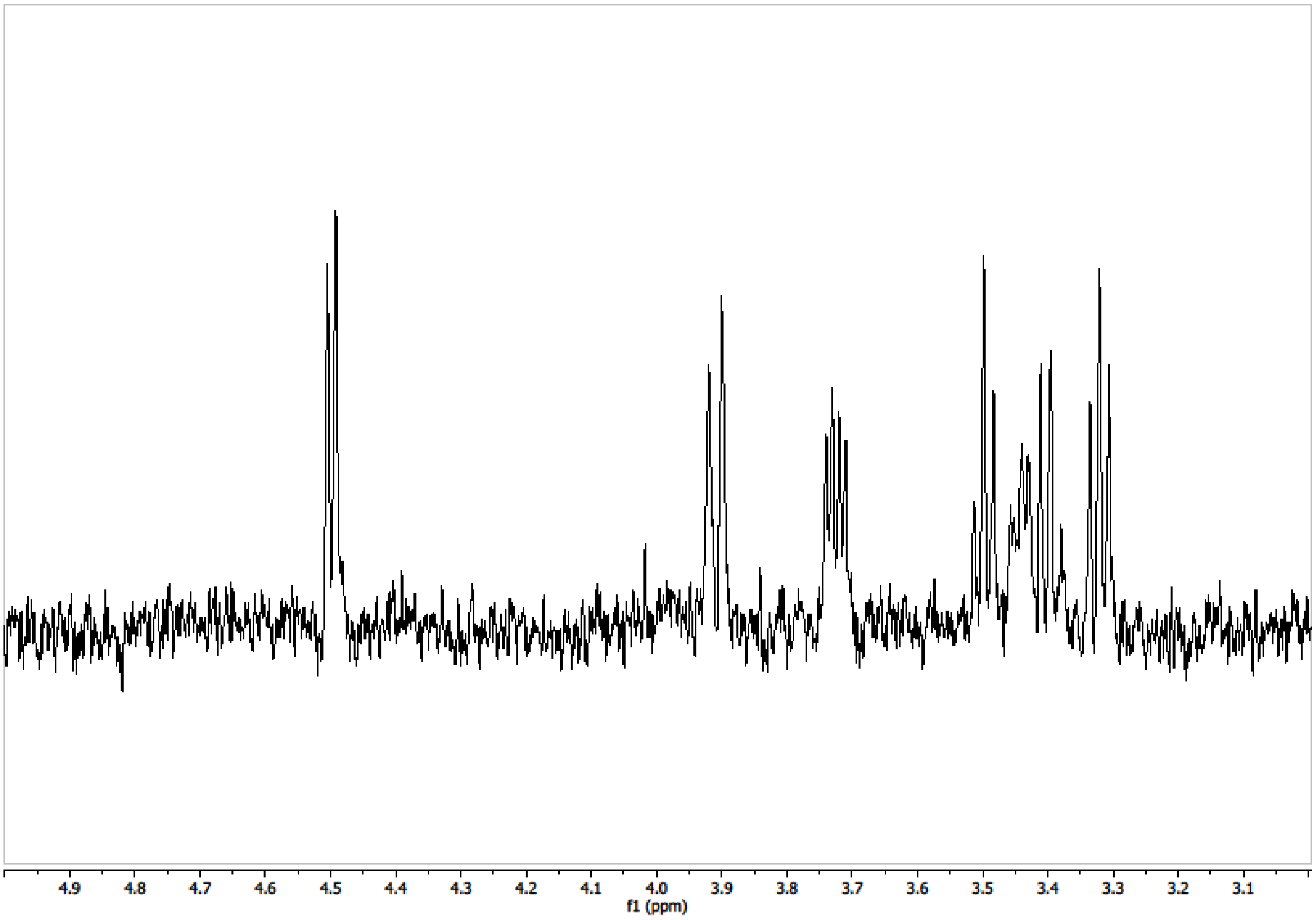

Selective 1D NOESY Spectrum of **4** irradiation at 4.35 ppm

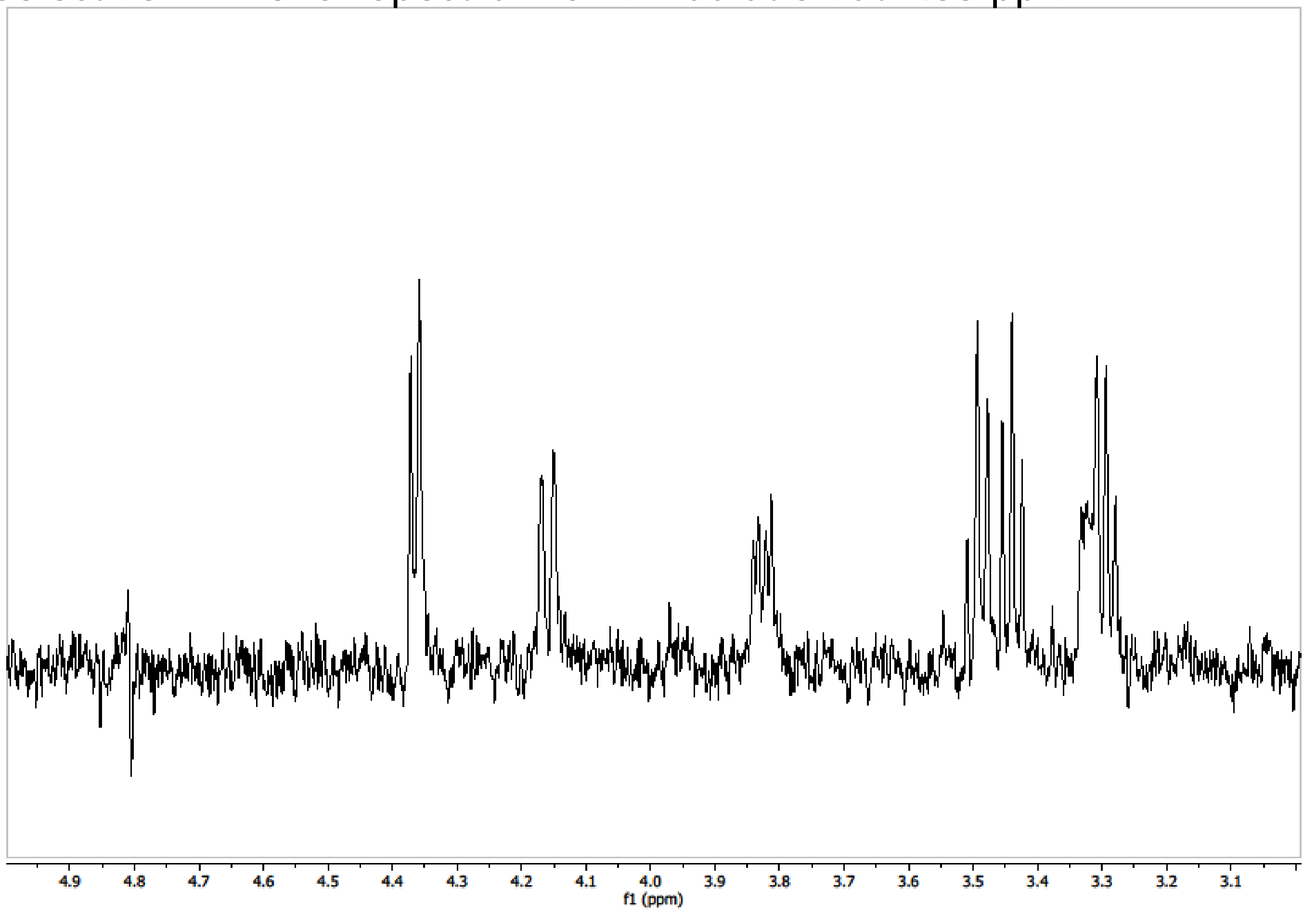

HRMS of **4**

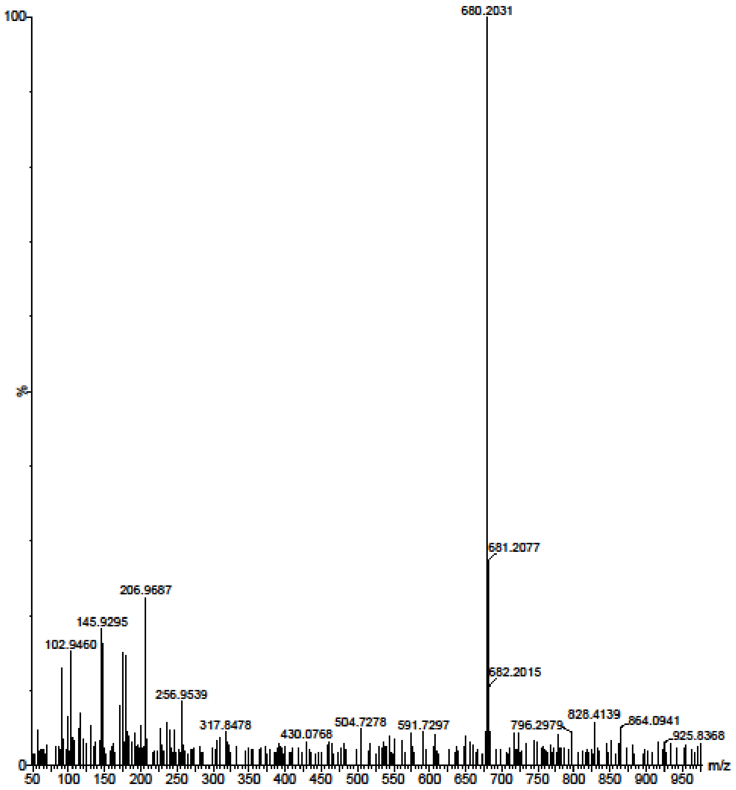

CD Spectrum of **4**

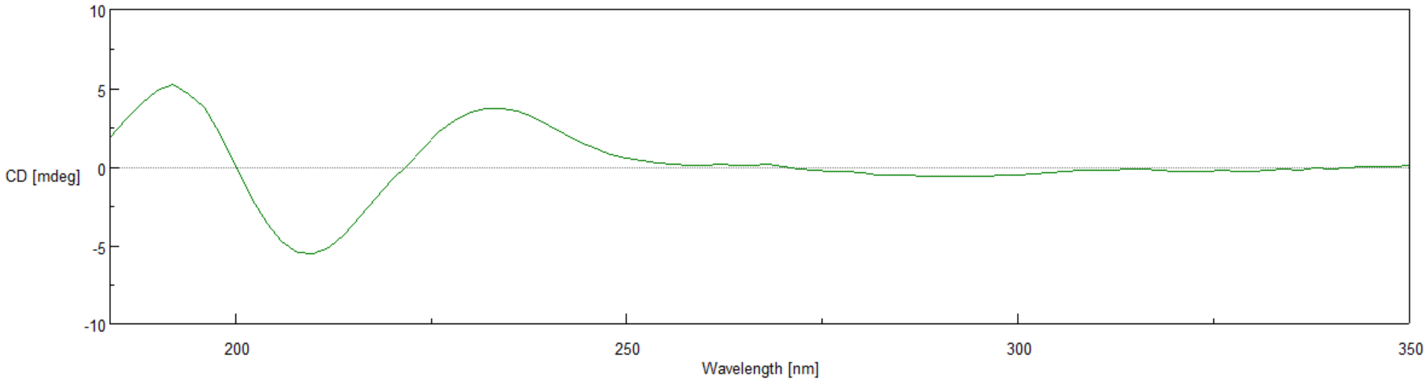

Isolation and structure determination of drought-induced multihexose benzoxazinoids from maize (*Zea mays*)

Supplementary Information SI6:

Structure elucidation of HM_2BOA-2Glc (5).

Sylvain Sutour[1,*], Cong Van Doan[2,3,4], Pierre Mateo[2], Tobias Züst[5], Ella Raymonde Hartmann[2], Gaétan Glauser[1], Christelle Aurélie Maud Robert[2,3,*]

[1] Neuchâtel Platform of Analytical Chemistry, University of Neuchâtel, 2000 Neuchâtel, Switzerland

[2] Institute of Plant Sciences, University of Bern, 3013 Bern, Switzerland

[3] Oeschger Centre for Climate Change Research (OCCR), University of Bern, 3012 Bern, Switzerland

[4] Plant Physiology Unit, The Department of Life Sciences and Systems Biology of the University of Turin, Via Accademia Albertina 13, 10123 Torino, Italy

[5] Department of Systematic and Evolutionary Botany, University of Zürich, 8008 Zürich, Switzerland

For correspondence:Sutour Sylvain - sylvain.sutour@unine.ch; Christelle A. M. Robert - christelle.robert@unibe.ch

Chemical data for **5**: ECD (c 0.9 mg/mL, H2O) λmax (Δε) 196 (16.2), 226 (-8.5), 258 (5.9), 282 (-2.2); ^{1}H NMR (D_2O, 600 MHz) δ 7.19 (1H, d, J = 8.8 Hz, H-5), 6.91 (1H, d, J = 8.8 Hz, H-6), 6.07 (1H, s, H-2), 4.88 (1H, d, J = 7.8 Hz, H-1'), 4.39 (1H, d, J = 7.9 Hz, H-1''), 4.07 (1H, dd, J = 12.0, 1.6 Hz, H-6'b), 3.93 (1H, dd, J = 12.0, 4.2 Hz, H-6'a), 3.92 (3H, s, OMe H-12), 3.89 (3H, s, OMe H-11), 3.84 (1H, dd, J = 12.3, 2.2 Hz, H-6''b), 3.69 (1H, dd, J = 12.3, 5.5 Hz, H-6''a), 3.60 (1H, ddd, J = 7.3, 4.2, 1.6 Hz, H-5'), 3.52 (1H, dd, J = 8.5, 7.2 Hz, H-3'), 3.51 (1H, td, J = 7.3, 2.2 Hz, H-4'), 3.42 (1H, t, J = 9.2, Hz, H-3''), 3.36 (1H, dd, J = 9.5, 9.2 Hz, H-4''), 3.25 (1H, ddd, J = 8.5, 7.8, 2.2 Hz, H-2'), 3.23 (1H, dd, J = 9.0, 7.9 Hz, H- 2''), 3.23 (1H, ddd, J = 9.5, 5.5, 2.2 Hz, H-5''); ^{13}C NMR (D2O, 151 MHz) δ 156.0 (C, C-3), 149.8 (C, C-7), 137.2 (C, C-8), 134.6 (C, C-9), 122.2 (CH, C-10), 108.7 (CH, C-5), 107.0 (CH, C-6), 103.2 (CH, C-1''), 102.3 (CH, C-1'), 97.2 (CH, C-2), 78.0 (CH, C-5''), 77.9 (CH, C-3''), 77.5 (CH, C-5'), 75.2 (CH, C-2''), 75.1 (CH, C-3'), 73.1 (CH, C-2'), 71.6 (CH, C-4''), 68.6 (CH, C-4'), 68.4 (CH2, C-6'), 61.6 (CH3, C-12), 60.5 (CH2, C-6'') , 56.2 (CH3, C-12). HRESIMS m/z 548.1618 (calcd for C22H30NO15, 548.1615).

^{1}H NMR Spectrum of **5** (D_2O, 600 MHz)

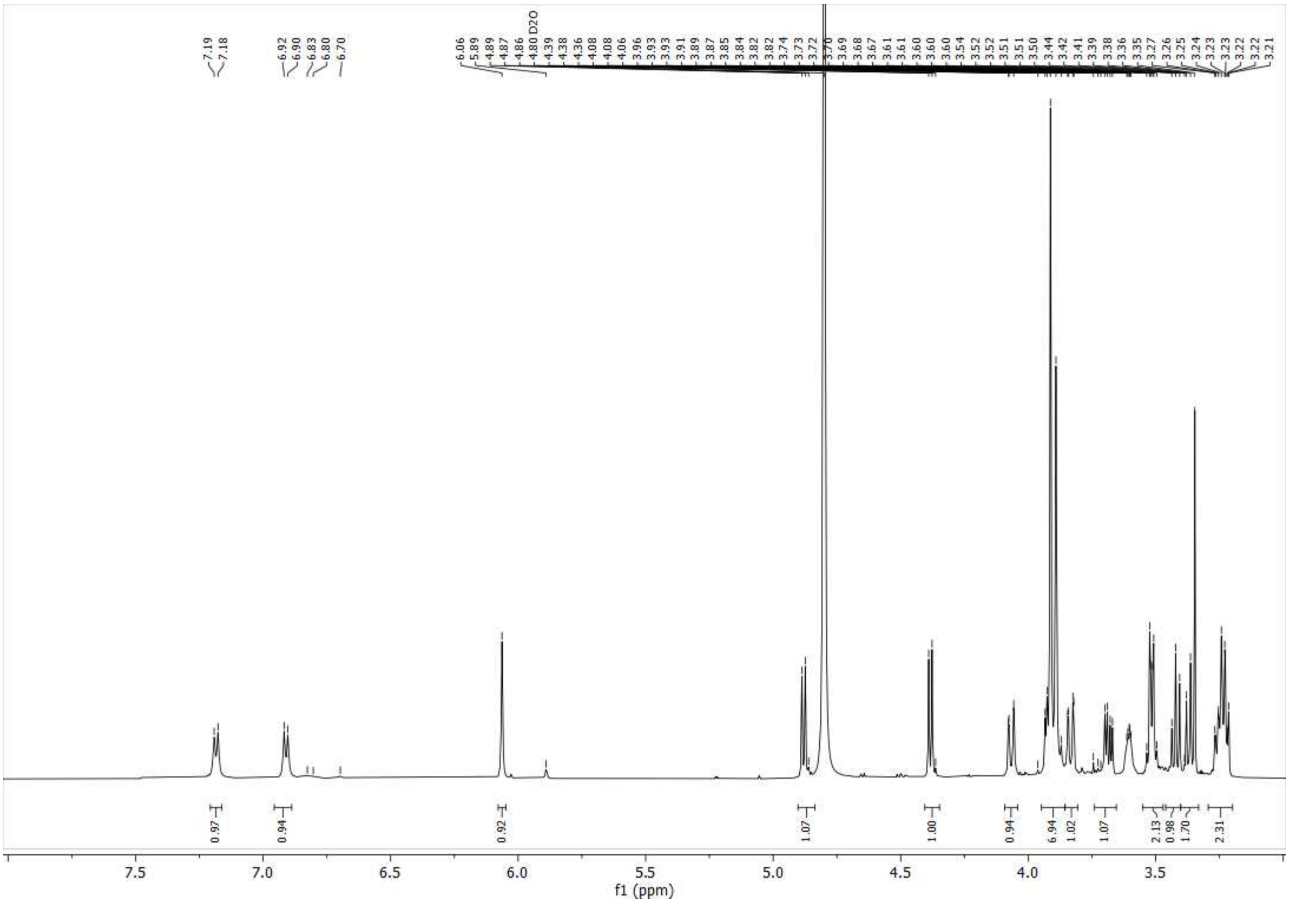

^{13}C NMR Spectrum of **5** (D_2O, 600 MHz)

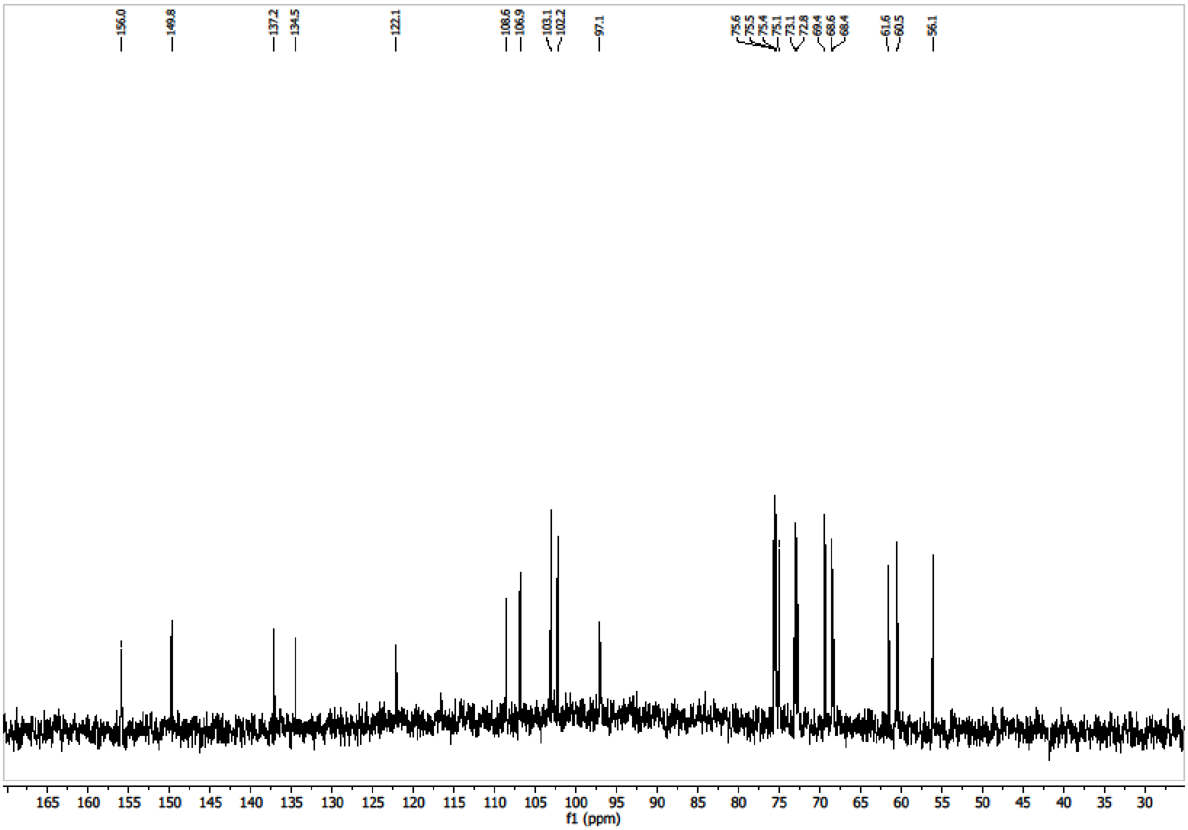

HSQC Spectrum of **5**

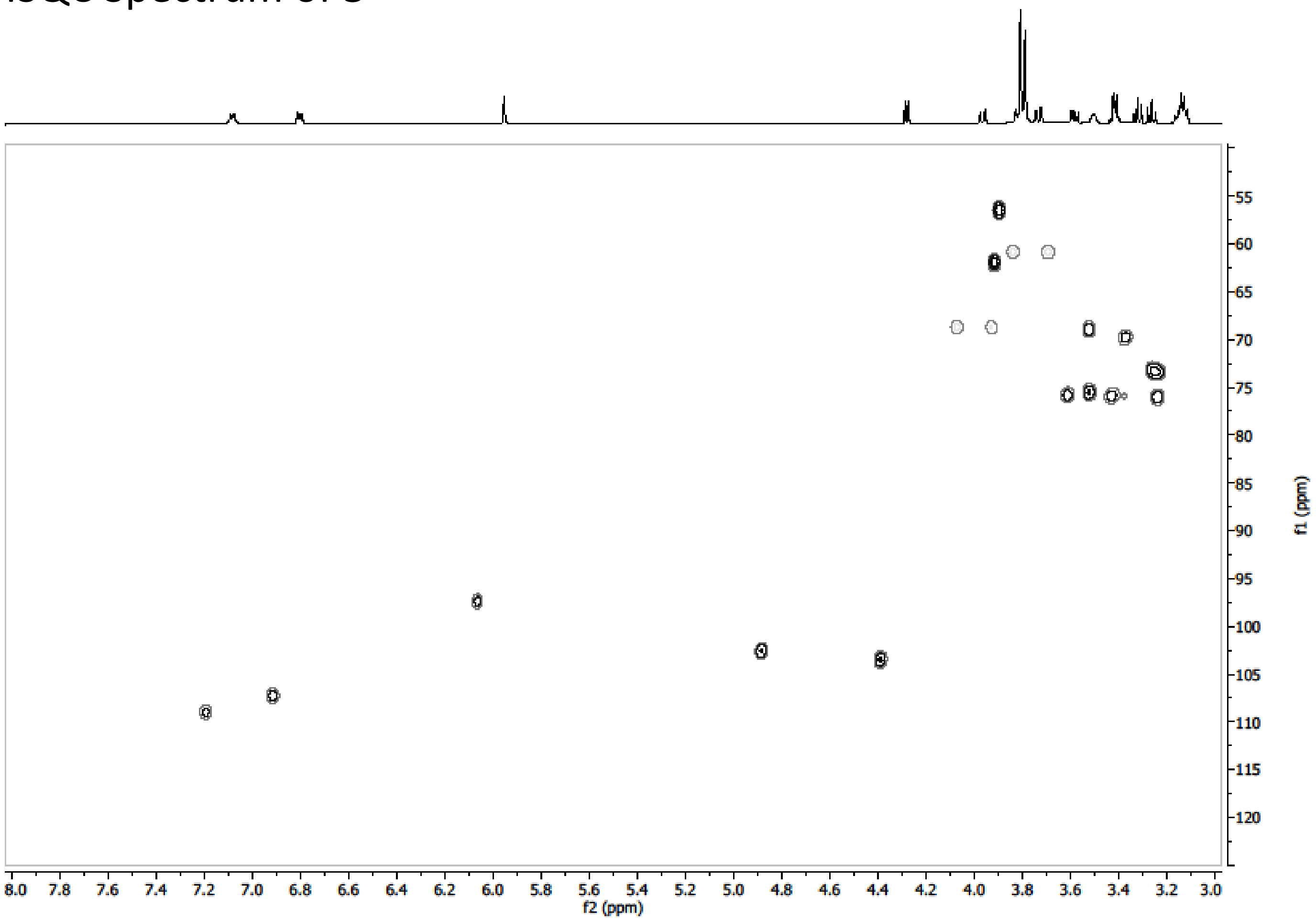

Band selective HSQC Spectrum of **5**

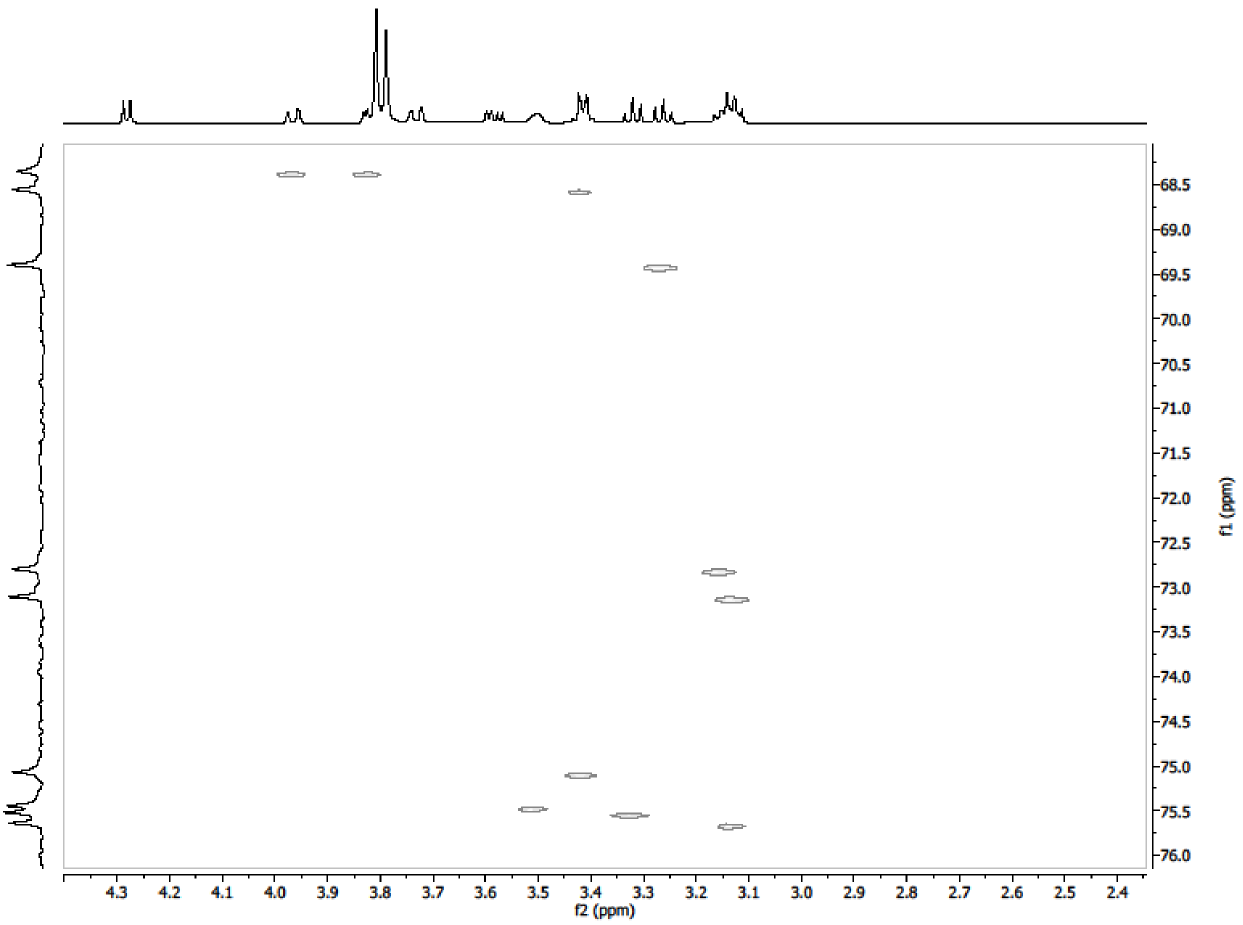

HMBC Spectrum of **5**

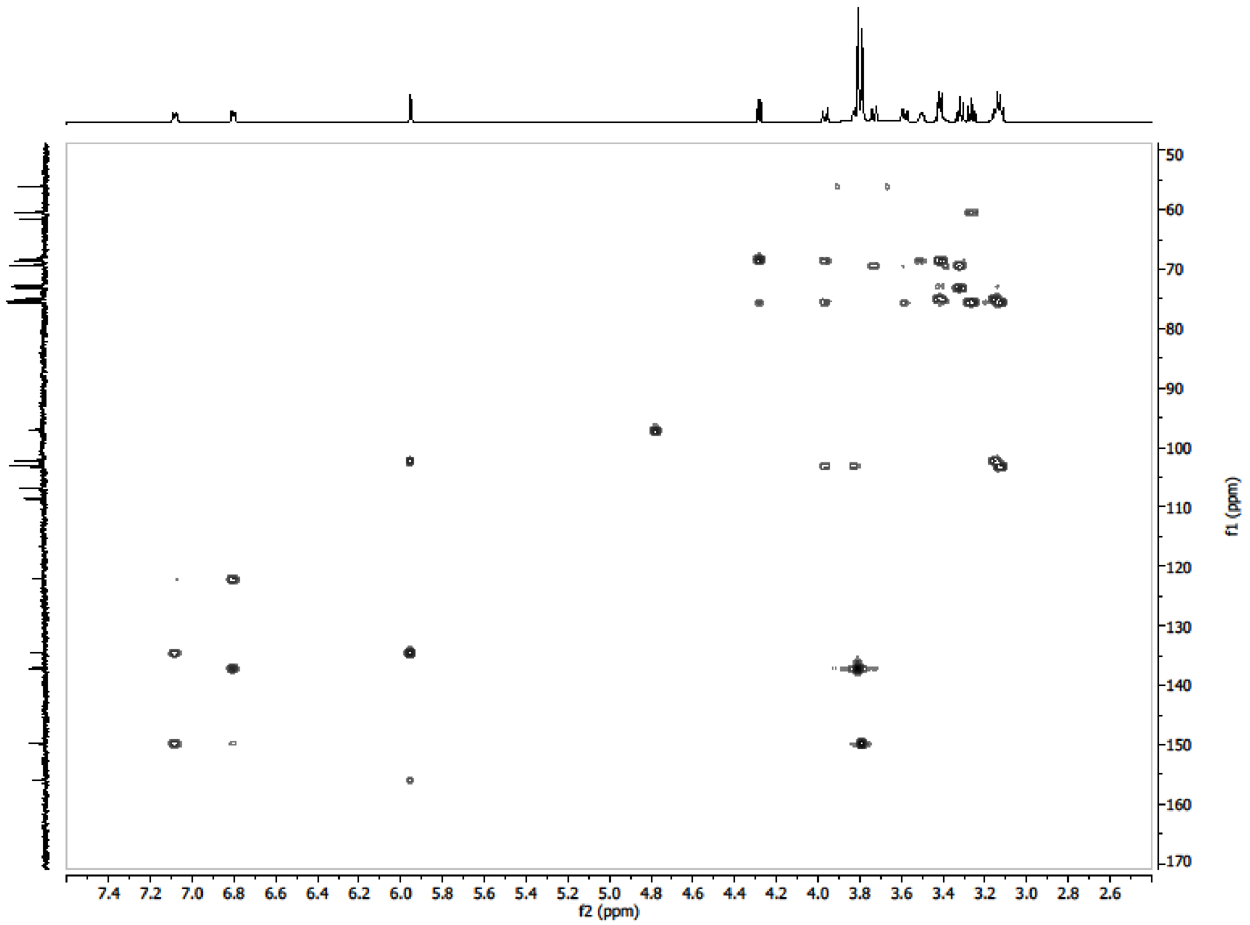

CLIP HSQC Spectrum of **5**

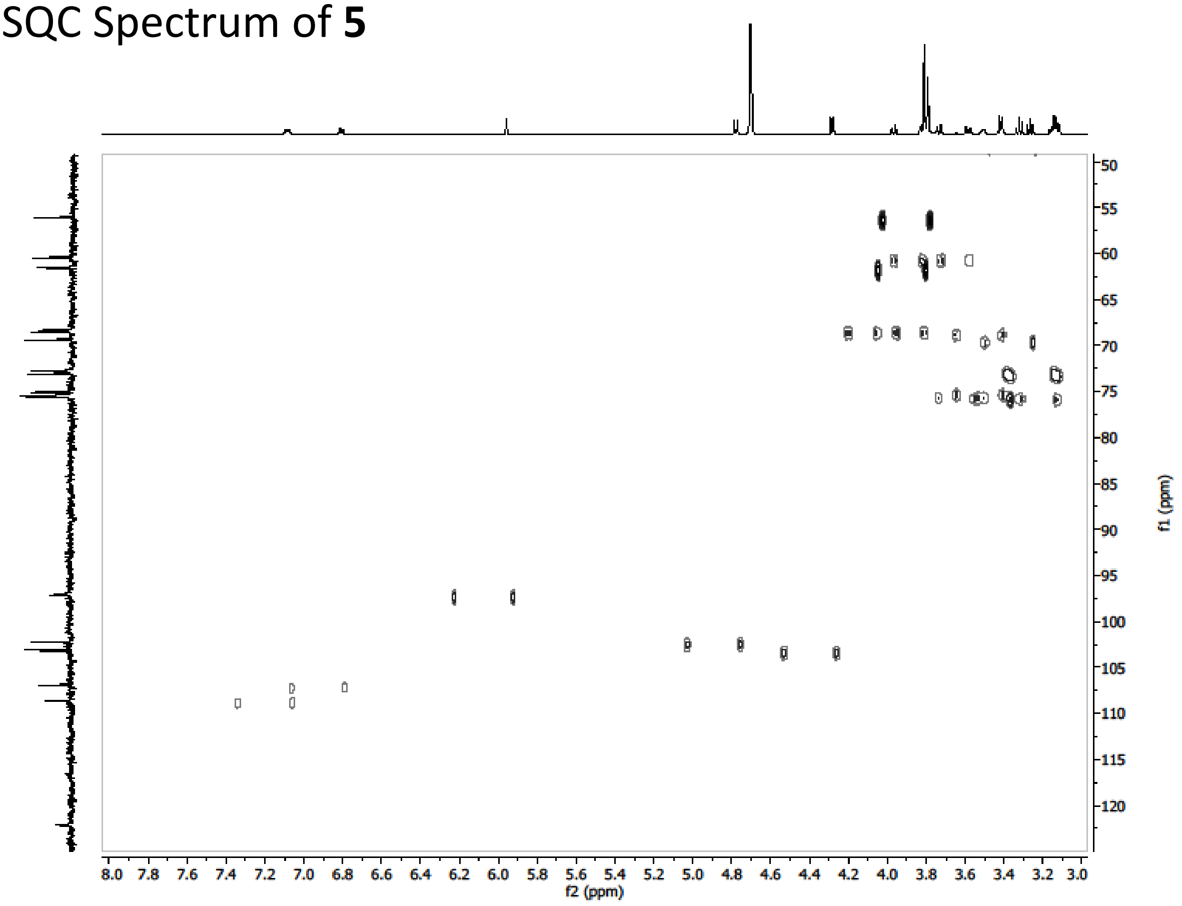

NOESY Spectrum of **5**

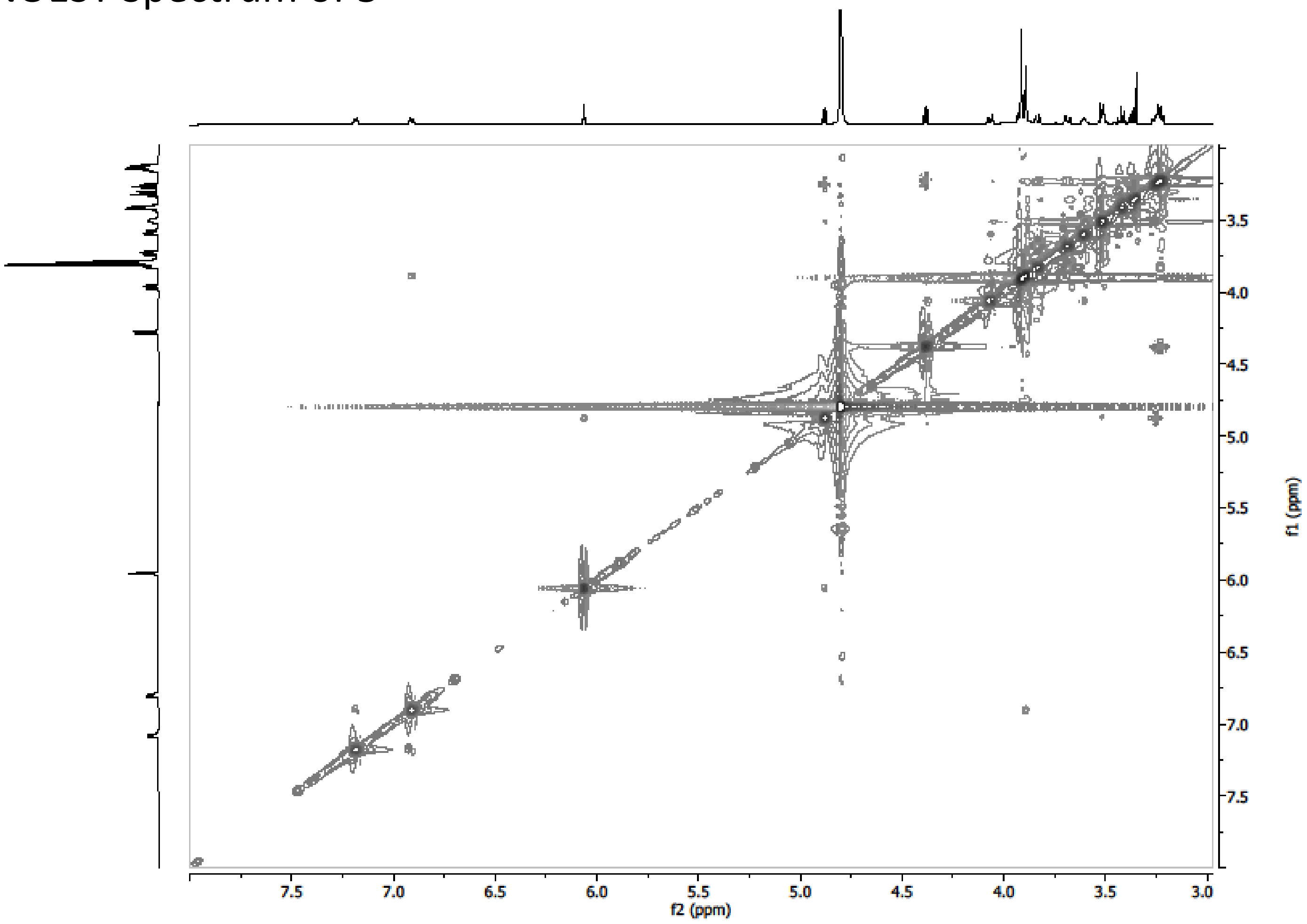

Selective 1D TOCSY Spectrum of **5** irradiation at 4.88 ppm

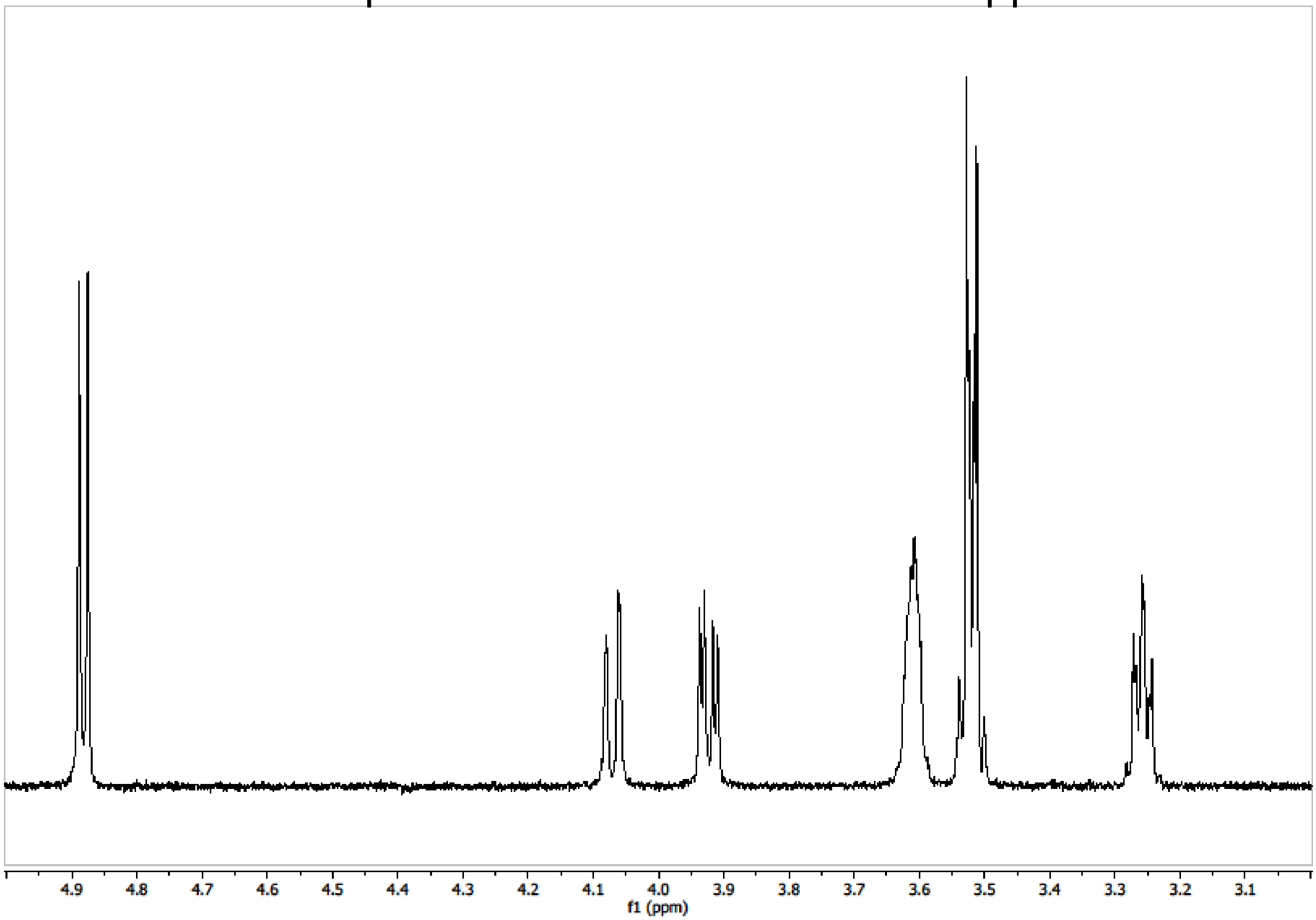

Selective 1D TOCSY Spectrum of **5** irradiation at 4.37 ppm

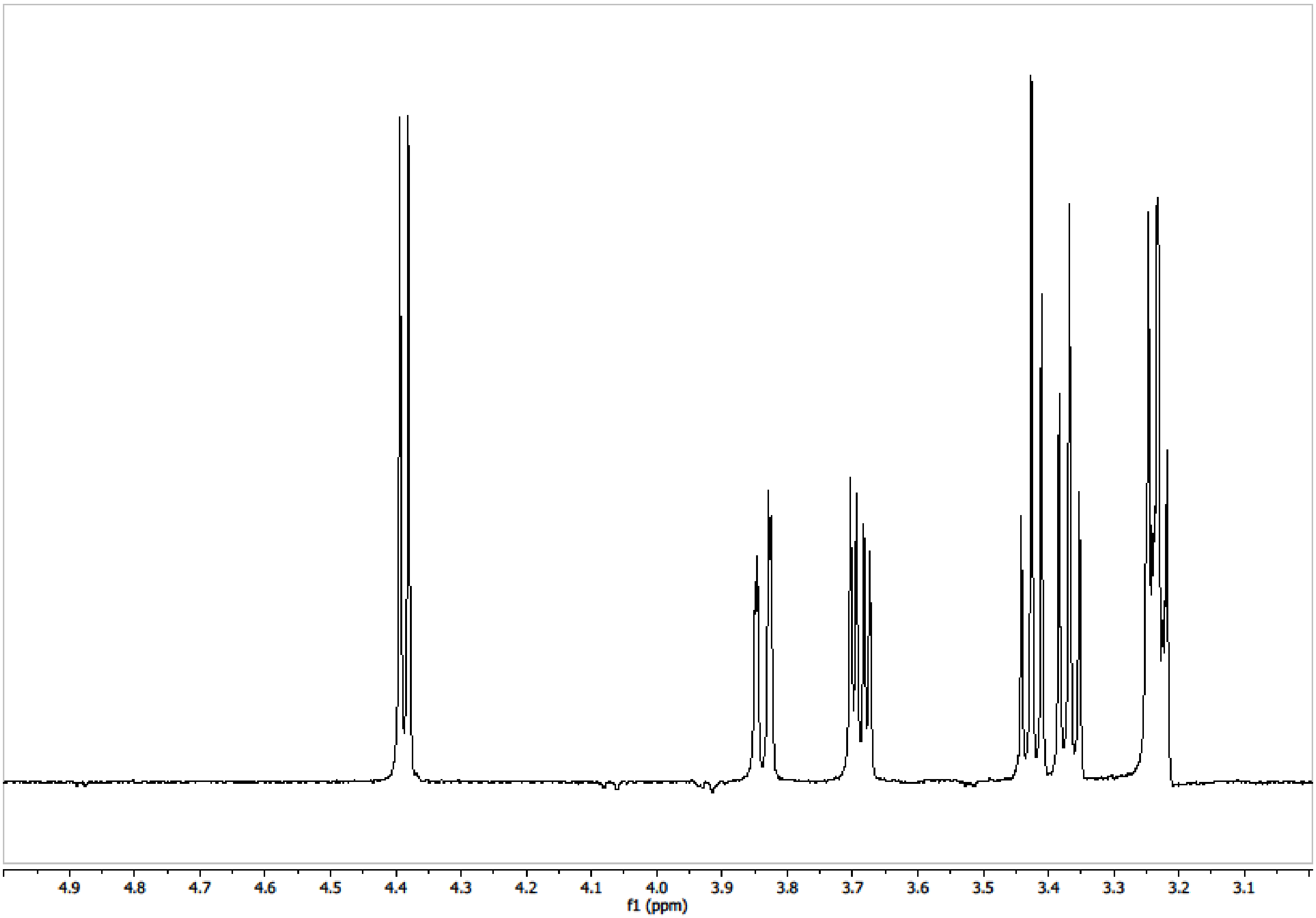

HRMS of **5**

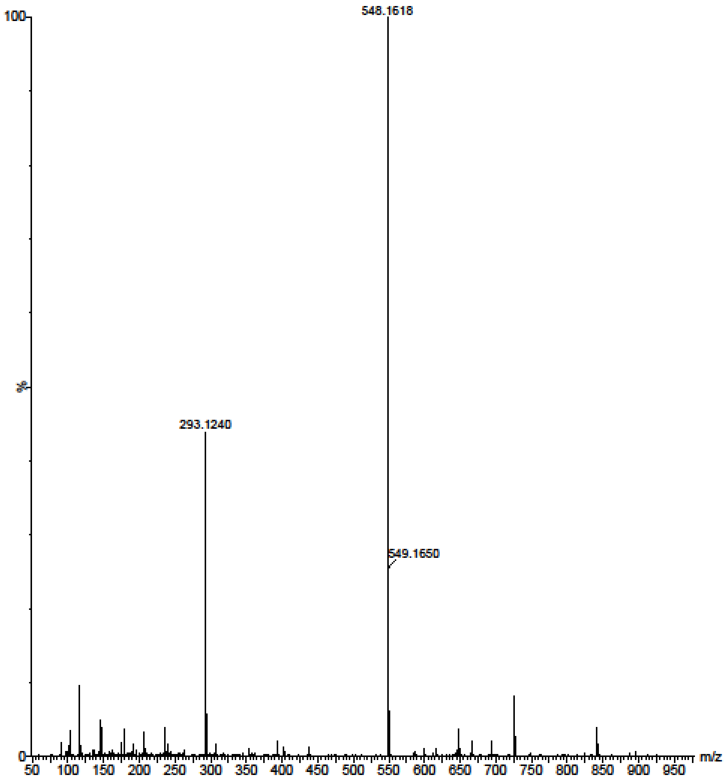

CD Spectrum of **5**

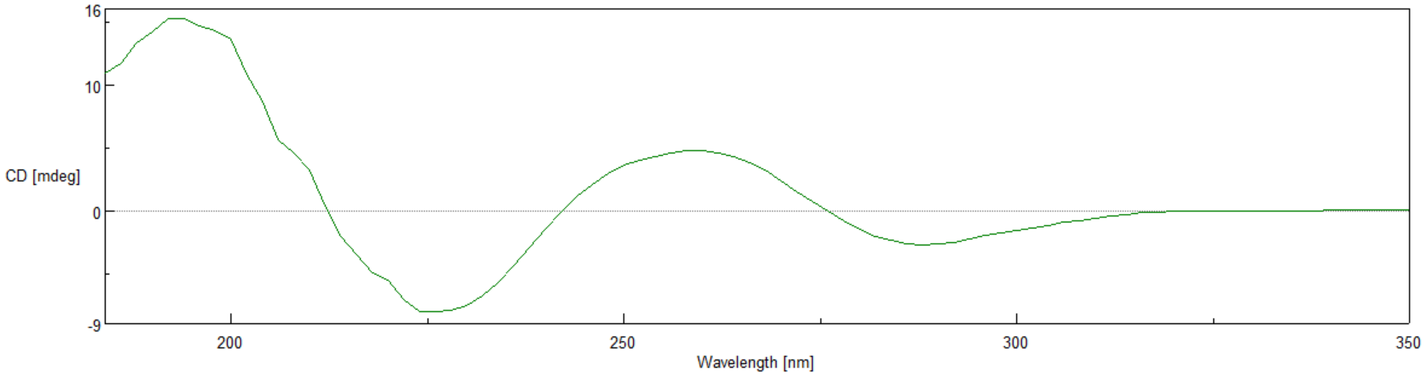

Isolation and structure determination of drought-induced multihexose benzoxazinoids from maize (*Zea mays*)

Supplementary Information SI7:

Structure elucidation of HDMBOA-2Glc (6).

Sylvain Sutour[1,*], Cong Van Doan[2,3,4], Pierre Mateo[2], Tobias Züst[5], Ella Raymonde Hartmann[2], Gaétan Glauser[1], Christelle Aurélie Maud Robert[2,3,*]

[1] Neuchâtel Platform of Analytical Chemistry, University of Neuchâtel, 2000 Neuchâtel, Switzerland

[2] Institute of Plant Sciences, University of Bern, 3013 Bern, Switzerland

[3] Oeschger Centre for Climate Change Research (OCCR), University of Bern, 3012 Bern, Switzerland

[4] Plant Physiology Unit, The Department of Life Sciences and Systems Biology of the University of Turin, Via Accademia Albertina 13, 10123 Torino, Italy

[5] Department of Systematic and Evolutionary Botany, University of Zürich, 8008 Zürich, Switzerland

For correspondence:Sutour Sylvain - sylvain.sutour@unine.ch; Christelle A. M. Robert - christelle.robert@unibe.ch

Chemical data for **6**: ECD (c 0.5 mg/mL, H2O) λmax (Δε) 192 (4.7), 208 (-1.6), 234 (3.4), 288 (-0.7); ^{1}H NMR (D_2O, 600 MHz) δ 7.36 (1H, d, J = 8.9 Hz, H-5), 6.92 (1H, d, J = 2.5 Hz, H-8), 6.88 (1H, dd, J = 8.9, 2.5 Hz, H-6), 6.02 (1H, s, H-2), 4.88 (1H, d, J = 8.2 Hz, H-1'), 4.41 (1H, d, J = 7.9 Hz, H-1''), 4.14 (1H, dd, J = 12.1, 1.4 Hz, H-6'b), 3.98 (3H, s, N-OMe H-12), 3.93 (1H, dd, J = 12.1, 4.2 Hz, H-6'a), 3.87 (3H, s, OMe H-11), 3.89 (1H, dd, J = 12.4, 2.1 Hz, H-6''b), 3.73 (1H, dd, J = 12.4, 5.6 Hz, H-6''a), 3.63 (1H, ddd, J = 7.6, 5.6, 2.0 Hz, H-5'), 3.50 (1H, m, H-3'), 3.49 (1H, m, H-4'), 3.46 (1H, m, H-3''), 3.40 (1H, m, H-4''), 3.29 (1H, dd, J = 9.1, 7.8 Hz, H-2''), 3.28 (1H, m, H- 5''), 3.25 (1H, dd, J = 8.1, 7.9 Hz, H-2'); ^{13}C NMR (D_2O, 151 MHz) δ 157.6 (C, C-7), 156.4 (C, C-3), 142.0 (C, C-9), 118.9 (C, C-10), 114.8 (CH, C-5), 109.8 (CH, C-6), 105.0 (CH, C-8), 103.7 (CH, C-1''), 102.6 (CH, C-1'), 97.8 (CH, C-2), 76.3 (CH, C-5''), 76.0 (CH, C-3''), 75.8 (CH, C-5'), 75.7 (CH, C-3'), 73.6 (CH, C-2''), 73.1 (CH, C-2'), 70.0 (CH, C-4''), 69.1 (CH, C-4'), 69.1 (CH2, C-6'), 63.6 (CH3, C-12), 61.1 (CH2, C-6''), 56.3 (CH3, C-11). HRESIMS m/z 594.1670 (formate adduct, calcd for C23H32NO17, 594.1670).

^{1}H NMR Spectrum of **6** (D_2O, 600 MHz)

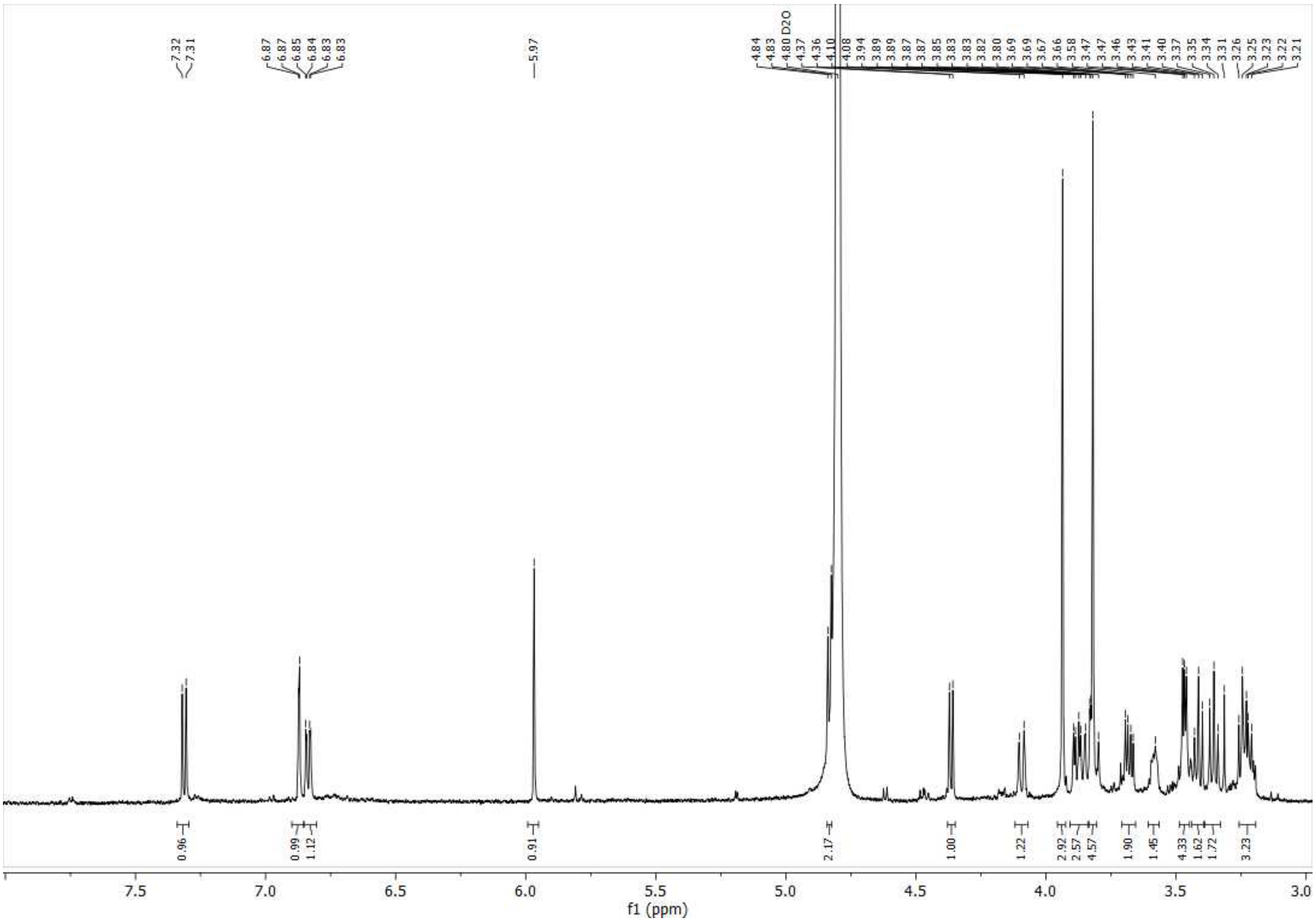

HSQC Spectrum of **6**

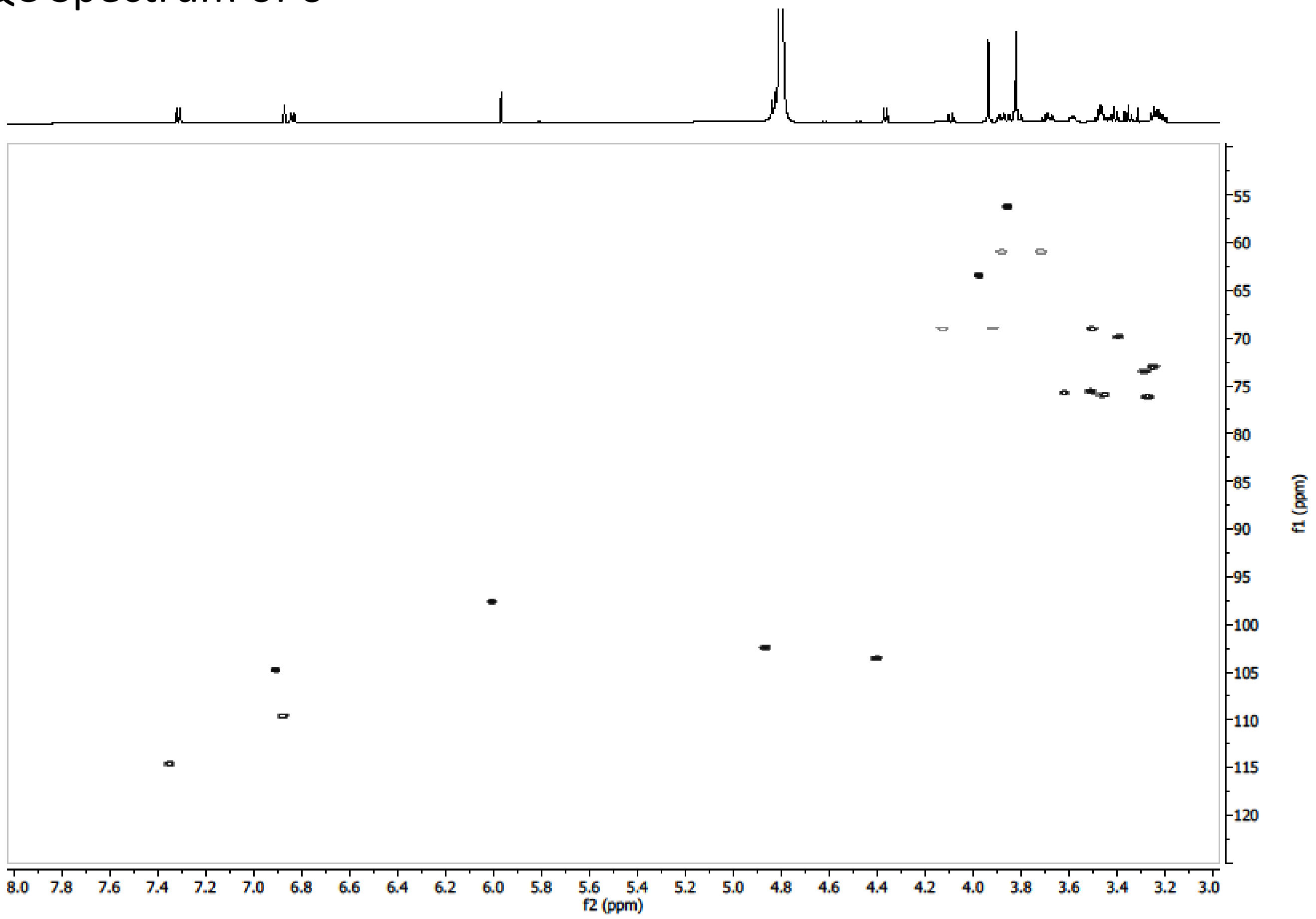

HMBC Spectrum of **6**

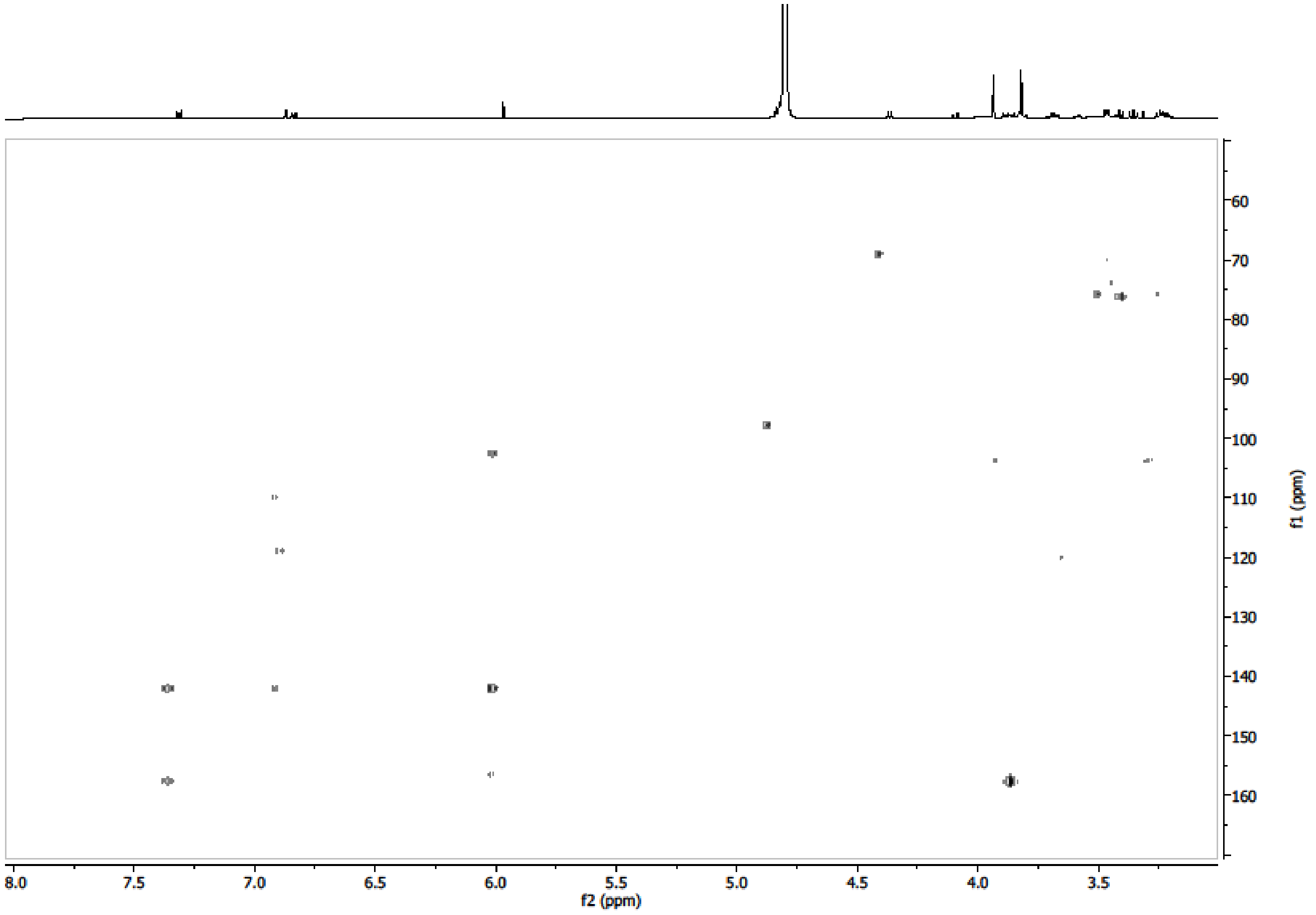

CLIP HSQC Spectrum of **6**

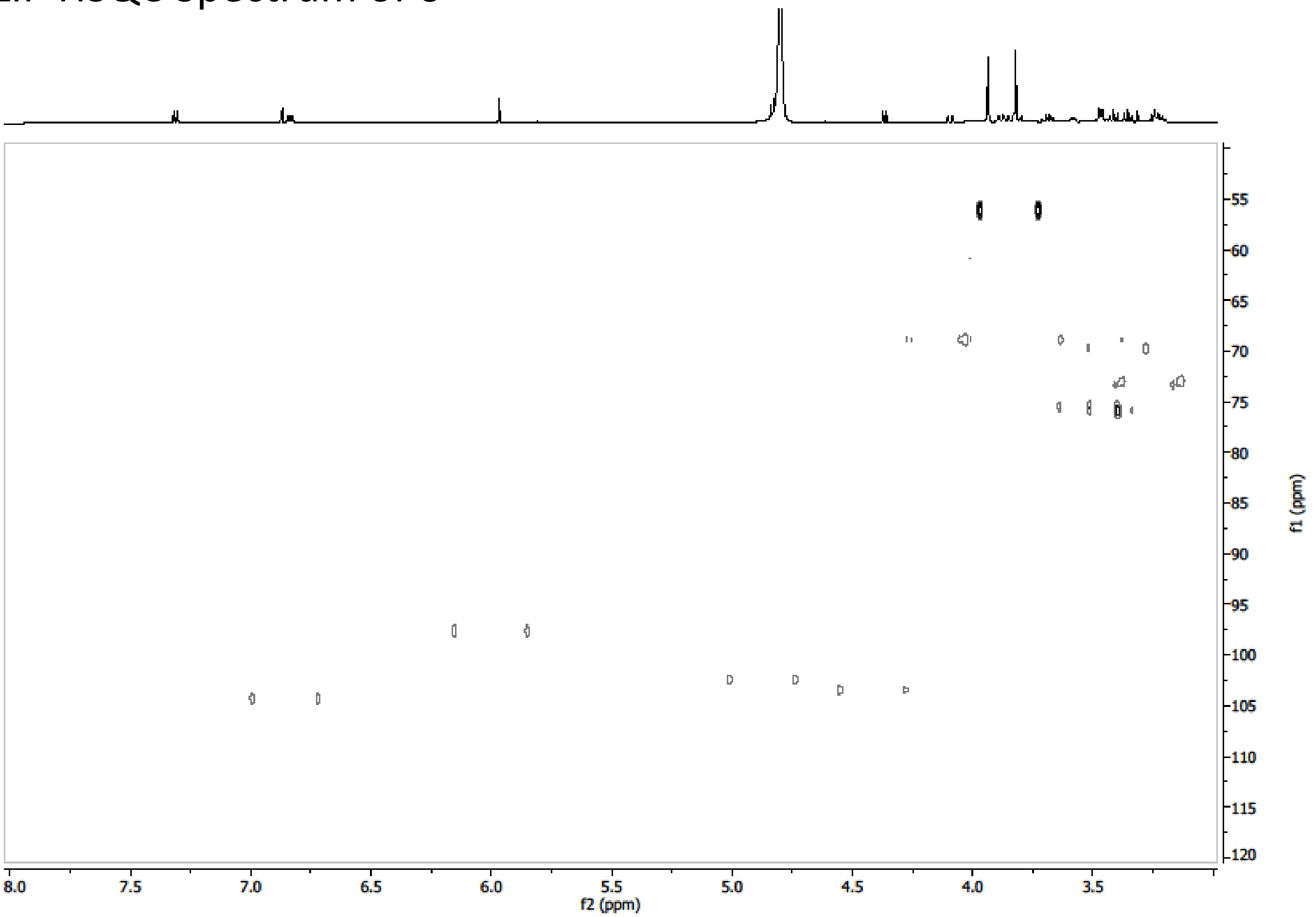

Selective 1D TOCSY Spectrum of **6** irradiation at 4.88 ppm

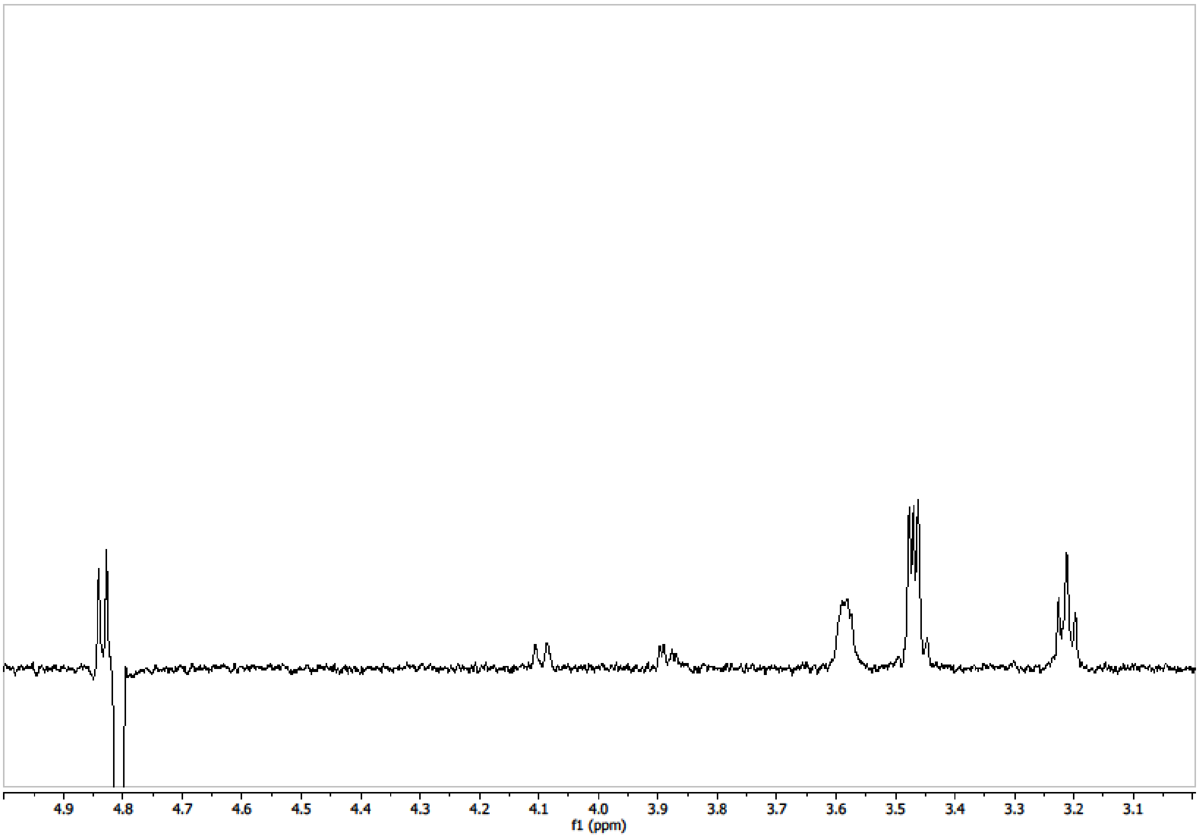

Selective 1D TOCSY Spectrum of **6** irradiation at 4.41 ppm

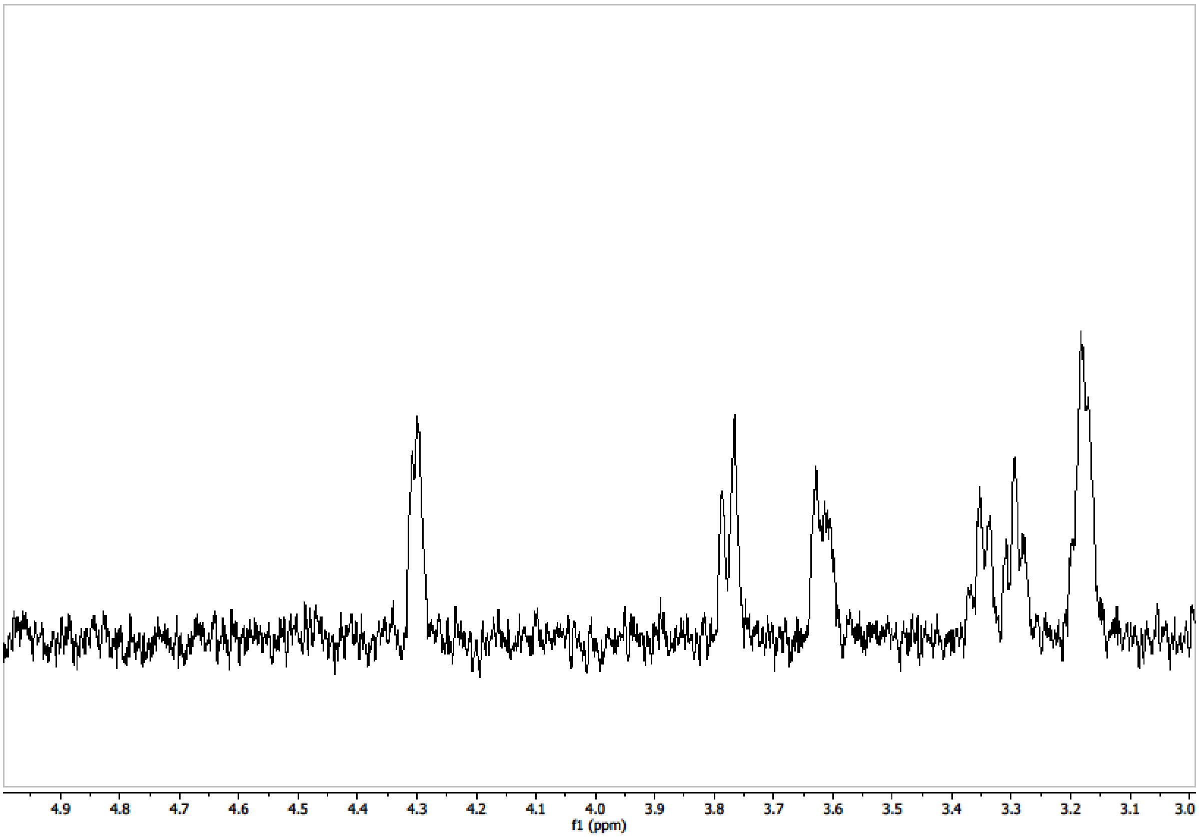

HRMS of **6**

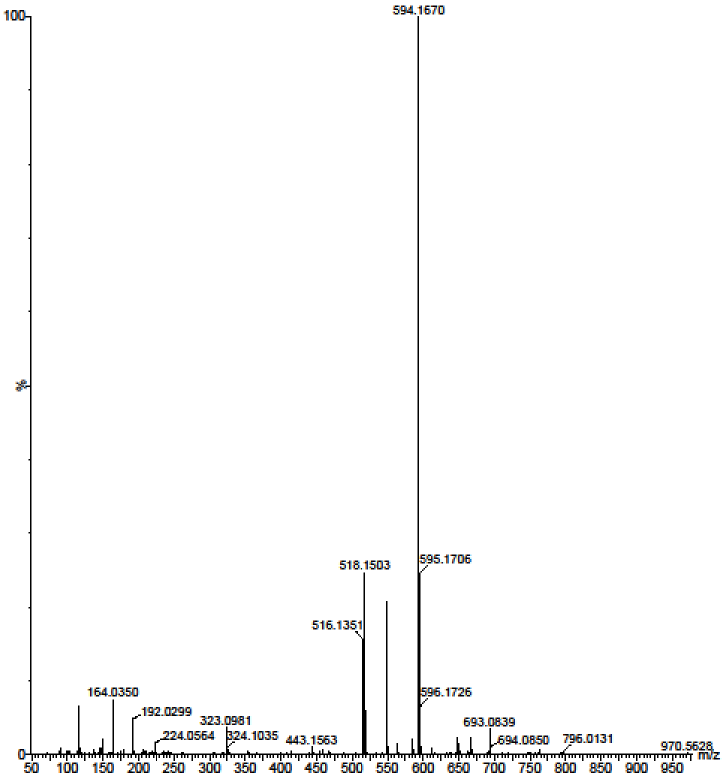

CD Spectrum of **6**

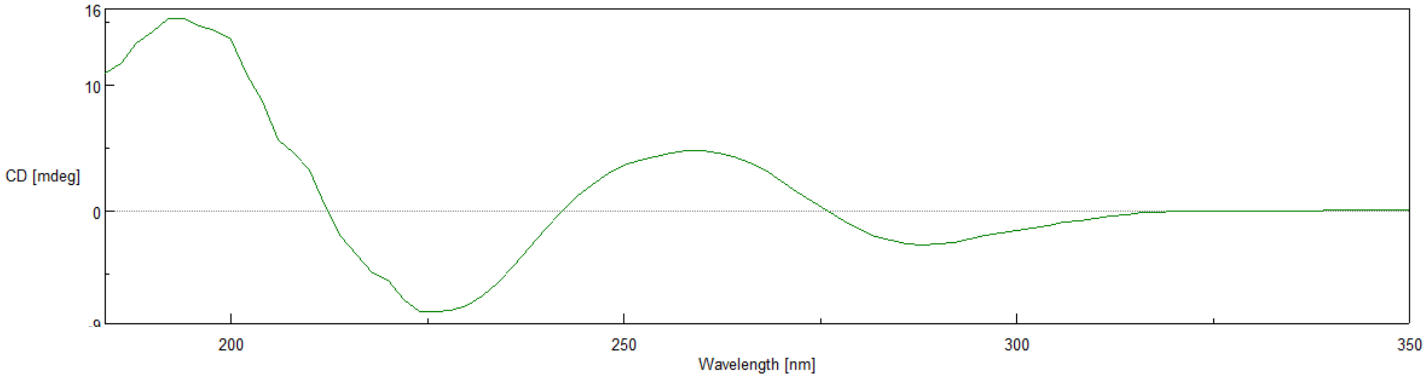

Isolation and structure determination of drought-induced multihexose benzoxazinoids from maize (*Zea mays*)

Supplementary Information SI8:
Structure elucidation of DIBOA-2Glc (7).

Sylvain Sutour[1,*], Cong Van Doan[2,3,4], Pierre Mateo[2], Tobias Züst[5], Ella Raymonde Hartmann[2], Gaétan Glauser[1], Christelle Aurélie Maud Robert[2,3,*]

[1] Neuchâtel Platform of Analytical Chemistry, University of Neuchâtel, 2000 Neuchâtel, Switzerland

[2] Institute of Plant Sciences, University of Bern, 3013 Bern, Switzerland

[3] Oeschger Centre for Climate Change Research (OCCR), University of Bern, 3012 Bern, Switzerland

[4] Plant Physiology Unit, The Department of Life Sciences and Systems Biology of the University of Turin, Via Accademia Albertina 13, 10123 Torino, Italy

[5] Department of Systematic and Evolutionary Botany, University of Zürich, 8008 Zürich, Switzerland

For correspondence:Sutour Sylvain - sylvain.sutour@unine.ch; Christelle A. M. Robert - christelle.robert@unibe.ch

Chemical data for **7**: ^1H NMR (D_2O, 600 MHz) δ 7.58 (1H, dd, J = 7.5, 2.5 Hz, H-5), 7.24 (1H, ddd, J = 7.9, 6.5, 2.5 Hz, H-7), 7.19 (1H, dd, J = 7.5, 6.5 Hz, H-6), 7.18 (1H, d, J = 7.9 Hz, H-8), 5.95 (1H, s, H-2), 4.87 (1H, d, J = 7.9 Hz, H-1'), 4.48 (1H, d, J = 8.0 Hz, H-1''), 4.21 (1H, dd, J = 12.0, 1.5 Hz, H-6'b), 3.92 (1H, dd, J = 12.2, 2.1 Hz, H-6''b), 3.90 (1H, dd, J = 12.0, 5.0 Hz, H-6'a), 3.74 (1H, dd, J = 12.2, 5.9 Hz, H-6''a), 3.68 (1H, ddd, J = 9.8, 5.1, 1.6 Hz, H-5'), 3.54 (1H, m, H-3'), 3.50 (1H, m, H-4'), 3.47 (1H, m, H-3''), 3.42 (1H, m, H-4''), 3.34 (1H, ddd, J = 9.6, 5.9, 2.1 Hz, H-5''), 3.31 (1H, dd, J = 9.1, 8.0 Hz, H-2''), 3.25 (1H, dd, J = 8.9, 7.9 Hz, H-2'); HRESIMS m/z 504.1364 (calcd for C20H26NO14, 504.1353).

^1H NMR Spectrum of **7** (D_2O, 600 MHz)

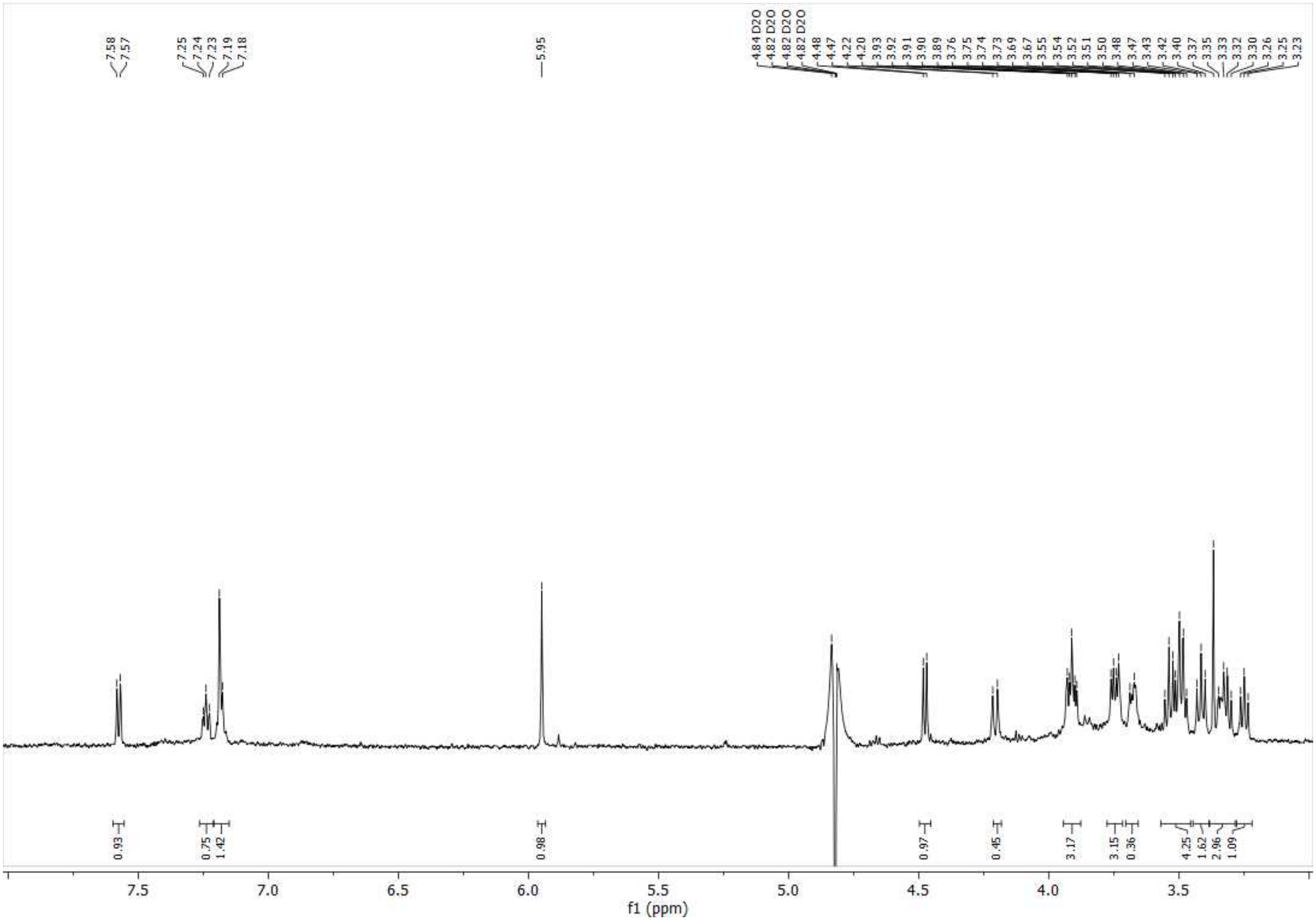

Supplement: Supplementary file 1 — jf3c09141_si_001.pdf [file jf3c09141_si_001.pdf]
